# Supplementary material for: Endogenous Hormone Levels and Transcriptomic Analysis Reveal the Mechanisms of Bulbil Initiation in Pinellia ternata
Source: Int J Mol Sci. 2024 Jun 3;25(11):6149. doi: 10.3390/ijms25116149 (PMC11173086; doi:10.3390/ijms25116149)

Sup.raw data

Calibration curve

Component	Calibration curve	Correlation coefficent R	Weight
Zeatin	y = 24439.76712 x + -285.65501	0.99072	$1/x^2$
IP	y = 8.03214e5 x + 23475.21140	0.99688	$1/x^2$
IPA	y = 5.69500e5 x + 3930.71488	0.99750	$1/x^2$
IAA	y = 17530.42039 x + 2299.36382	0.99676	$1/x^2$
ABA	y = 27169.46155 x + 5110.08090	0.99473	$1/x^2$
SA	y = 22027.20998 x + 1.04861e5	0.99465	$1/x^2$
MESA	y = 7.04570e5 x + -16330.18156	0.99910	$1/x^2$
JA	y = 1782.49601 x + 455.66467	0.99260	$1/x^2$
MEJA	y = 1.39463e5 x + 1015.55113	0.99292	$1/x^2$
TZR	y = 2.92442e5 x + -5169.71987	0.99814	$1/x^2$
IBA	y = 674.30811 x + -3.26572	0.99857	$1/x^2$
5-DS	y =337.20896 x + -387.46192	0.99099	$1/x^2$

IP, isopentenyladenine; SA, salicylic acid; MESA, methyl salicylate; IPA, isopentenyl adenosine; ABA, abscisic acid; JA, jasmonic acid; IAA, indole-3-acetic acid; IBA, indole-3-butyric acid; 5-DS, 5-dexoxystrigol; MeJA, methyl jasmonate; TZR, trans-zeatin nucleoside. SU, the top of the petiole in SB; DU, the top of the petiole in DB.

Sample raw data of acid-alkaline hormones

Sample Name: DU **Vial #:** 10

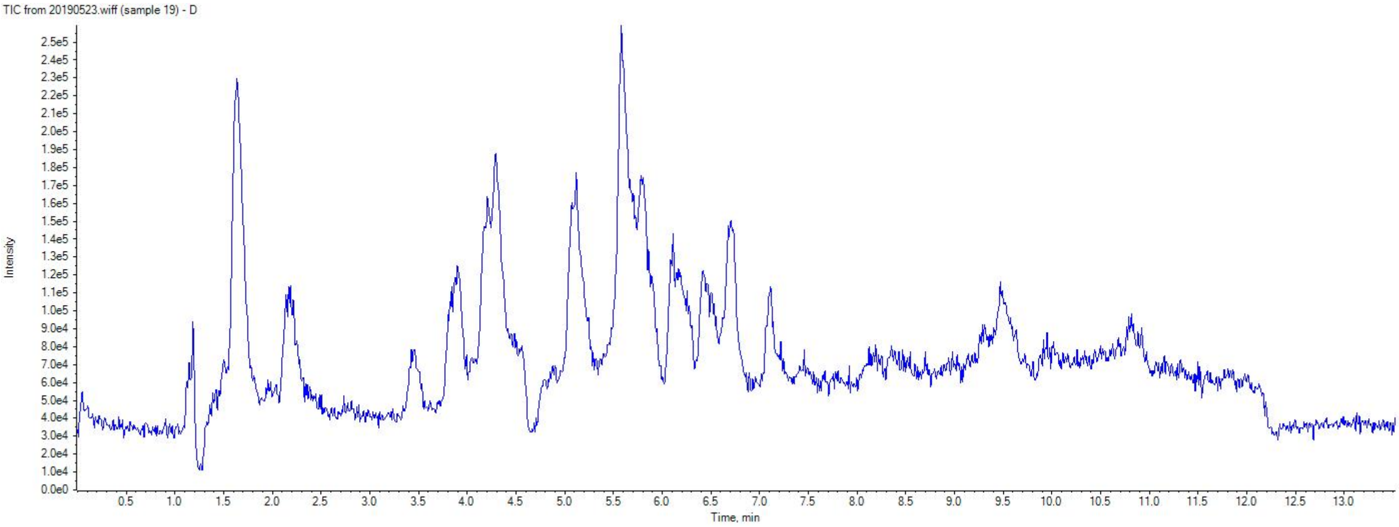

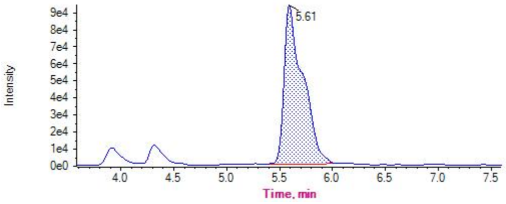

**Compound Name:**	IAA 2 **(**176.2 / 102.9**)**
Expected RT:	5.59
Actual RT:	5.61
Equation:	y = 17530.42039 x + 2299.36382 (r = 0.99676)<br>(weighting: 1 / x^2)
Area Counts:	1.151e6
ISTD Area Counts:	N/A
Amount:	65.55 (ng/mL)

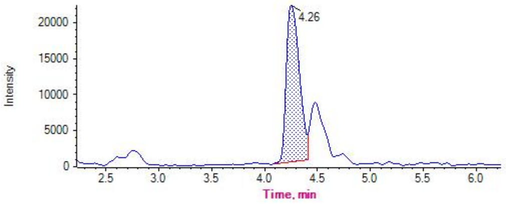

**Compound Name:**	T Zeatin 1 **(**352.3 / 220.2**)**
Expected RT:	4.23
Actual RT:	4.26
Equation:	y = 2.92442e5 x + -5169.71987 (r = 0.99814)<br>(weighting: 1 / x^2)
Area Counts:	1.878e5
ISTD Area Counts:	N/A
Amount:	0.66 (ng/mL)

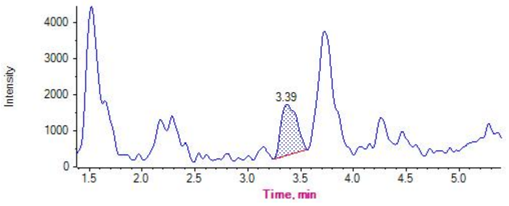

**Compound Name:**	Zeatin 1 **(**220.4 / 136.0**)**
Expected RT:	3.39
Actual RT:	3.39
Equation:	y = 24439.76712 x + -285.65501 (r = 0.99072) (weighting: 1 / x^2)
Area Counts:	1.466e4
ISTD Area Counts:	N/A
Amount:	0.61 (ng/mL)

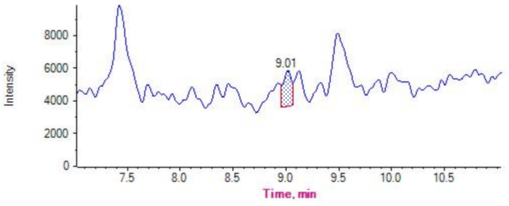

**Compound Name:**	MEJA 2 **(**225.1 / 133.0**)**
Expected RT:	9.03
Actual RT:	9.01
Equation:	y = 1.39463e5 x + 1015.55113 (r = 0.99292) (weighting: 1 / x^2)
Area Counts:	1.135e4
ISTD Area Counts:	N/A
Amount:	0.07 (ng/mL)

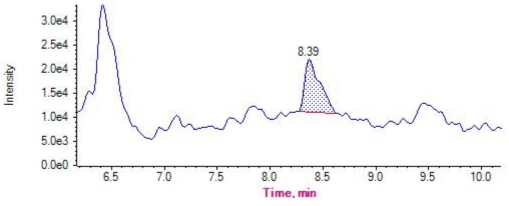

**Compound Name:**	MESA **(**153.0 / 93.1**)**
Expected RT:	8.18
Actual RT:	8.39
Equation:	y = 7.04570e5 x + -16330.18156 (r = 0.99910) (weighting: 1 / x^2)
Area Counts:	1.103e5
ISTD Area Counts:	N/A
Amount:	0.18 (ng/mL)

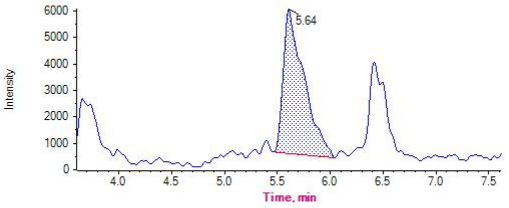

**Compound Name:**	IP 1 **(**204.1 / 136.1**)**
Expected RT:	5.61
Actual RT:	5.64
Equation:	y = 8.03214e5 x + 23475.21140 (r = 0.99688) (weighting: 1 / x^2)
Area Counts:	7.437e4
ISTD Area Counts:	N/A
Amount:	0.06 (ng/mL)

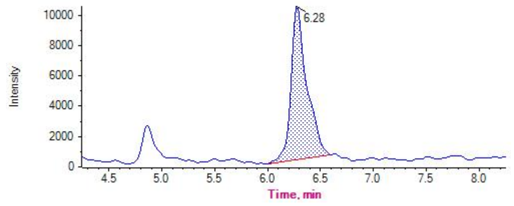

**Compound Name:**	IPA 1 (336.2 / 204.2)
Expected RT:	6.25
Actual RT:	6.28
Equation:	y = 5.69500e5 x + 3930.71488 (r = 0.99750) (weighting: 1 / x^2)
Area Counts:	1.056e5
ISTD Area Counts:	N/A
Amount:	0.18 (ng/mL)

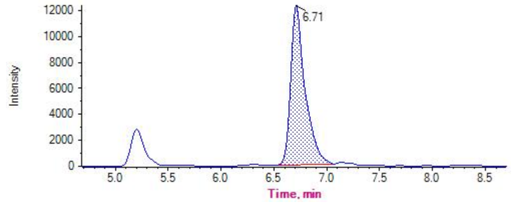

**Compound Name:**	ABA 1 (263.1 / 153.0)
Expected RT:	6.69
Actual RT:	6.71
Equation:	y = 27169.46155 x + 5110.08090 (r = 0.99473) (weighting: 1 / x^2)
Area Counts:	1.162e5
ISTD Area Counts:	N/A
Amount:	4.09 (ng/mL)

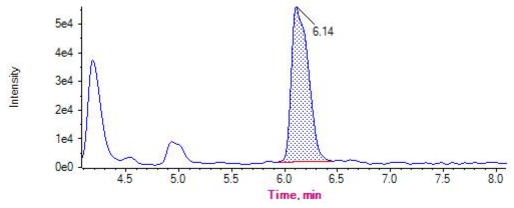

**Compound Name:**	SA 1 (137.0 / 92.9)
Expected RT:	6.09
Actual RT:	6.14
Equation:	y = 22027.20998 x + 1.04861e5 (r = 0.99465) (weighting: 1 / x^2)
Area Counts:	6.000e5
ISTD Area Counts:	N/A
Amount:	22.48 (ng/mL)

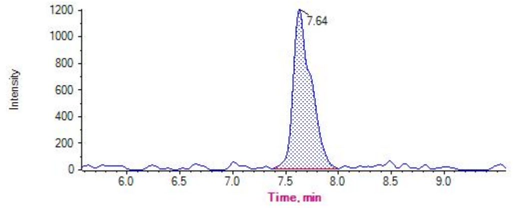

**Compound Name:**	JA 1 (209.2 / 58.9)
Expected RT:	7.58
Actual RT:	7.64
Equation:	y = 1782.49601 x + 455.66467 (r = 0.99260) (weighting: 1 / x^2)
Area Counts:	1.419e4
ISTD Area Counts:	N/A
Amount:	7.70 (ng/mL)

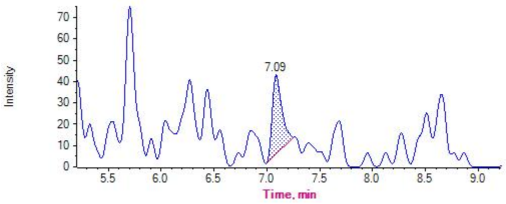

Compound Name:	IBA 1 (202.0 / 116.0)
Expected RT:	7.21
Actual RT:	7.09
Equation:	y = 674.30811 x + -3.26572 (r = 0.99857) (weighting: 1 / x^2)
Area Counts:	2.324e2
ISTD Area Counts:	N/A
Amount:	0.35 (ng/mL)

Sample Name: DU **Vial #:** 10

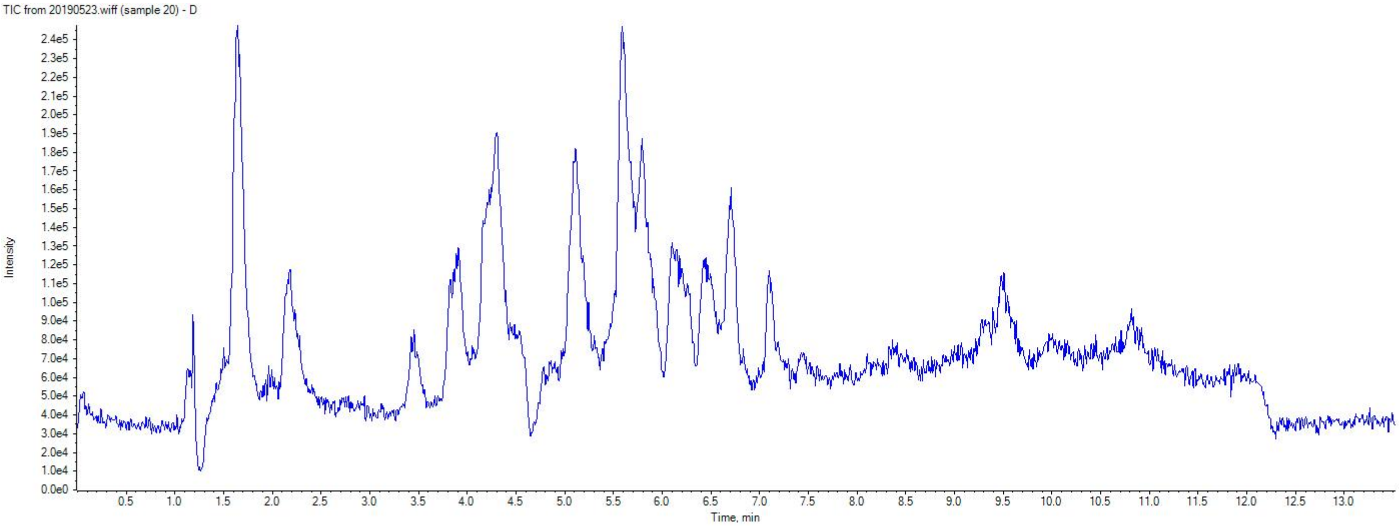

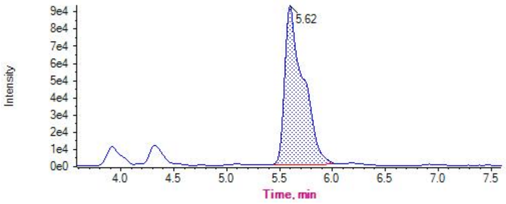

Compound Name:	IAA 2 (176.2 / 102.9)
Expected RT:	5.59
Actual RT:	5.62
Equation:	y = 17530.42039 x + 2299.36382 (r = 0.99676) (weighting: 1 / x^2)
Area Counts:	1.126e6
ISTD Area Counts:	N/A
Amount:	64.10 (ng/mL)

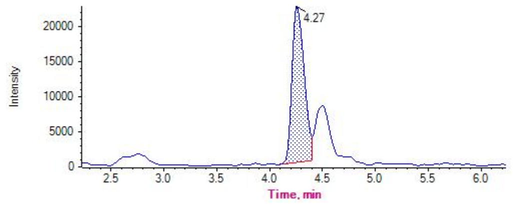

**Compound Name:**	T Zeatin 1 **(**352.3 / 220.2**)**
Expected RT:	4.23
Actual RT:	4.27
Equation:	y = 2.92442e5 x + -5169.71987 (r = 0.99814) (weighting: 1 / x^2)
Area Counts:	1.787e5
ISTD Area Counts:	N/A
Amount:	0.63 (ng/mL)

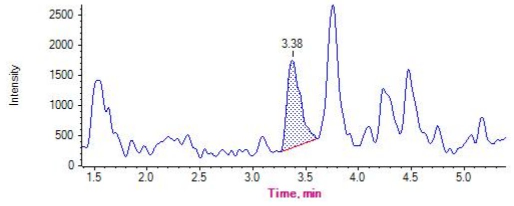

**Compound Name:**	Zeatin 1 **(**220.4 / 136.0**)**
Expected RT:	3.39
Actual RT:	3.38
Equation:	y = 24439.76712 x + -285.65501 (r = 0.99072) (weighting: 1 / x^2)
Area Counts:	1.268e4
ISTD Area Counts:	N/A
Amount:	0.53 (ng/mL)

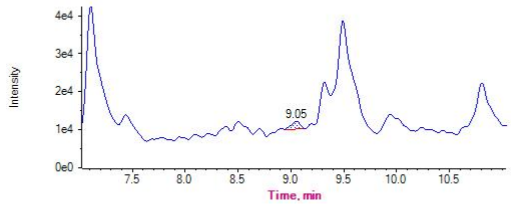

**Compound Name:**	MEJA 2 **(**225.1 / 133.0**)**
Expected RT:	9.03
Actual RT:	9.05
Equation:	y = 1.39463e5 x + 1015.55113 (r = 0.99292) (weighting: 1 / x^2)
Area Counts:	1.152e4
ISTD Area Counts:	N/A
Amount:	0.08 (ng/mL)

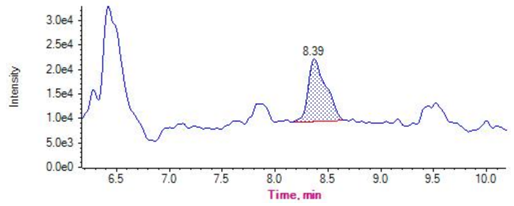

**Compound Name:**	MESA **(**153.0 / 93.1**)**
Expected RT:	8.18
Actual RT:	8.39
Equation:	y = 7.04570e5 x + -16330.18156 (r = 0.99910) (weighting: 1 / x^2)
Area Counts:	1.504e5
ISTD Area Counts:	N/A
Amount:	0.24 (ng/mL)

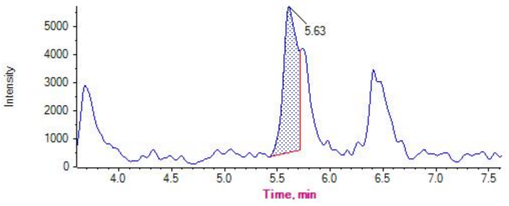

**Compound Name:**	IP 1 (204.1 / 136.1)
Expected RT:	5.61
Actual RT:	5.63
Equation:	y = 8.03214e5 x + 23475.21140 (r = 0.99688) (weighting: 1 / x^2)
Area Counts:	4.703e4
ISTD Area Counts:	N/A
Amount:	0.03 (ng/mL)

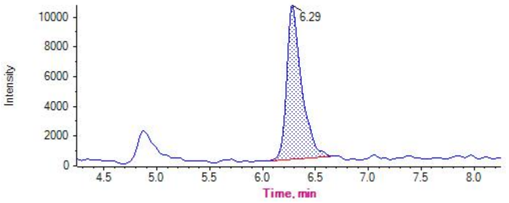

**Compound Name:**	IPA 1 (336.2 / 204.2)
Expected RT:	6.25
Actual RT:	6.29
Equation:	y = 5.69500e5 x + 3930.71488 (r = 0.99750) (weighting: 1 / x^2)
Area Counts:	1.017e5
ISTD Area Counts:	N/A
Amount:	0.17 (ng/mL)

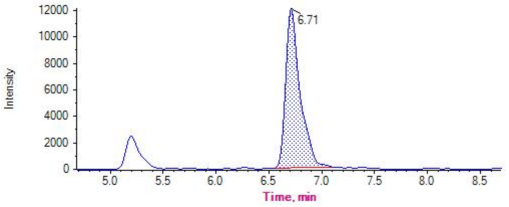

**Compound Name:**	ABA 1 (263.1 / 153.0)
Expected RT:	6.69
Actual RT:	6.71
Equation:	y = 27169.46155 x + 5110.08090 (r = 0.99473) (weighting: 1 / x^2)
Area Counts:	1.136e5
ISTD Area Counts:	N/A
Amount:	3.99 (ng/mL)

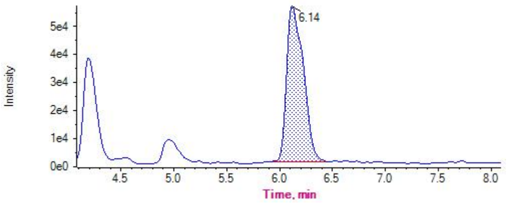

**Compound Name:**	SA 1 (137.0 / 92.9)
Expected RT:	6.09
Actual RT:	6.14
Equation:	y = 22027.20998 x + 1.04861e5 (r = 0.99465) (weighting: 1 / x^2)
Area Counts:	6.143e5
ISTD Area Counts:	N/A
Amount:	23.13 (ng/mL)

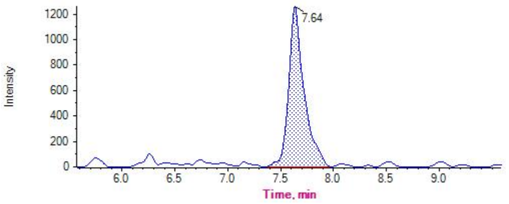

**Compound Name:**	JA 1 **(**209.2 / 58.9**)**
Expected RT:	7.58
Actual RT:	7.64
Equation:	y = 1782.49601 x + 455.66467 (r = 0.99260) (weighting: 1 / x^2)
Area Counts:	1.277e4
ISTD Area Counts:	N/A
Amount:	6.91 (ng/mL)

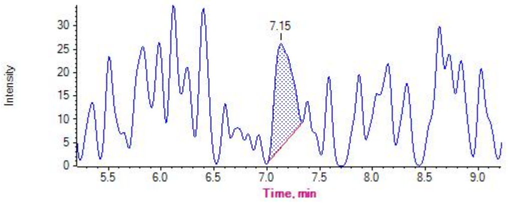

**Compound Name:**	IBA 1 **(**202.0 / 116.0**)**
Expected RT:	7.21
Actual RT:	7.15
Equation:	y = 674.30811 x + -3.26572 (r = 0.99857) (weighting: 1 / x^2)
Area Counts:	2.432e2
ISTD Area Counts:	N/A
Amount:	0.37 (ng/mL)

Sample Name: DU **Vial #:** 10

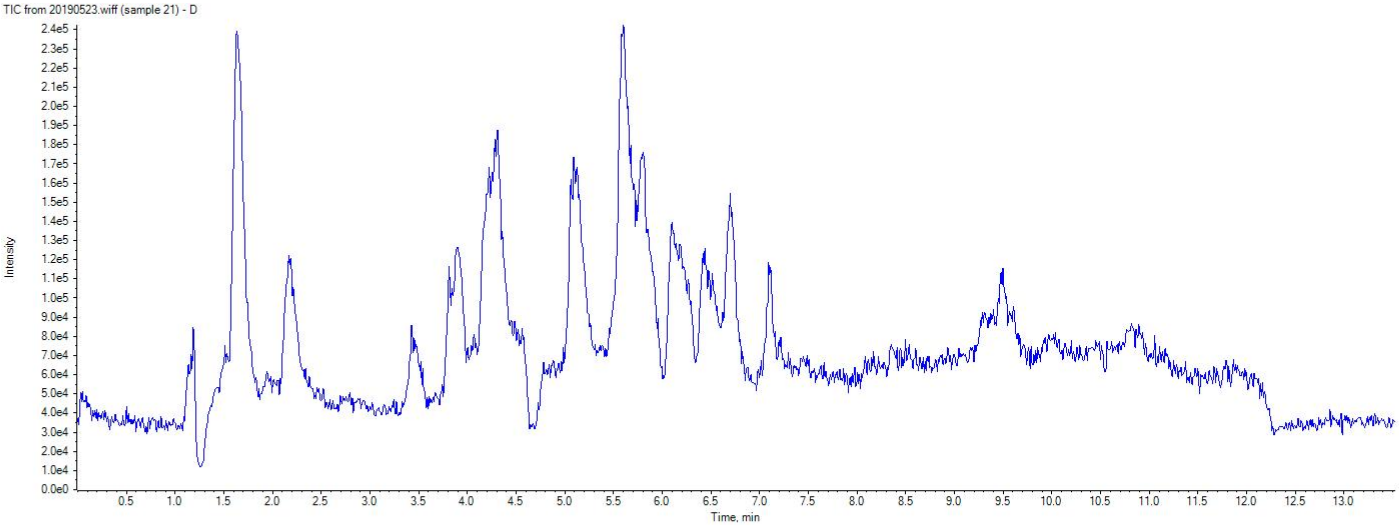

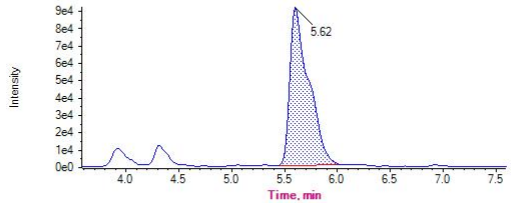

**Compound Name:**	IAA 2 **(**176.2 / 102.9**)**
Expected RT:	5.59
Actual RT:	5.62
Equation:	y = 17530.42039 x + 2299.36382 (r = 0.99676) (weighting: 1 / x^2)
Area Counts:	1.133e6
ISTD Area Counts:	N/A
Amount:	64.50 (ng/mL)

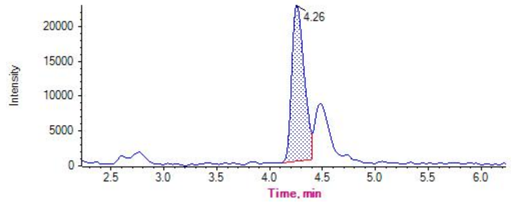

**Compound Name:**	T Zeatin 1 **(**352.3 / 220.2**)**
Expected RT:	4.23
Actual RT:	4.26
Equation:	y = 2.92442e5 x + -5169.71987 (r = 0.99814) (weighting: 1 / x^2)
Area Counts:	1.827e5
ISTD Area Counts:	N/A
Amount:	0.64 (ng/mL)

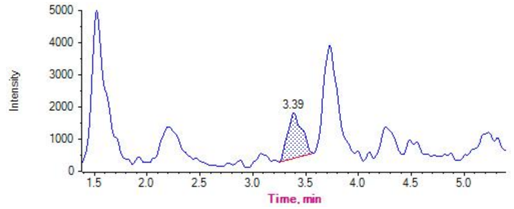

**Compound Name:**	Zeatin 1 **(**220.4 / 136.0**)**
Expected RT:	3.39
Actual RT:	3.39
Equation:	y = 24439.76712 x + -285.65501 (r = 0.99072) (weighting: 1 / x^2)
Area Counts:	1.326e4
ISTD Area Counts:	N/A
Amount:	0.55 (ng/mL)

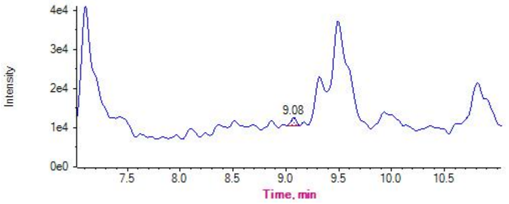

**Compound Name:**	MEJA 2 **(**225.1 / 133.0**)**
Expected RT:	9.03
Actual RT:	9.08
Equation:	y = 1.39463e5 x + 1015.55113 (r = 0.99292)<br>(weighting: 1 / x^2)
Area Counts:	7.266e3
ISTD Area Counts:	N/A
Amount:	0.04 (ng/mL)

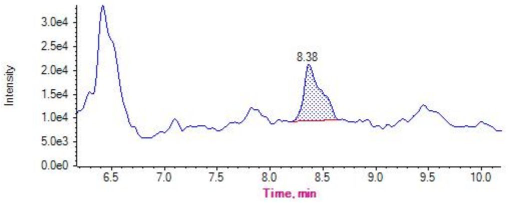

**Compound Name:**	MESA **(**153.0 / 93.1**)**
Expected RT:	8.18
Actual RT:	8.38
Equation:	y = 7.04570e5 x + -16330.18156 (r = 0.99910)<br>(weighting: 1 / x^2)
Area Counts:	1.326e5
ISTD Area Counts:	N/A
Amount:	0.21 (ng/mL)

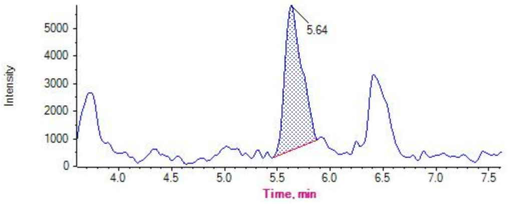

**Compound Name:**	IP 1 **(**204.1 / 136.1**)**
Expected RT:	5.61
Actual RT:	5.64
Equation:	y = 8.03214e5 x + 23475.21140 (r = 0.99688)<br>(weighting: 1 / x^2)
Area Counts:	5.865e4
ISTD Area Counts:	N/A
Amount:	0.04 (ng/mL)

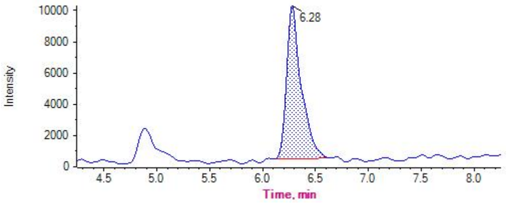

**Compound Name:**	IPA 1 **(**336.2 / 204.2**)**
Expected RT:	6.25
Actual RT:	6.28
Equation:	y = 5.69500e5 x + 3930.71488 (r = 0.99750)<br>(weighting: 1 / x^2)
Area Counts:	9.456e4
ISTD Area Counts:	N/A
Amount:	0.16 (ng/mL)

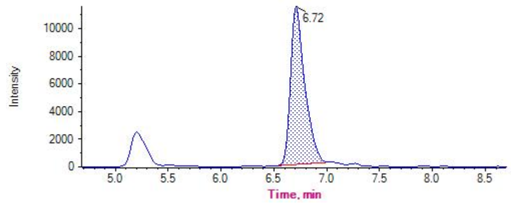

**Compound Name:**	ABA 1 **(**263.1 / 153.0**)**
Expected RT:	6.69
Actual RT:	6.72
Equation:	y = 27169.46155 x + 5110.08090 (r = 0.99473) (weighting: 1 / x^2)
Area Counts:	1.070e5
ISTD Area Counts:	N/A
Amount:	3.75 (ng/mL)

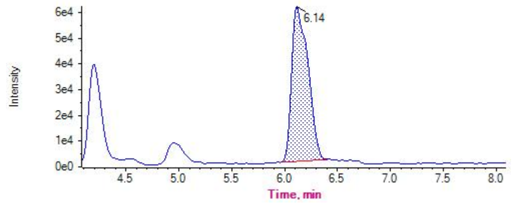

**Compound Name:**	SA 1 **(**137.0 / 92.9**)**
Expected RT:	6.09
Actual RT:	6.14
Equation:	y = 22027.20998 x + 1.04861e5 (r = 0.99465) (weighting: 1 / x^2)
Area Counts:	6.495e5
ISTD Area Counts:	N/A
Amount:	24.73 (ng/mL)

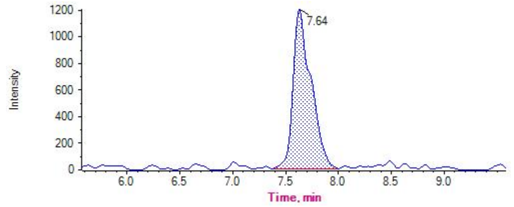

**Compound Name:**	JA 1 **(**209.2 / 58.9**)**
Expected RT:	7.58
Actual RT:	7.64
Equation:	y = 1782.49601 x + 455.66467 (r = 0.99260) (weighting: 1 / x^2)
Area Counts:	1.302e4
ISTD Area Counts:	N/A
Amount:	7.05 (ng/mL)

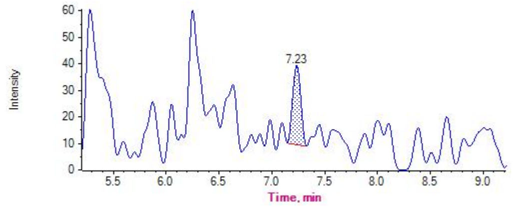

**Compound Name:**	IBA 1 **(**202.0 / 116.0**)**
Expected RT:	7.21
Actual RT:	7.23
Equation:	y = 674.30811 x + -3.26572 (r = 0.99857) (weighting: 1 / x^2)
Area Counts:	1.590e2
ISTD Area Counts:	N/A
Amount:	0.24 (ng/mL)

Sample Name: SU **Vial #:** 11

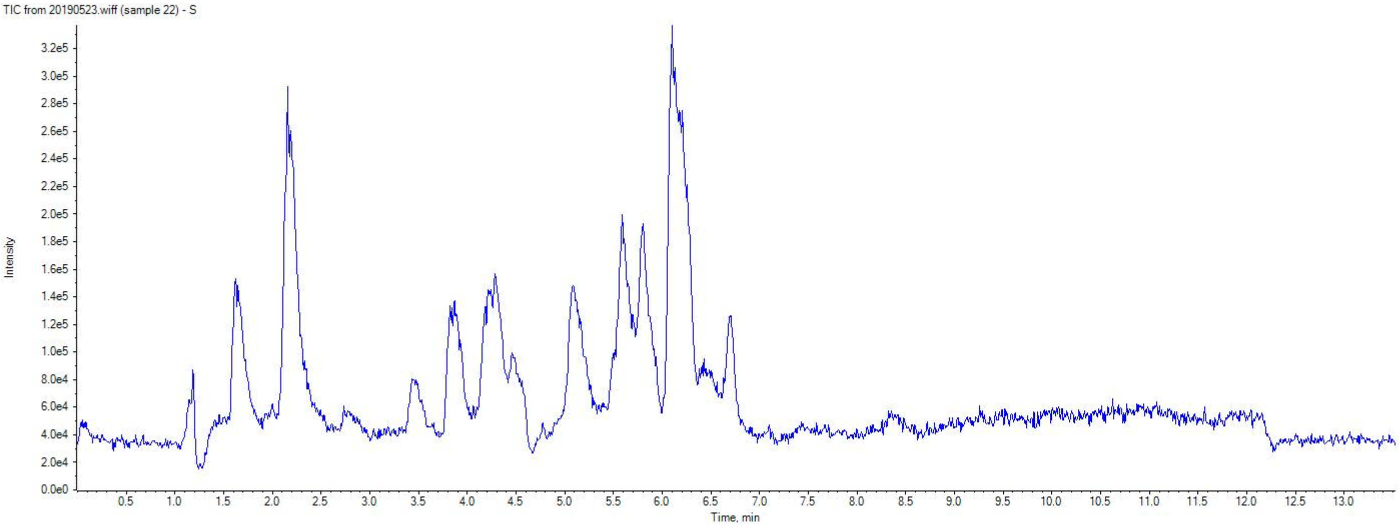

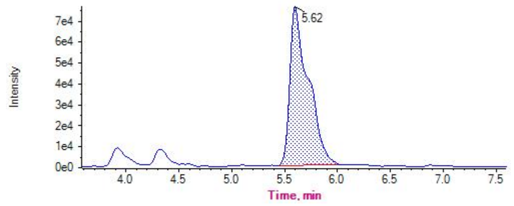

**Compound Name:**	IAA 2 **(**176.2 / 102.9**)**
Expected RT:	5.59
Actual RT:	5.62
Equation:	y = 17530.42039 x + 2299.36382 (r = 0.99676) (weighting: 1 / x^2)
Area Counts:	9.223e5
ISTD Area Counts:	N/A
Amount:	52.48 (ng/mL)

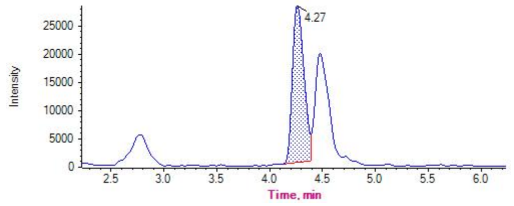

**Compound Name:**	T Zeatin 1 **(**352.3 / 220.2**)**
Expected RT:	4.23
Actual RT:	4.27
Equation:	y = 2.92442e5 x + -5169.71987 (r = 0.99814) (weighting: 1 / x^2)
Area Counts:	2.102e5
ISTD Area Counts:	N/A
Amount:	0.74 (ng/mL)

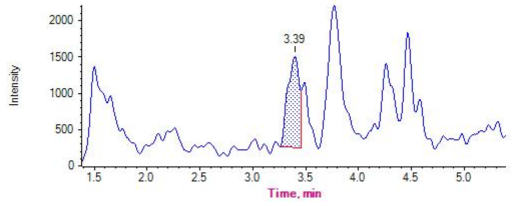

**Compound Name:**	Zeatin 1 **(**220.4 / 136.0**)**
Expected RT:	3.39
Actual RT:	3.39
Equation:	y = 24439.76712 x + -285.65501 (r = 0.99072) (weighting: 1 / x^2)
Area Counts:	9.356e3
ISTD Area Counts:	N/A
Amount:	0.39 (ng/mL)

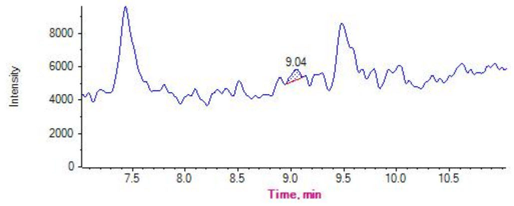

**Compound Name:**	MEJA 2 **(**225.1 / 133.0**)**
Expected RT:	9.03
Actual RT:	9.04
Equation:	y = 1.39463e5 x + 1015.55113 (r = 0.99292) (weighting: 1 / x^2)
Area Counts:	3.584e3
ISTD Area Counts:	N/A
Amount:	0.02 (ng/mL)

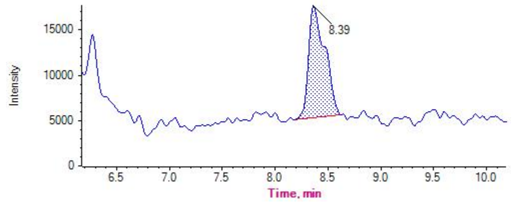

**Compound Name:**	MESA **(**153.0 / 93.1**)**
Expected RT:	8.18
Actual RT:	8.39
Equation:	y = 7.04570e5 x + -16330.18156 (r = 0.99910) (weighting: 1 / x^2)
Area Counts:	1.327e5
ISTD Area Counts:	N/A
Amount:	0.21 (ng/mL)

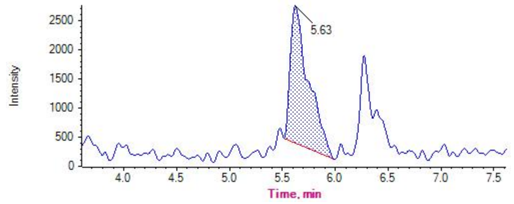

**Compound Name:**	IP 1 **(**204.1 / 136.1**)**
Expected RT:	5.61
Actual RT:	5.63
Equation:	y = 8.03214e5 x + 23475.21140 (r = 0.99688) (weighting: 1 / x^2)
Area Counts:	2.838e4
ISTD Area Counts:	N/A
Amount:	0.01 (ng/mL)

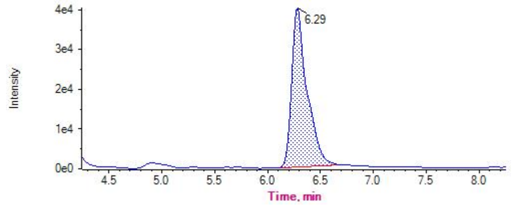

**Compound Name:**	IPA 1 **(**336.2 / 204.2**)**
Expected RT:	6.25
Actual RT:	6.29
Equation:	y = 5.69500e5 x + 3930.71488 (r = 0.99750) (weighting: 1 / x^2)
Area Counts:	4.020e5
ISTD Area Counts:	N/A
Amount:	0.70 (ng/mL)

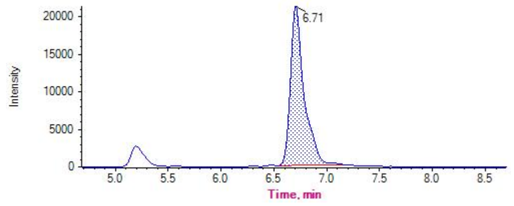

**Compound Name:**	ABA 1 **(**263.1 / 153.0**)**
Expected RT:	6.69
Actual RT:	6.71
Equation:	y = 27169.46155 x + 5110.08090 (r = 0.99473) (weighting: 1 / x^2)
Area Counts:	1.902e5
ISTD Area Counts:	N/A
Amount:	6.81 (ng/mL)

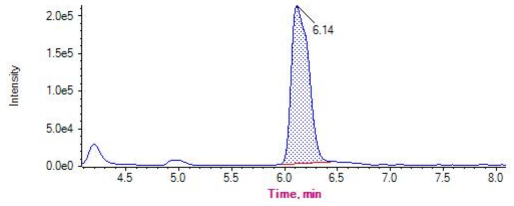

**Compound Name:**	SA 1 **(**137.0 / 92.9**)**
Expected RT:	6.09
Actual RT:	6.14
Equation:	y = 22027.20998 x + 1.04861e5 (r = 0.99465) (weighting: 1 / x^2)
Area Counts:	2.384e6
ISTD Area Counts:	N/A
Amount:	103.48 (ng/mL)

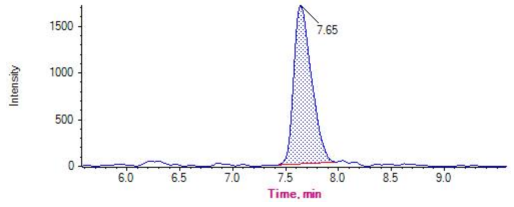

**Compound Name:**	JA 1 **(**209.2 / 58.9**)**
Expected RT:	7.58
Actual RT:	7.65
Equation:	y = 1782.49601 x + 455.66467 (r = 0.99260) (weighting: 1 / x^2)
Area Counts:	1.982e4
ISTD Area Counts:	N/A
Amount:	10.86 (ng/mL)

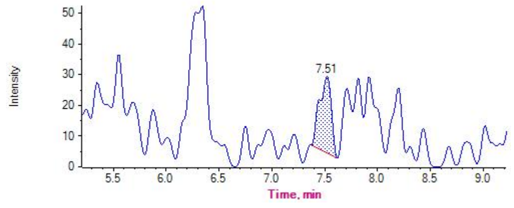

**Compound Name:**	IBA 1 **(**202.0 / 116.0**)**
Expected RT:	7.21
Actual RT:	7.51
Equation:	y = 674.30811 x + -3.26572 (r = 0.99857) (weighting: 1 / x^2)
Area Counts:	1.935e2
ISTD Area Counts:	N/A
Amount:	0.29 (ng/mL)

Sample Name: SU **Vial #:** 11

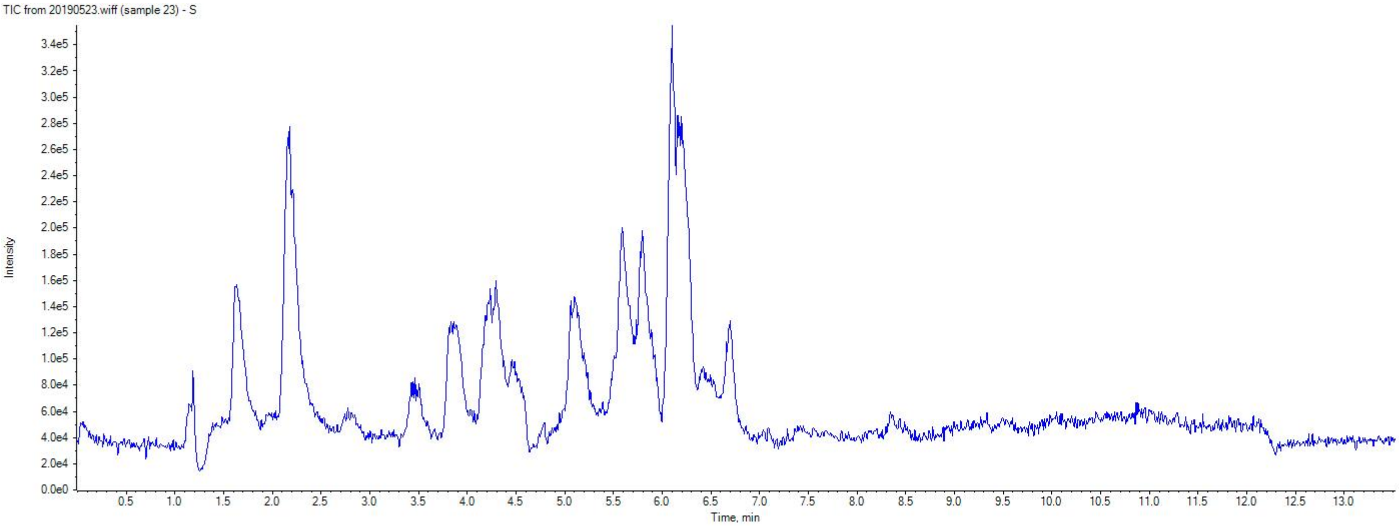

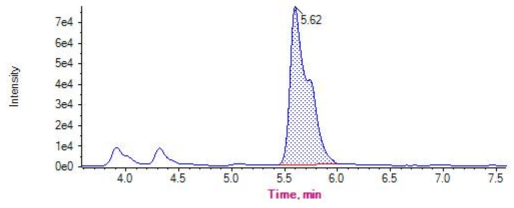

Compound Name:	IAA 2 (176.2 / 102.9)
Expected RT:	5.59
Actual RT:	5.62
Equation:	y = 17530.42039 x + 2299.36382 (r = 0.99676)<br>(weighting: 1 / x^2)
Area Counts:	9.194e5
ISTD Area Counts:	N/A
Amount:	52.31 (ng/mL)

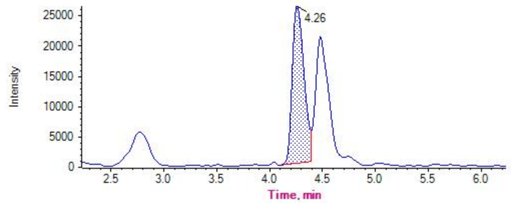

Compound Name:	T Zeatin 1 (352.3 / 220.2)
Expected RT:	4.23
Actual RT:	4.26
Equation:	y = 2.92442e5 x + -5169.71987 (r = 0.99814)<br>(weighting: 1 / x^2)
Area Counts:	2.007e5
ISTD Area Counts:	N/A
Amount:	0.70 (ng/mL)

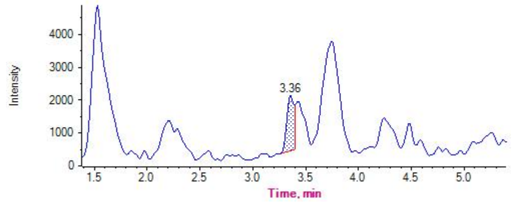

Compound Name:	Zeatin 1 (220.4 / 136.0)
Expected RT:	3.39
Actual RT:	3.36
Equation:	y = 24439.76712 x + -285.65501 (r = 0.99072)<br>(weighting: 1 / x^2)
Area Counts:	7.663e3
ISTD Area Counts:	N/A
Amount:	0.33 (ng/mL)

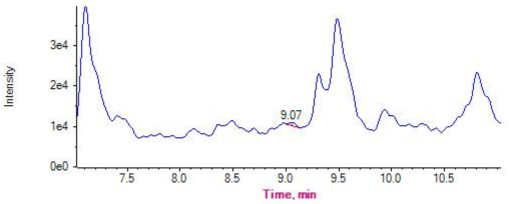

Compound Name:	MEJA 2 (225.1 / 133.0)
Expected RT:	9.03
Actual RT:	9.07
Equation:	y = 1.39463e5 x + 1015.55113 (r = 0.99292) (weighting: 1 / x^2)
Area Counts:	3.822e3
ISTD Area Counts:	N/A
Amount:	0.02 (ng/mL)

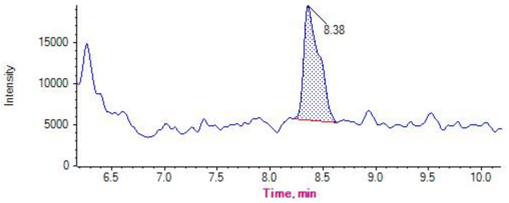

Compound Name:	MESA (153.0 / 93.1)
Expected RT:	8.18
Actual RT:	8.38
Equation:	y = 7.04570e5 x + -16330.18156 (r = 0.99910) (weighting: 1 / x^2)
Area Counts:	1.398e5
ISTD Area Counts:	N/A
Amount:	0.22 (ng/mL)

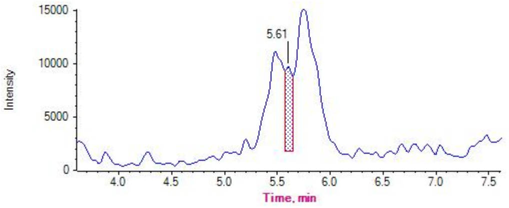

Compound Name:	IP 1 (204.1 / 136.1)
Expected RT:	5.61
Actual RT:	5.61
Equation:	y = 8.03214e5 x + 23475.21140 (r = 0.99688) (weighting: 1 / x^2)
Area Counts:	3.503e4
ISTD Area Counts:	N/A
Amount:	0.01 (ng/mL)

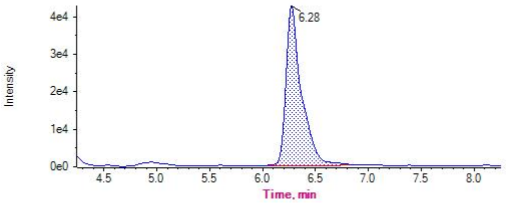

Compound Name:	IPA 1 (336.2 / 204.2)
Expected RT:	6.25
Actual RT:	6.28
Equation:	y = 5.69500e5 x + 3930.71488 (r = 0.99750) (weighting: 1 / x^2)
Area Counts:	4.355e5
ISTD Area Counts:	N/A
Amount:	0.76 (ng/mL)

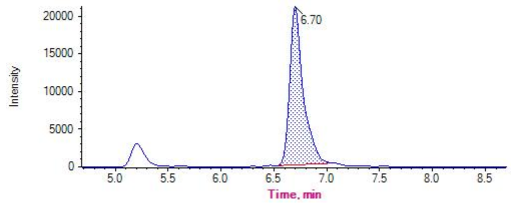

**Compound Name:**	ABA 1 **(**263.1 / 153.0**)**
Expected RT:	6.69
Actual RT:	6.70
Equation:	y = 27169.46155 x + 5110.08090 (r = 0.99473) (weighting: 1 / x^2)
Area Counts:	1.868e5
ISTD Area Counts:	N/A
Amount:	6.69 (ng/mL)

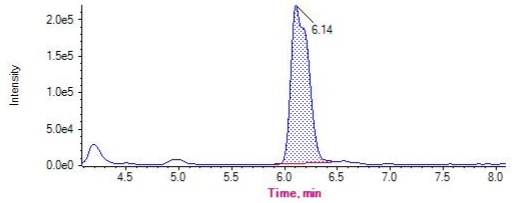

**Compound Name:**	SA 1 **(**137.0 / 92.9**)**
Expected RT:	6.09
Actual RT:	6.14
Equation:	y = 22027.20998 x + 1.04861e5 (r = 0.99465) (weighting: 1 / x^2)
Area Counts:	2.470e6
ISTD Area Counts:	N/A
Amount:	107.36 (ng/mL)

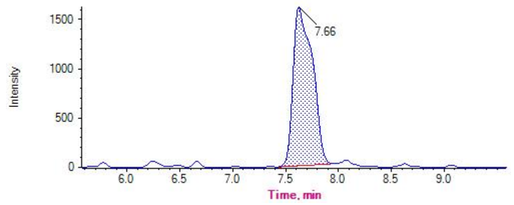

**Compound Name:**	JA 1 **(**209.2 / 58.9**)**
Expected RT:	7.58
Actual RT:	7.66
Equation:	y = 1782.49601 x + 455.66467 (r = 0.99260) (weighting: 1 / x^2)
Area Counts:	2.043e4
ISTD Area Counts:	N/A
Amount:	11.21 (ng/mL)

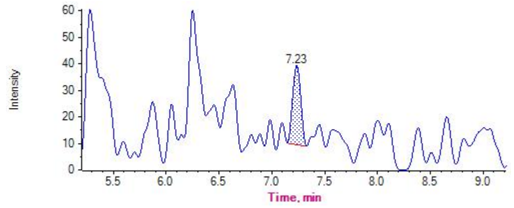

**Compound Name:**	IBA 1 **(**202.0 / 116.0**)**
Expected RT:	7.21
Actual RT:	7.23
Equation:	y = 674.30811 x + -3.26572 (r = 0.99857) (weighting: 1 / x^2)
Area Counts:	1.690e2
ISTD Area Counts:	N/A
Amount:	0.26 (ng/mL)

Sample Name: SU **Vial #:** 11

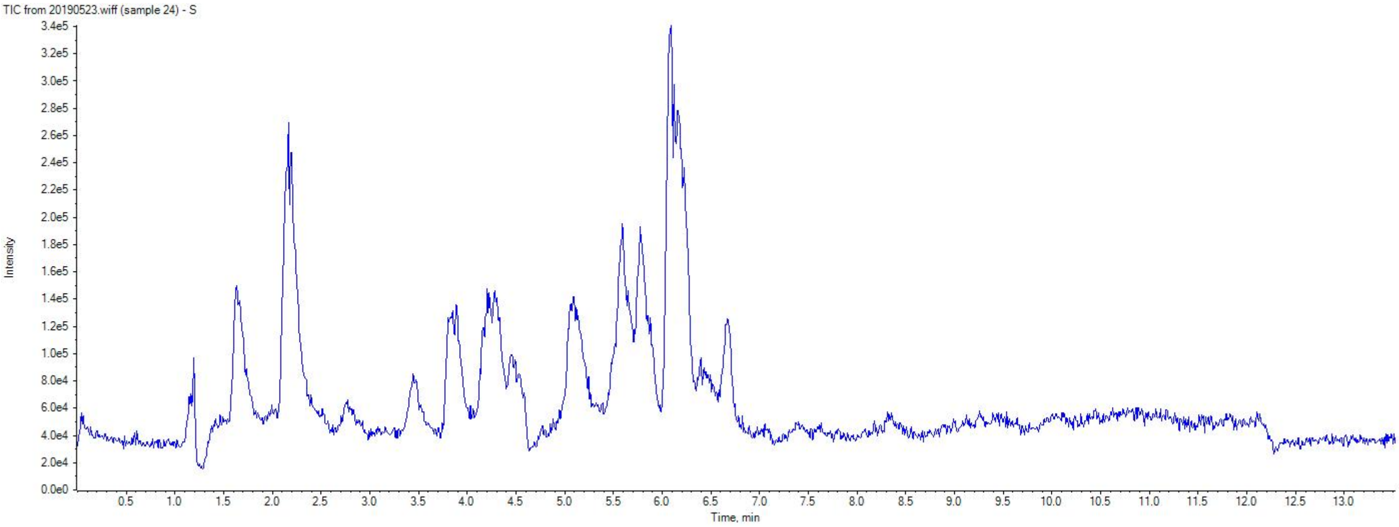

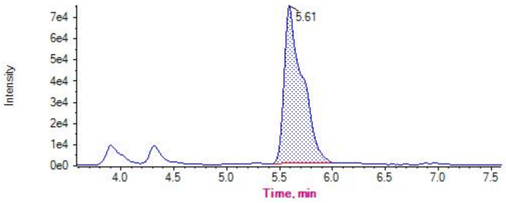

**Compound Name:**	IAA 2 **(**176.2 / 102.9**)**
Expected RT:	5.59
Actual RT:	5.61
Equation:	y = 17530.42039 x + 2299.36382 (r = 0.99676) (weighting: 1 / x^2)
Area Counts:	8.928e5
ISTD Area Counts:	N/A
Amount:	50.80 (ng/mL)

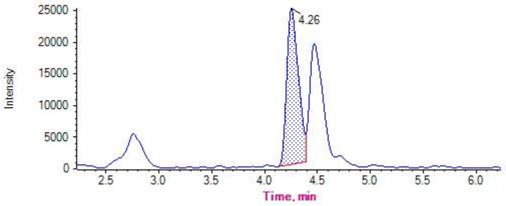

**Compound Name:**	T Zeatin 1 **(**352.3 / 220.2**)**
Expected RT:	4.23
Actual RT:	4.26
Equation:	y = 2.92442e5 x + -5169.71987 (r = 0.99814) (weighting: 1 / x^2)
Area Counts:	1.889e5
ISTD Area Counts:	N/A
Amount:	0.66 (ng/mL)

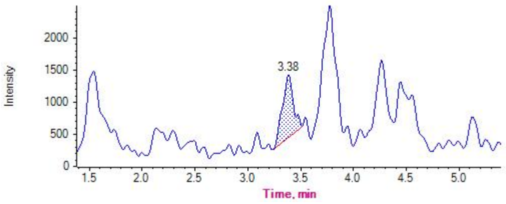

**Compound Name:**	Zeatin 1 **(**220.4 / 136.0**)**
Expected RT:	3.39
Actual RT:	3.38
Equation:	y = 24439.76712 x + -285.65501 (r = 0.99072) (weighting: 1 / x^2)
Area Counts:	7.048e3
ISTD Area Counts:	N/A
Amount:	0.30 (ng/mL)

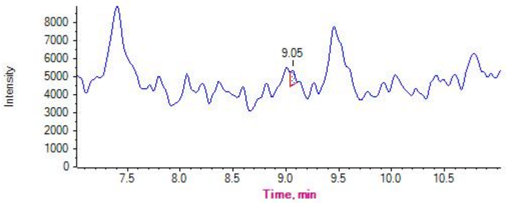

**Compound Name:**	MEJA 2 **(**225.1 / 133.0**)**
Expected RT:	9.03
Actual RT:	9.05
Equation:	y = 1.39463e5 x + 1015.55113 (r = 0.99292) (weighting: 1 / x^2)
Area Counts:	2.534e3
ISTD Area Counts:	N/A
Amount:	0.01 (ng/mL)

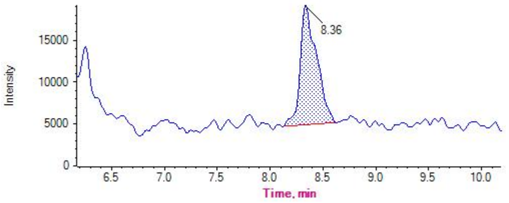

**Compound Name:**	MESA **(**153.0 / 93.1**)**
Expected RT:	8.18
Actual RT:	8.36
Equation:	y = 7.04570e5 x + -16330.18156 (r = 0.99910) (weighting: 1 / x^2)
Area Counts:	1.548e5
ISTD Area Counts:	N/A
Amount:	0.24 (ng/mL)

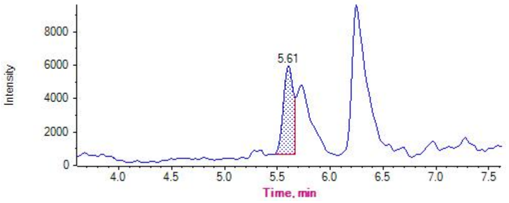

**Compound Name:**	IP 1 **(**204.1 / 136.1**)**
Expected RT:	5.61
Actual RT:	5.61
Equation:	y = 8.03214e5 x + 23475.21140 (r = 0.99688) (weighting: 1 / x^2)
Area Counts:	3.443e4
ISTD Area Counts:	N/A
Amount:	0.01 (ng/mL)

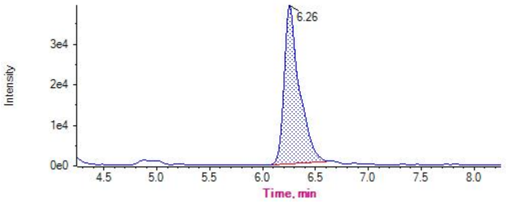

**Compound Name:**	IPA 1 **(**336.2 / 204.2**)**
Expected RT:	6.25
Actual RT:	6.26
Equation:	y = 5.69500e5 x + 3930.71488 (r = 0.99750) (weighting: 1 / x^2)
Area Counts:	3.895e5
ISTD Area Counts:	N/A
Amount:	0.68 (ng/mL)

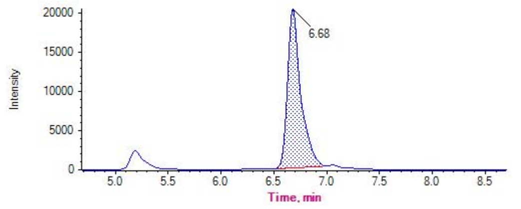

**Compound Name:**	ABA 1 **(**263.1 / 153.0**)**
Expected RT:	6.69
Actual RT:	6.68
Equation:	y = 27169.46155 x + 5110.08090 (r = 0.99473) (weighting: 1 / x^2)
Area Counts:	1.771e5
ISTD Area Counts:	N/A
Amount:	6.33 (ng/mL)

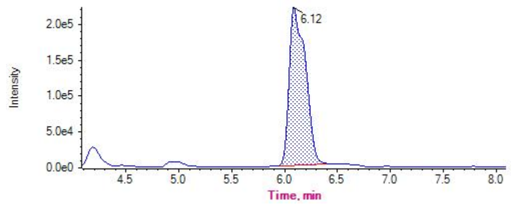

**Compound Name:**	SA 1 **(**137.0 / 92.9**)**
Expected RT:	6.09
Actual RT:	6.12
Equation:	y = 22027.20998 x + 1.04861e5 (r = 0.99465) (weighting: 1 / x^2)
Area Counts:	2.359e6
ISTD Area Counts:	N/A
Amount:	102.34 (ng/mL)

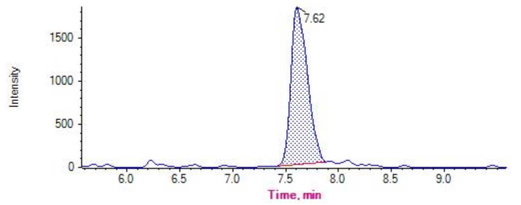

**Compound Name:**	JA 1 **(**209.2 / 58.9**)**
Expected RT:	7.58
Actual RT:	7.62
Equation:	y = 1782.49601 x + 455.66467 (r = 0.99260) (weighting: 1 / x^2)
Area Counts:	1.988e4
ISTD Area Counts:	N/A
Amount:	10.90 (ng/mL)

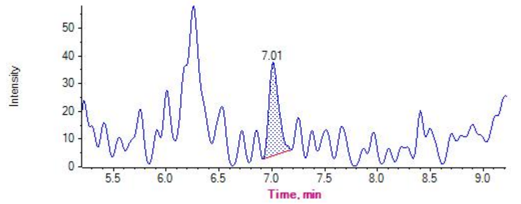

**Compound Name:**	IBA 1 **(**202.0 / 116.0**)**
Expected RT:	7.21
Actual RT:	7.01
Equation:	y = 674.30811 x + -3.26572 (r = 0.99857) (weighting: 1 / x^2)
Area Counts:	2.146e2
ISTD Area Counts:	N/A
Amount:	0.32 (ng/mL)

IAA

0.1

RT (Exp. RT): 5.63 (5.59) min

Calculated Conc: 0.09 ng/mL

Area: 3.948e3

Sample Type: (Standard)

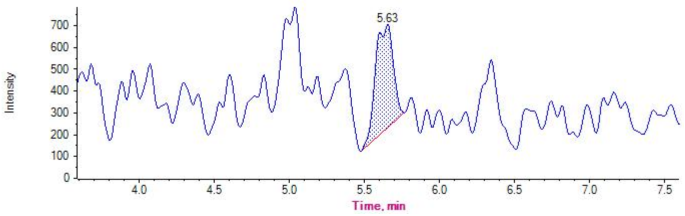

0.2

RT (Exp. RT): 5.60 (5.59) min

Calculated Conc: 0.18 ng/mL

Area: 5.496e3

Sample Type: (Standard)

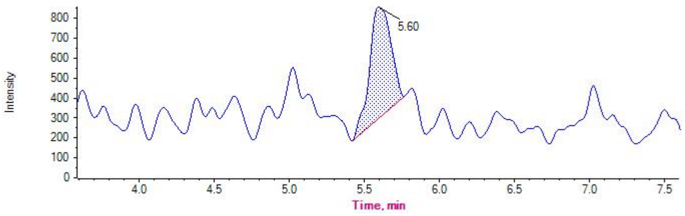

0.2

RT (Exp. RT): 5.60 (5.59) min

Calculated Conc: 0.21 ng/mL

Area: 5.913e3

Sample Type: (Standard)

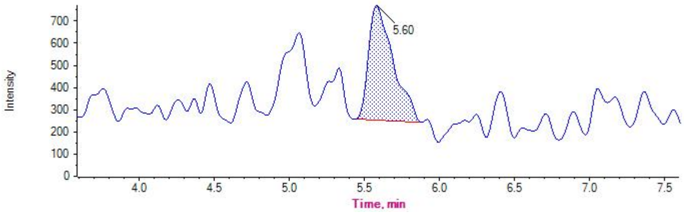

0.5

RT (Exp. RT): 5.62 (5.59) min

Calculated Conc: 0.59 ng/mL

Area: 1.257e4

Sample Type: (Standard)

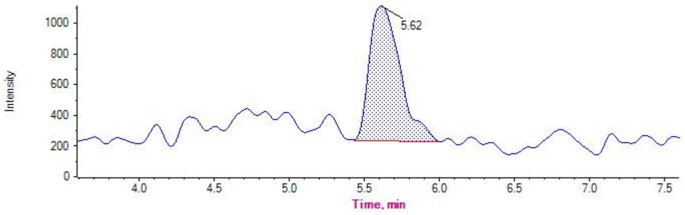

2

RT (Exp. RT): 5.60 (5.59) min

Calculated Conc: 1.82 ng/mL

Area: 3.428e4

Sample Type: (Standard)

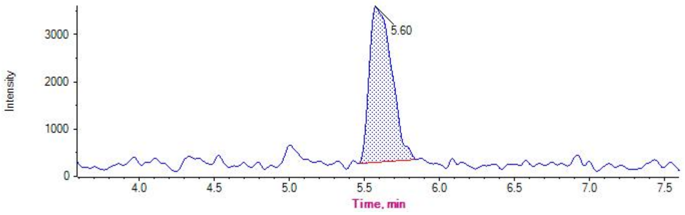

2

RT (Exp. RT): 5.59 (5.59) min

Calculated Conc: 2.04 ng/mL

Area: 3.803e4

Sample Type: (Standard)

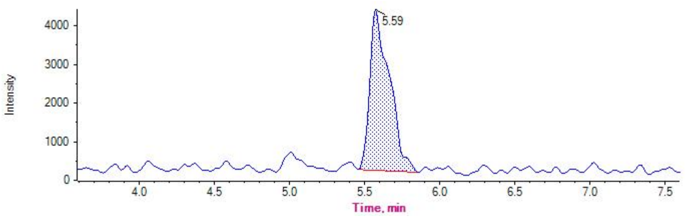

5

RT (Exp. RT): 5.60 (5.59) min

Calculated Conc: 4.58 ng/mL

Area: 8.262e4

Sample Type: (Standard)

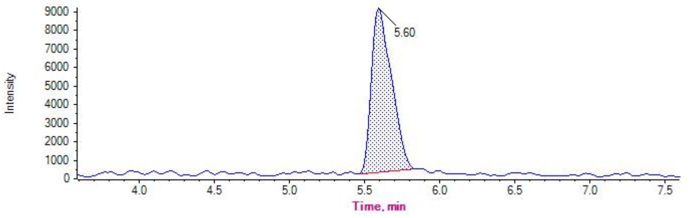

5

RT (Exp. RT): 5.60 (5.59) min

Calculated Conc: 4.88 ng/mL

Area: 8.788e4

Sample Type: (Standard)

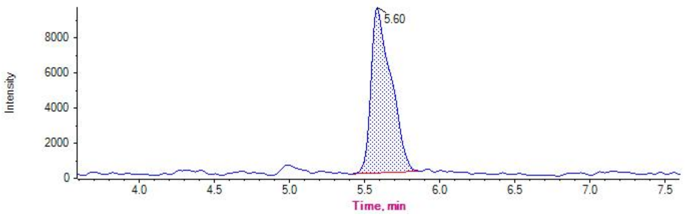

20

RT (Exp. RT): 5.59 (5.59) min

Calculated Conc: 20.78 ng/mL

Area: 3.666e5

Sample Type: (Standard)

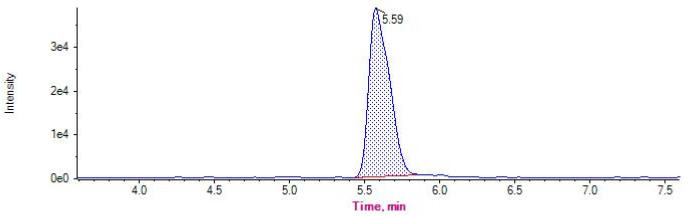

20

RT (Exp. RT): 5.60 (5.59) min

Calculated Conc: 20.34 ng/mL

Area: 3.588e5

Sample Type: (Standard)

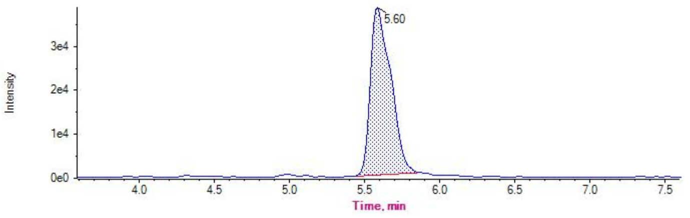

50

RT (Exp. RT): 5.59 (5.59) min

Calculated Conc: 47.04 ng/mL

Area: 8.269e5

Sample Type: (Standard)

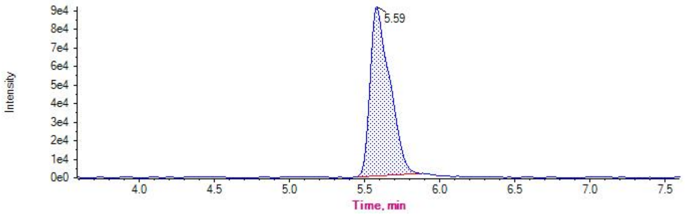

50

RT (Exp. RT): 5.59 (5.59) min

Calculated Conc: 49.14 ng/mL

Area: 8.638e5

Sample Type: (Standard)

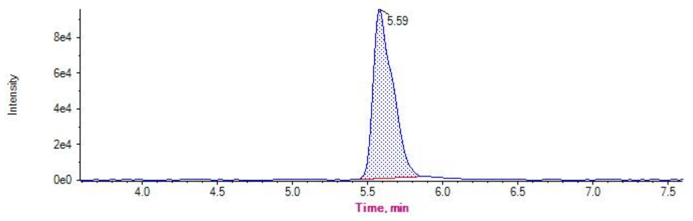

200

RT (Exp. RT): 5.59 (5.59) min

Calculated Conc: 208.50 ng/mL

Area: 3.657e6

Sample Type: (Standard)

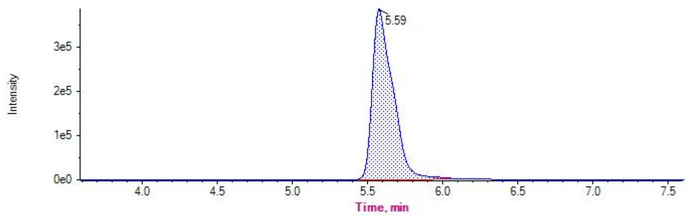

200

RT (Exp. RT): 5.59 (5.59) min

Calculated Conc: 208.08 ng/mL

Area: 3.650e6

Sample Type: (Standard)

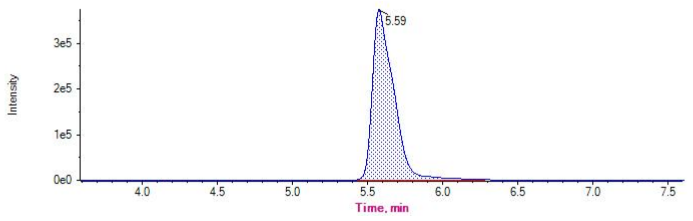

TZR

0.1

RT (Exp. RT): 4.25 (4.23) min

Calculated Conc: 0.09 ng/mL

Area: 2.234e4

Sample Type: (Standard)

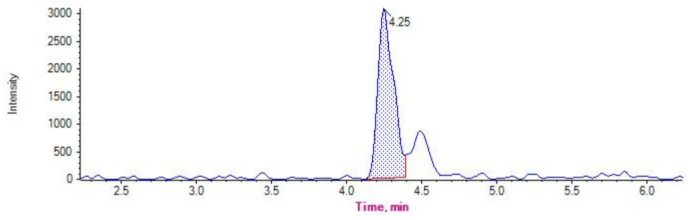

0.1

RT (Exp. RT): 4.25 (4.23) min

Calculated Conc: 0.10 ng/mL

Area: 2.551e4

Sample Type: (Standard)

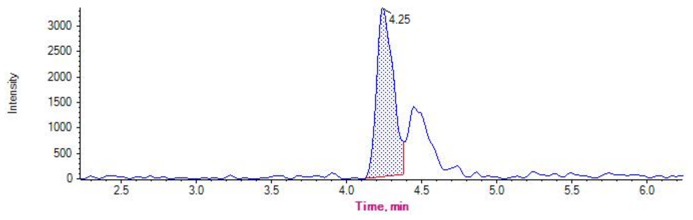

0.2

RT (Exp. RT): 4.24 (4.23) min

Calculated Conc: 0.21 ng/mL

Area: 5.693e4

Sample Type: (Standard)

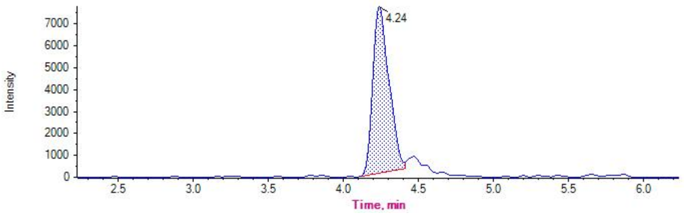

0.2

RT (Exp. RT): 4.25 (4.23) min

Calculated Conc: 0.21 ng/mL

Area: 5.483e4

Sample Type: (Standard)

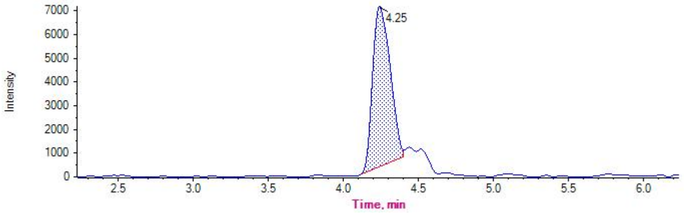

0.5

RT (Exp. RT): 4.24 (4.23) min

Calculated Conc: 0.47 ng/mL

Area: 1.325e5

Sample Type: (Standard)

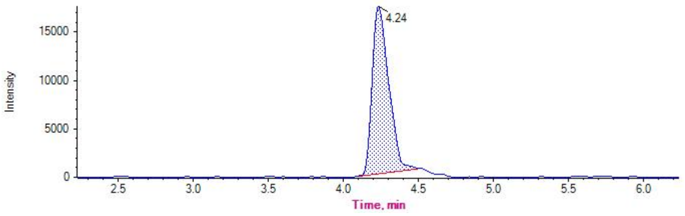

0.5

RT (Exp. RT): 4.24 (4.23) min

Calculated Conc: 0.44 ng/mL

Area: 1.231e5

Sample Type: (Standard)

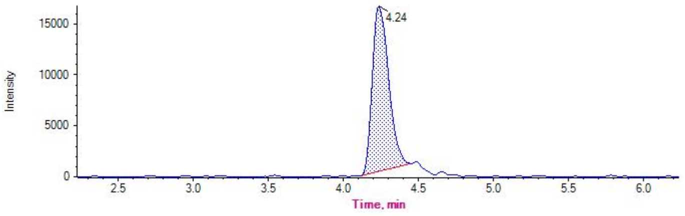

2

RT (Exp. RT): 4.24 (4.23) min

Calculated Conc: 2.03 ng/mL

Area: 5.896e5

Sample Type: (Standard)

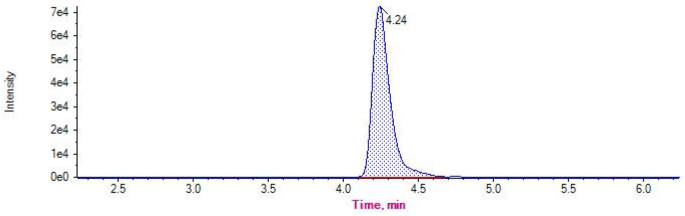

2

RT (Exp. RT): 4.23 (4.23) min

Calculated Conc: 2.08 ng/mL

Area: 6.028e5

Sample Type: (Standard)

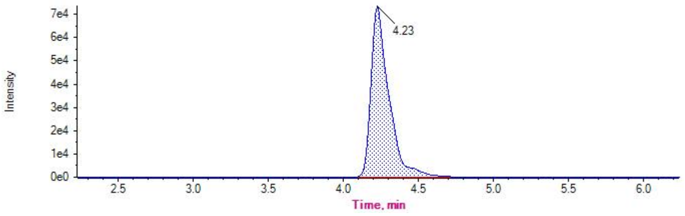

5

RT (Exp. RT):	4.24 (4.23) min
Calculated Conc:	4.77 ng/mL
Area:	1.391e6
Sample Type:	(Standard)

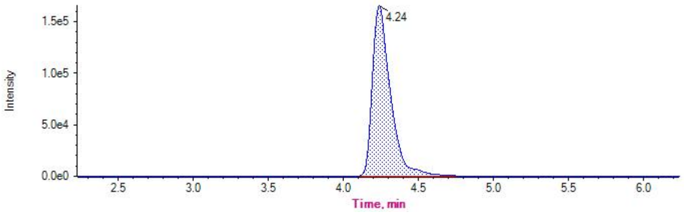

5

RT (Exp. RT):	4.25 (4.23) min
Calculated Conc:	5.10 ng/mL
Area:	1.486e6
Sample Type:	(Standard)

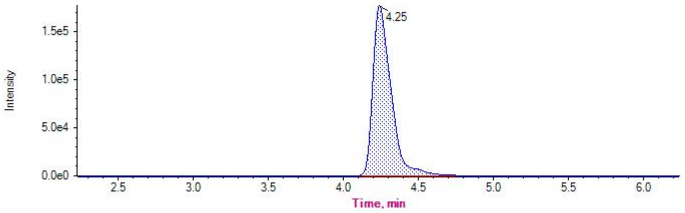

20

RT (Exp. RT):	4.24 (4.23) min
Calculated Conc:	20.70 ng/mL
Area:	6.048e6
Sample Type:	(Standard)

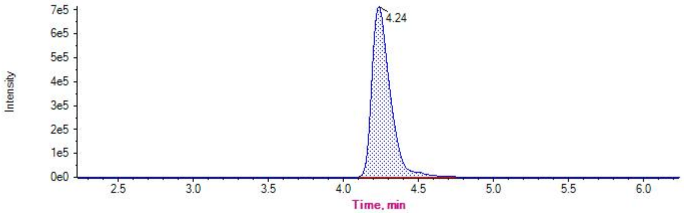

20

RT (Exp. RT):	4.25 (4.23) min
Calculated Conc:	21.08 ng/mL
Area:	6.160e6
Sample Type:	(Standard)

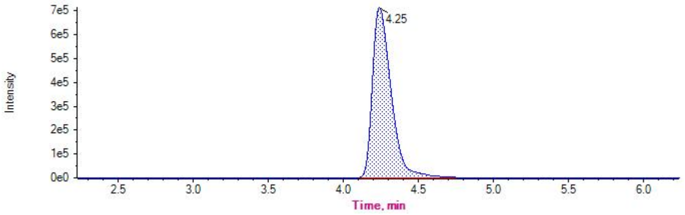

50

RT (Exp. RT):	4.24 (4.23) min
Calculated Conc:	48.06 ng/mL
Area:	1.405e7
Sample Type:	(Standard)

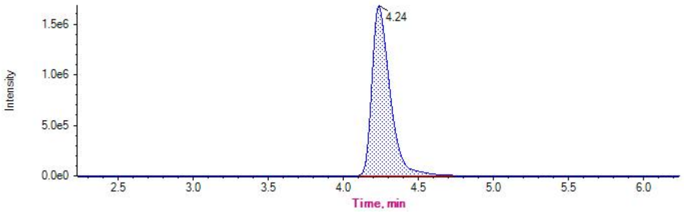

50

RT (Exp. RT):	4.24 (4.23) min
Calculated Conc:	48.66 ng/mL
Area:	1.422e7
Sample Type:	(Standard)

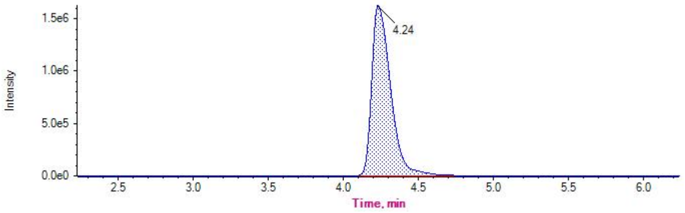

200

RT (Exp. RT): 4.24 (4.23) min

Calculated Conc: 201.53 ng/mL

Area: 5.893e7

Sample Type: (Standard)

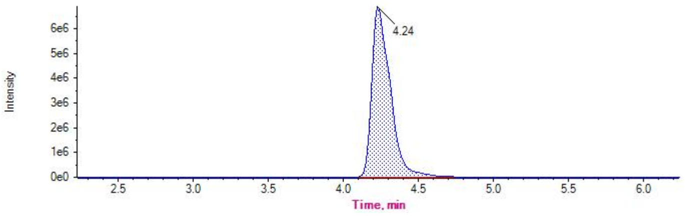

200

RT (Exp. RT): 4.24 (4.23) min

Calculated Conc: 208.49 ng/mL

Area: 6.097e7

Sample Type: (Standard)

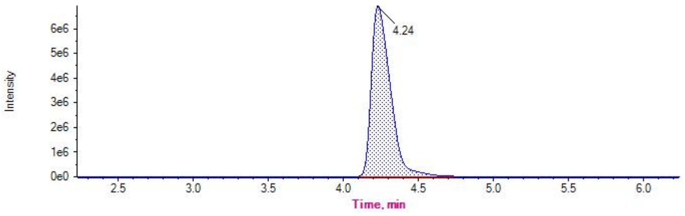

MESA

0.1

RT (Exp. RT): 8.22 (8.18) min

Calculated Conc: 0.10 ng/mL

Area: 5.297e4

Sample Type: (Standard)

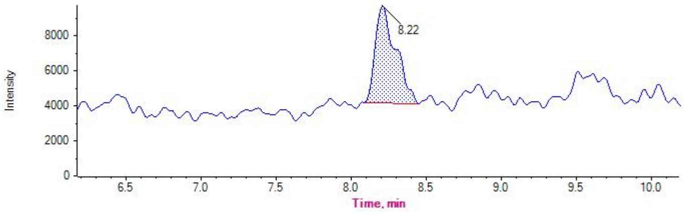

0.1

RT (Exp. RT): 8.20 (8.18) min

Calculated Conc: 0.10 ng/mL

Area: 5.082e4

Sample Type: (Standard)

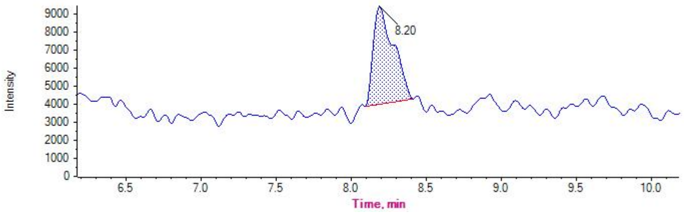

0.2

RT (Exp. RT): 8.20 (8.18) min

Calculated Conc: 0.21 ng/mL

Area: 1.309e5

Sample Type: (Standard)

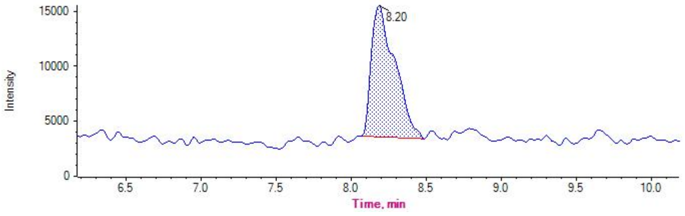

0.2

RT (Exp. RT): 8.19 (8.18) min

Calculated Conc: 0.22 ng/mL

Area: 1.366e5

Sample Type: (Standard)

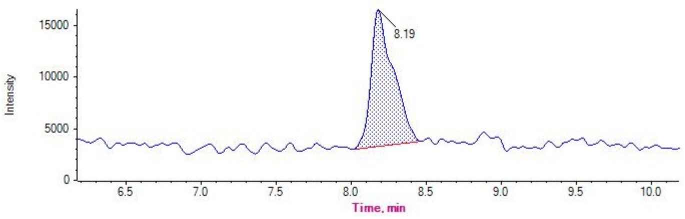

0.5

RT (Exp. RT): 8.20 (8.18) min

Calculated Conc: 0.50 ng/mL

Area: 3.365e5

Sample Type: (Standard)

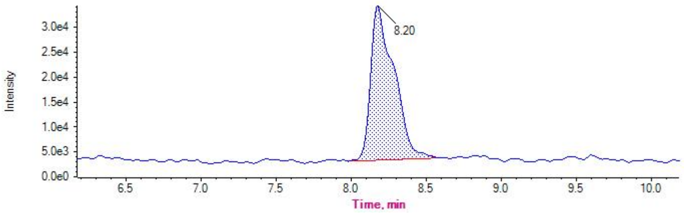

0.5

RT (Exp. RT): 8.20 (8.18) min

Calculated Conc: 0.50 ng/mL

Area: 3.388e5

Sample Type: (Standard)

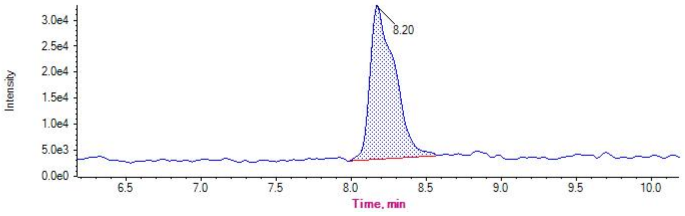

2

RT (Exp. RT): 8.19 (8.18) min

Calculated Conc: 1.93 ng/mL

Area: 1.345e6

Sample Type: (Standard)

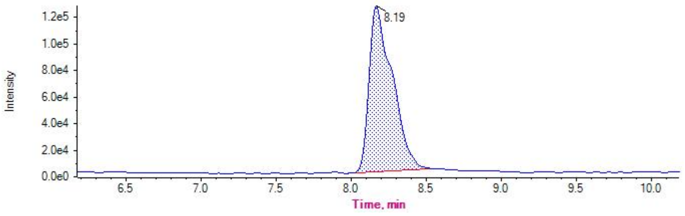

2

RT (Exp. RT): 8.18 (8.18) min

Calculated Conc: 1.99 ng/mL

Area: 1.385e6

Sample Type: (Standard)

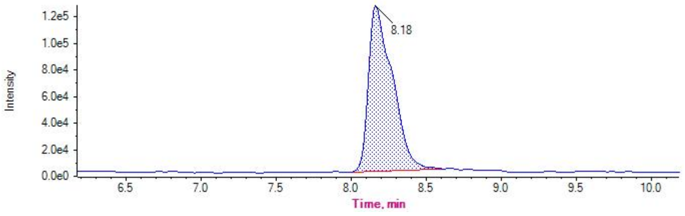

5

RT (Exp. RT): 8.19 (8.18) min

Calculated Conc: 4.75 ng/mL

Area: 3.328e6

Sample Type: (Standard)

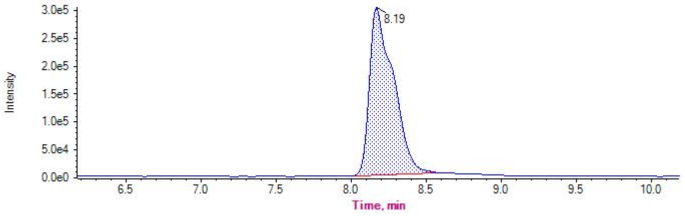

5

RT (Exp. RT):	8.19 (8.18) min
Calculated Conc:	4.78 ng/mL
Area:	3.354e6
Sample Type:	(Standard)

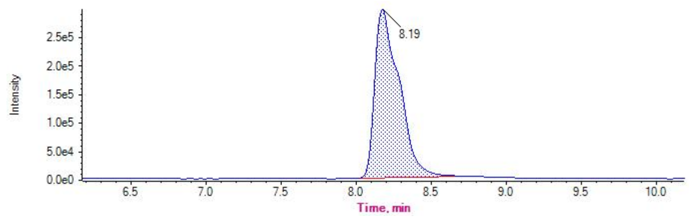

20

RT (Exp. RT):	8.17 (8.18) min
Calculated Conc:	20.49 ng/mL
Area:	1.442e7
Sample Type:	(Standard)

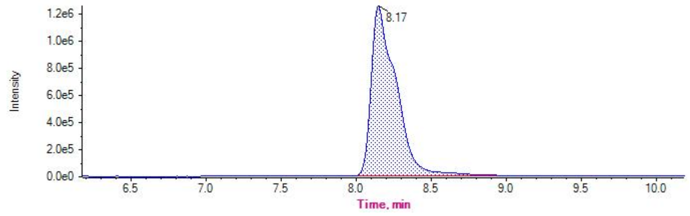

20

RT (Exp. RT):	8.18 (8.18) min
Calculated Conc:	20.47 ng/mL
Area:	1.441e7
Sample Type:	(Standard)

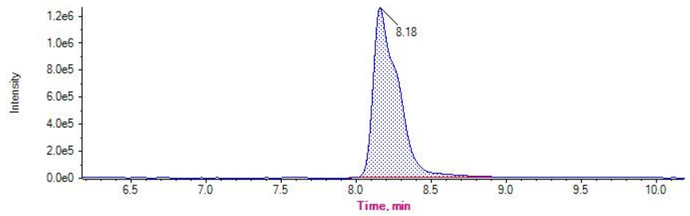

50

RT (Exp. RT):	8.18 (8.18) min
Calculated Conc:	48.64 ng/mL
Area:	3.426e7
Sample Type:	(Standard)

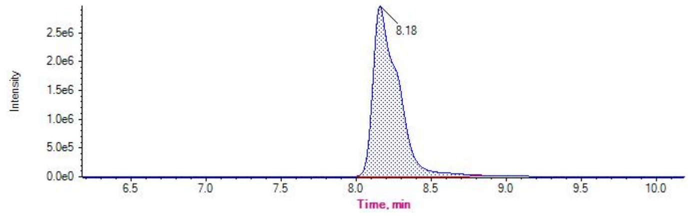

50

RT (Exp. RT):	8.18 (8.18) min
Calculated Conc:	50.34 ng/mL
Area:	3.545e7
Sample Type:	(Standard)

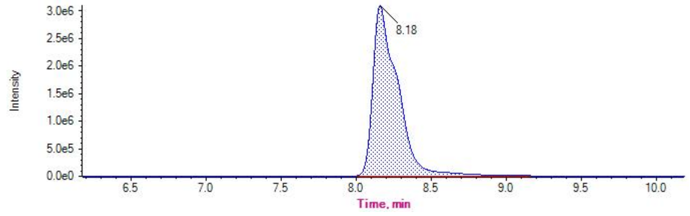

200

RT (Exp. RT):	8.18 (8.18) min
Calculated Conc:	205.16 ng/mL
Area:	1.445e8
Sample Type:	(Standard)

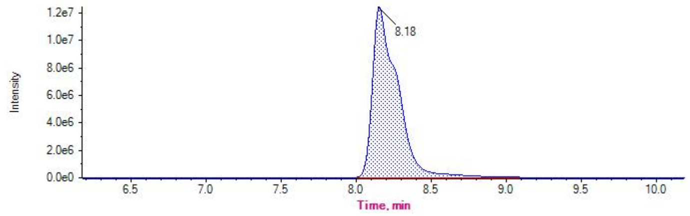

200

RT (Exp. RT): 8.18 (8.18) min

Calculated Conc: 200.69 ng/mL

Area: 1.414e8

Sample Type: (Standard)

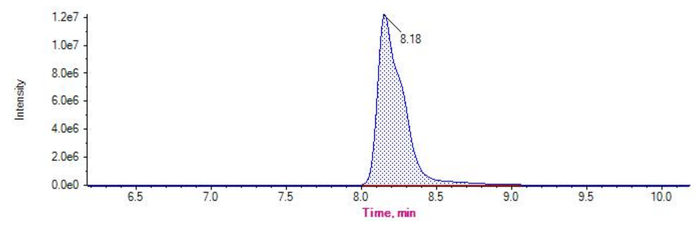

SA

0.2

RT (Exp. RT): 6.14 (6.09) min

Calculated Conc: 0.19 ng/mL

Area: 1.091e5

Sample Type: (Standard)

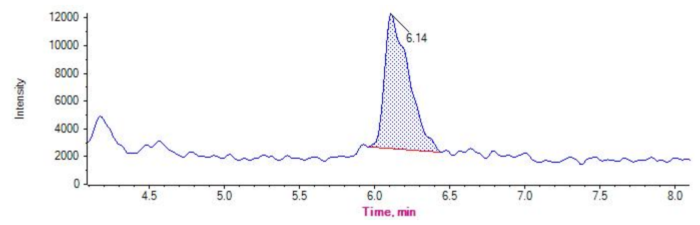

0.5

RT (Exp. RT): 6.13 (6.09) min

Calculated Conc: 0.55 ng/mL

Area: 1.169e5

Sample Type: (Standard)

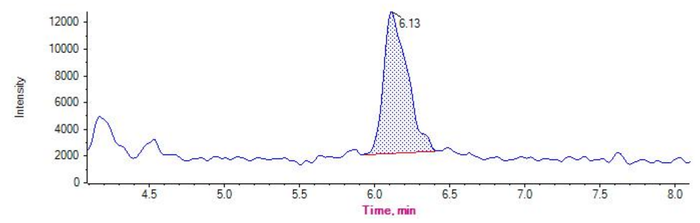

2

RT (Exp. RT): 6.13 (6.09) min

Calculated Conc: 1.76 ng/mL

Area: 1.437e5

Sample Type: (Standard)

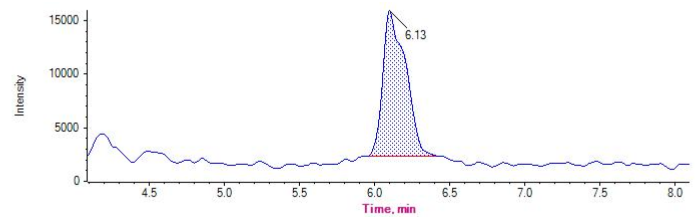

2

RT (Exp. RT): 6.11 (6.09) min

Calculated Conc: 2.36 ng/mL

Area: 1.568e5

Sample Type: (Standard)

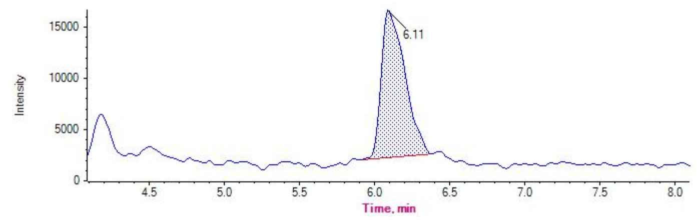

5

RT (Exp. RT): 6.12 (6.09) min

Calculated Conc: 4.58 ng/mL

Area: 2.057e5

Sample Type: (Standard)

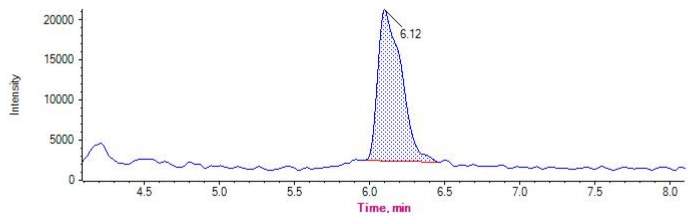

20

RT (Exp. RT): 6.10 (6.09) min

Calculated Conc: 19.79 ng/mL

Area: 5.407e5

Sample Type: (Standard)

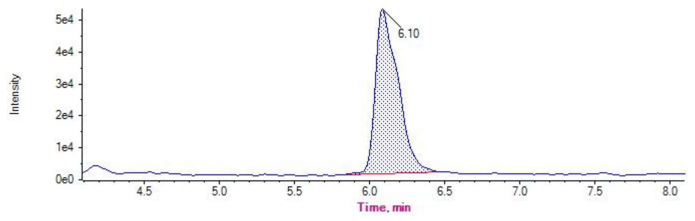

20

RT (Exp. RT): 6.12 (6.09) min

Calculated Conc: 22.99 ng/mL

Area: 6.112e5

Sample Type: (Standard)

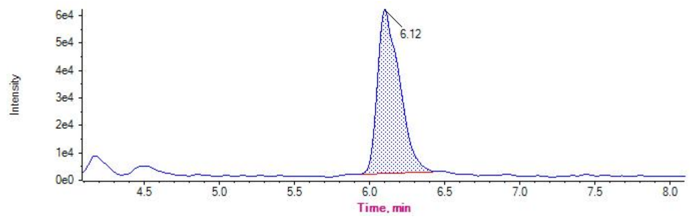

50

RT (Exp. RT): 6.11 (6.09) min

Calculated Conc: 47.14 ng/mL

Area: 1.143e6

Sample Type: (Standard)

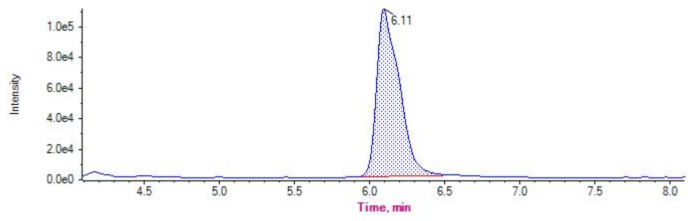

50

RT (Exp. RT): 6.11 (6.09) min

Calculated Conc: 47.37 ng/mL

Area: 1.148e6

Sample Type: (Standard)

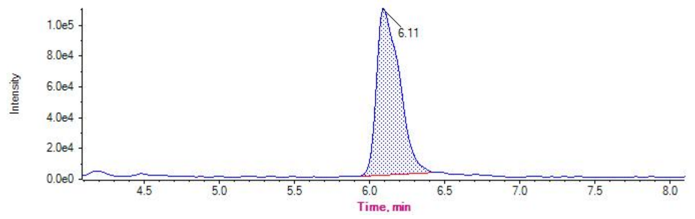

200

RT (Exp. RT): 6.11 (6.09) min

Calculated Conc: 196.07 ng/mL

Area: 4.424e6

Sample Type: (Standard)

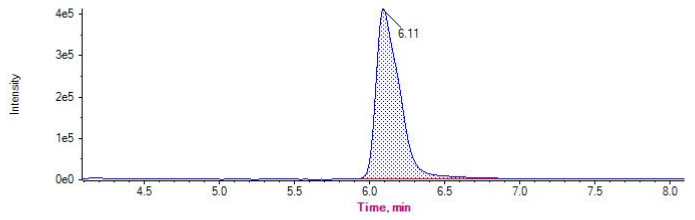

200

RT (Exp. RT): 6.11 (6.09) min

Calculated Conc: 192.63 ng/mL

Area: 4.348e6

Sample Type: (Standard)

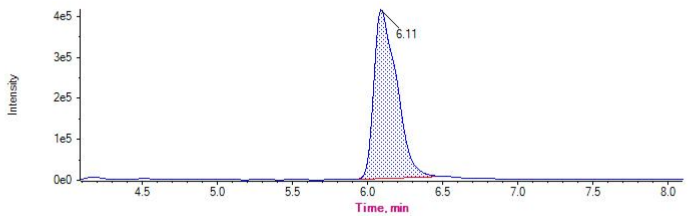

JA

0.1

RT (Exp. RT): 7.64 (7.58) min

Calculated Conc: 0.10 ng/mL

Area: 6.351e2

Sample Type: (Standard)

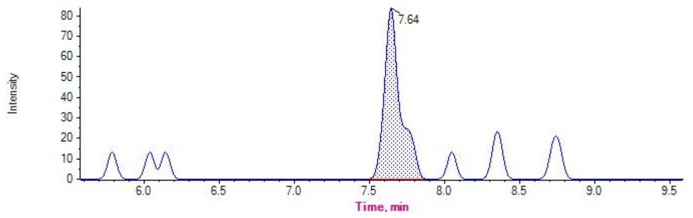

0.2

RT (Exp. RT): 7.63 (7.58) min

Calculated Conc: 0.20 ng/mL

Area: 8.083e2

Sample Type: (Standard)

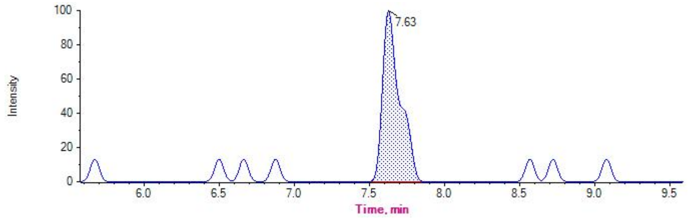

0.5

RT (Exp. RT): 7.60 (7.58) min

Calculated Conc: 0.54 ng/mL

Area: 1.426e3

Sample Type: (Standard)

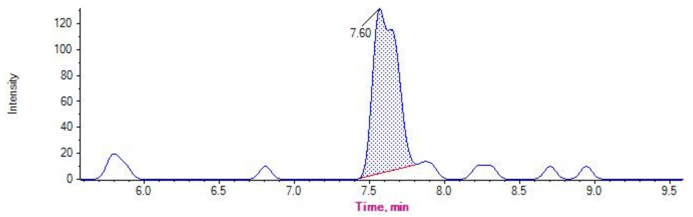

2

RT (Exp. RT): 7.62 (7.58) min

Calculated Conc: 1.75 ng/mL

Area: 3.581e3

Sample Type: (Standard)

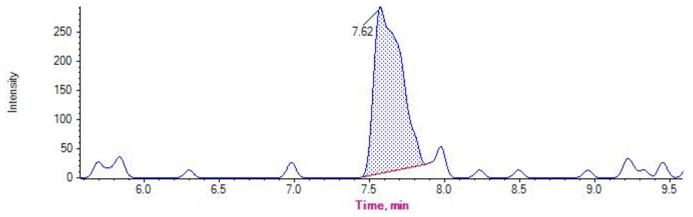

2

RT (Exp. RT): 7.60 (7.58) min

Calculated Conc: 1.68 ng/mL

Area: 3.452e3

Sample Type: (Standard)

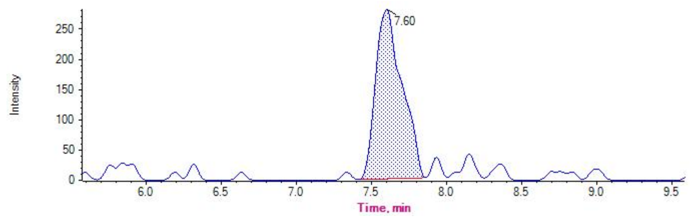

5

RT (Exp. RT): 7.63 (7.58) min

Calculated Conc: 4.32 ng/mL

Area: 8.159e3

Sample Type: (Standard)

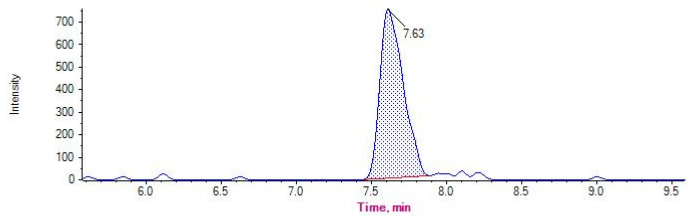

5

RT (Exp. RT): 7.62 (7.58) min

Calculated Conc: 4.16 ng/mL

Area: 7.870e3

Sample Type: (Standard)

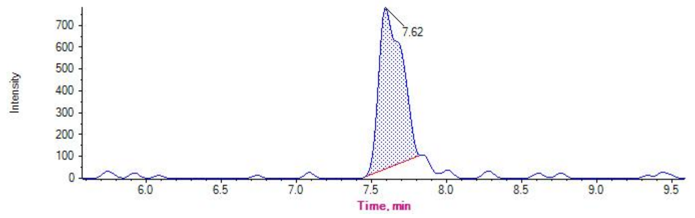

20

RT (Exp. RT): 7.60 (7.58) min

Calculated Conc: 21.32 ng/mL

Area: 3.845e4

Sample Type: (Standard)

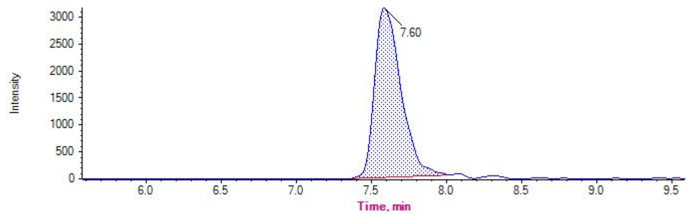

20

RT (Exp. RT): 7.62 (7.58) min

Calculated Conc: 23.54 ng/mL

Area: 4.241e4

Sample Type: (Standard)

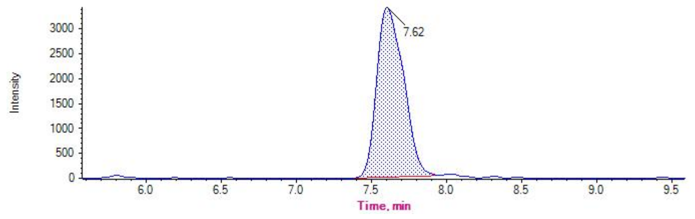

50

RT (Exp. RT): 7.61 (7.58) min

Calculated Conc: 50.09 ng/mL

Area: 8.974e4

Sample Type: (Standard)

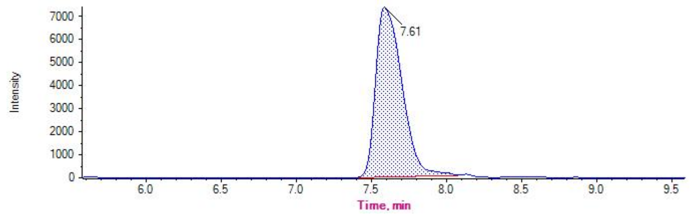

50

RT (Exp. RT): 7.61 (7.58) min

Calculated Conc: 52.76 ng/mL

Area: 9.449e4

Sample Type: (Standard)

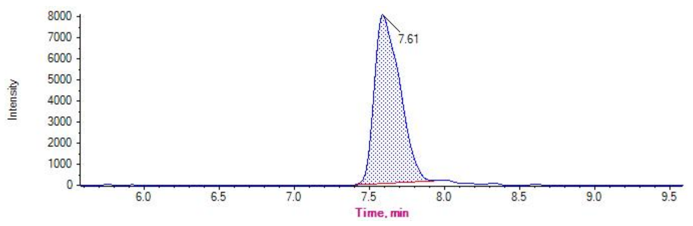

200

RT (Exp. RT): 7.60 (7.58) min

Calculated Conc: 218.01 ng/mL

Area: 3.890e5

Sample Type: (Standard)

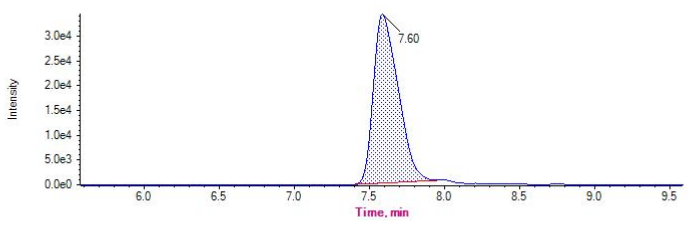

200

RT (Exp. RT): 7.60 (7.58) min

Calculated Conc: 222.53 ng/mL

Area: 3.971e5

Sample Type: (Standard)

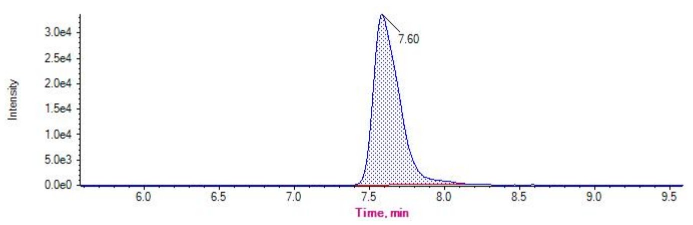

MEJA

0.1

RT (Exp. RT): 9.07 (9.03) min

Calculated Conc: 0.11 ng/mL

Area: 1.704e4

Sample Type: (Standard)

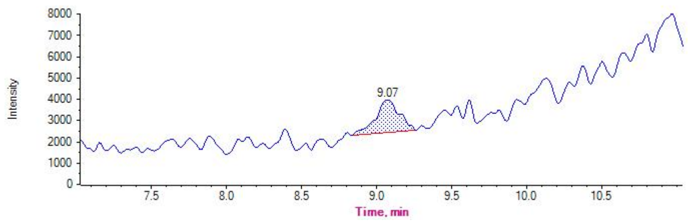

0.2

RT (Exp. RT): 9.08 (9.03) min

Calculated Conc: 0.21 ng/mL

Area: 2.994e4

Sample Type: (Standard)

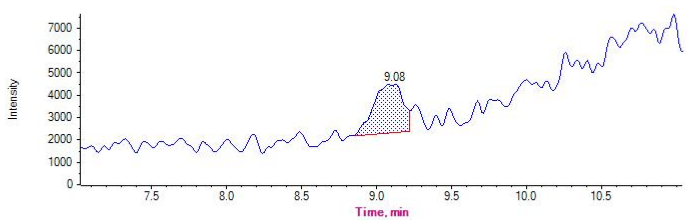

0.2

RT (Exp. RT): 9.07 (9.03) min

Calculated Conc: 0.22 ng/mL

Area: 3.238e4

Sample Type: (Standard)

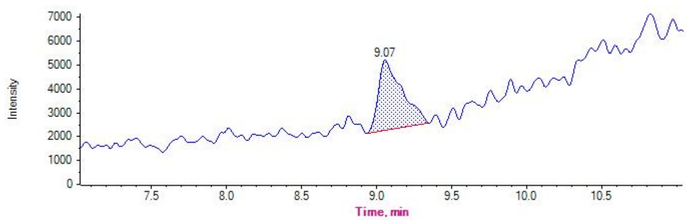

0.5

RT (Exp. RT): 9.07 (9.03) min

Calculated Conc: 0.42 ng/mL

Area: 6.013e4

Sample Type: (Standard)

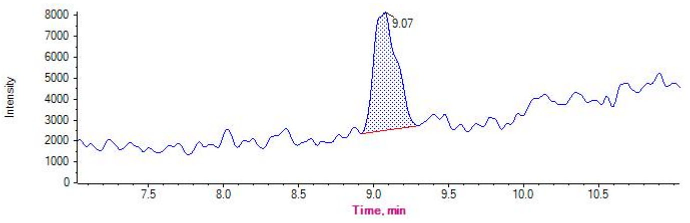

2

RT (Exp. RT): 9.05 (9.03) min

Calculated Conc: 2.01 ng/mL

Area: 2.811e5

Sample Type: (Standard)

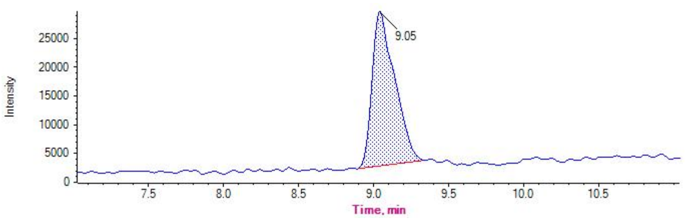

2

RT (Exp. RT): 9.05 (9.03) min

Calculated Conc: 1.93 ng/mL

Area: 2.706e5

Sample Type: (Standard)

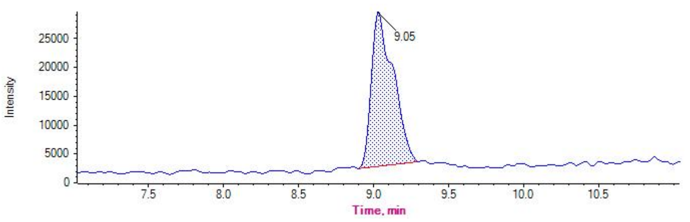

5

RT (Exp. RT): 9.05 (9.03) min

Calculated Conc: 4.75 ng/mL

Area: 6.631e5

Sample Type: (Standard)

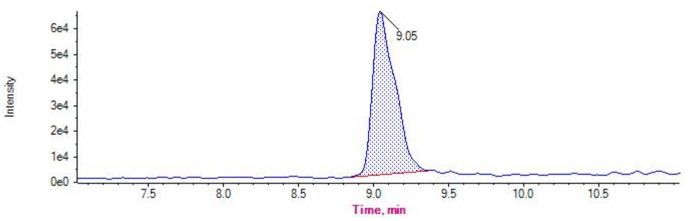

5

RT (Exp. RT): 9.06 (9.03) min

Calculated Conc: 4.46 ng/mL

Area: 6.231e5

Sample Type: (Standard)

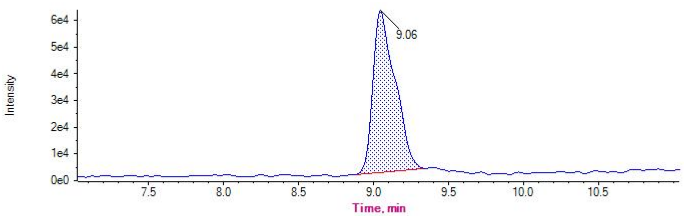

20

RT (Exp. RT): 9.04 (9.03) min

Calculated Conc: 20.97 ng/mL

Area: 2.925e6

Sample Type: (Standard)

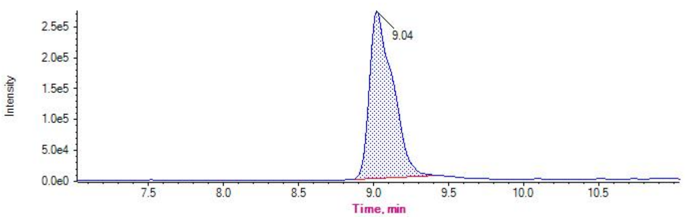

20

RT (Exp. RT): 9.05 (9.03) min

Calculated Conc: 21.43 ng/mL

Area: 2.989e6

Sample Type: (Standard)

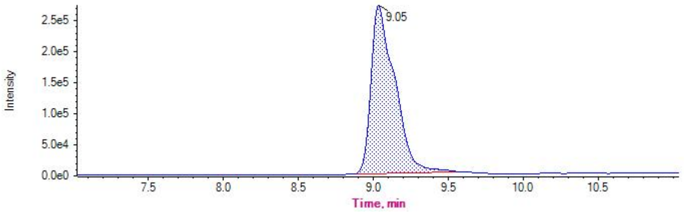

50

RT (Exp. RT): 9.05 (9.03) min

Calculated Conc: 51.89 ng/mL

Area: 7.238e6

Sample Type: (Standard)

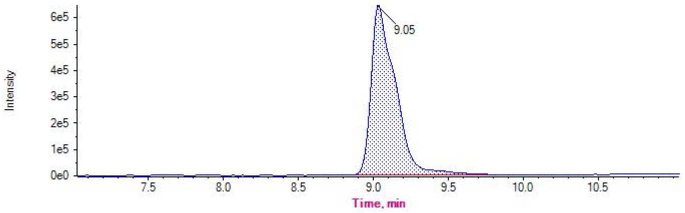

50

RT (Exp. RT): 9.05 (9.03) min

Calculated Conc: 52.55 ng/mL

Area: 7.330e6

Sample Type: (Standard)

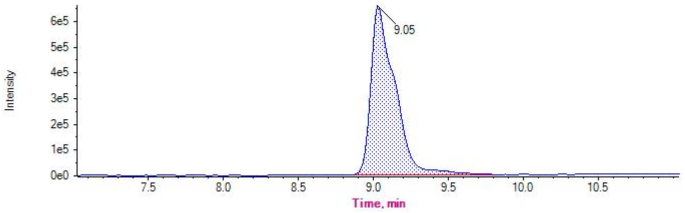

200

RT (Exp. RT): 9.05 (9.03) min

Calculated Conc: 221.21 ng/mL

Area: 3.085e7

Sample Type: (Standard)

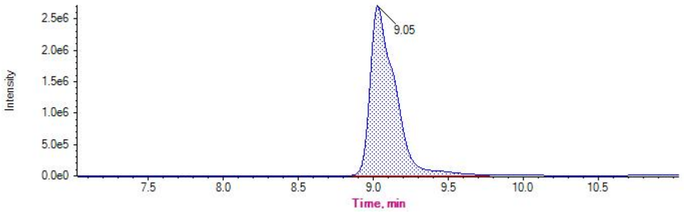

200

RT (Exp. RT): 9.04 (9.03) min

Calculated Conc: 219.22 ng/mL

Area: 3.057e7

Sample Type: (Standard)

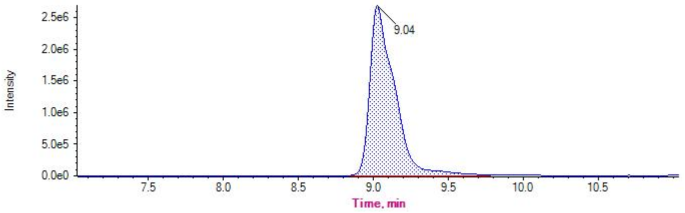

IBA

0.2

RT (Exp. RT): 7.23 (7.21) min

Calculated Conc: 0.20 ng/mL

Area: 1.314e2

Sample Type: (Standard)

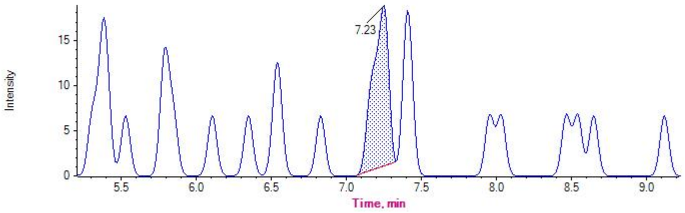

2

RT (Exp. RT): 7.23 (7.21) min

Calculated Conc: 1.97 ng/mL

Area: 1.324e3

Sample Type: (Standard)

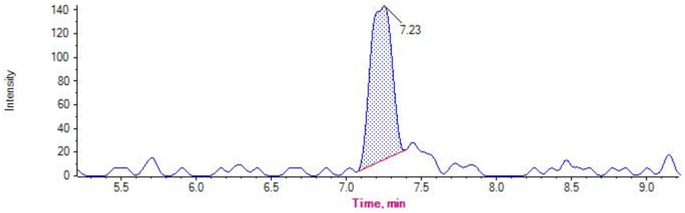

2

RT (Exp. RT): 7.22 (7.21) min

Calculated Conc: 2.03 ng/mL

Area: 1.364e3

Sample Type: (Standard)

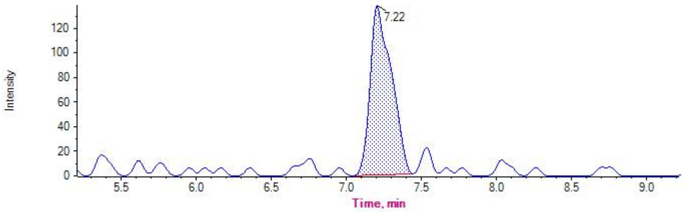

5

RT (Exp. RT): 7.22 (7.21) min

Calculated Conc: 5.26 ng/mL

Area: 3.542e3

Sample Type: (Standard)

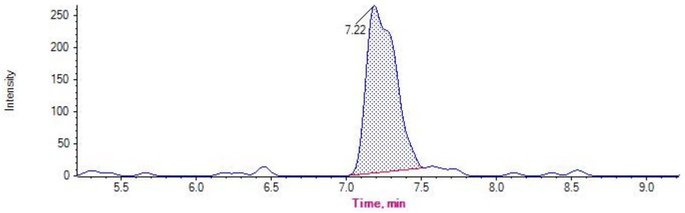

5

RT (Exp. RT): 7.22 (7.21) min

Calculated Conc: 4.80 ng/mL

Area: 3.234e3

Sample Type: (Standard)

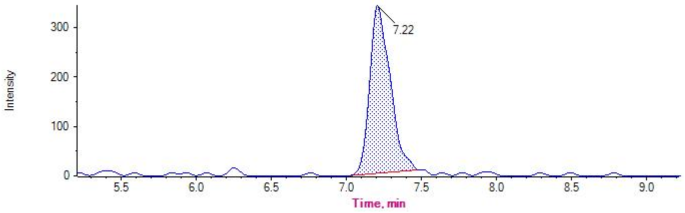

20

RT (Exp. RT): 7.19 (7.21) min

Calculated Conc: 20.56 ng/mL

Area: 1.386e4

Sample Type: (Standard)

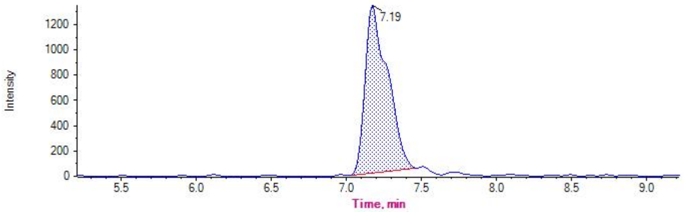

20

RT (Exp. RT): 7.20 (7.21) min

Calculated Conc: 22.39 ng/mL

Area: 1.509e4

Sample Type: (Standard)

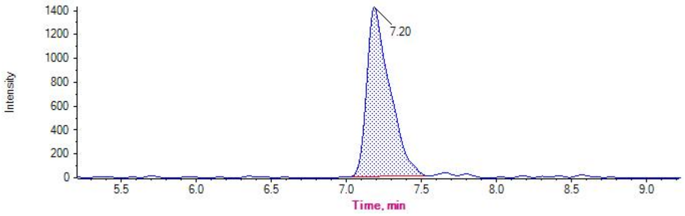

50

RT (Exp. RT): 7.20 (7.21) min

Calculated Conc: 47.48 ng/mL

Area: 3.201e4

Sample Type: (Standard)

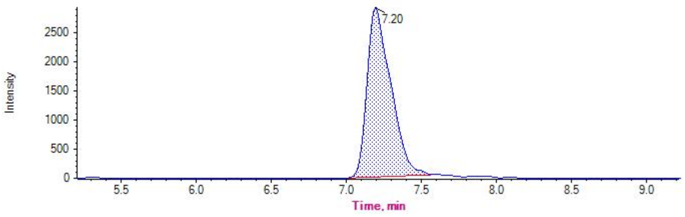

50

RT (Exp. RT): 7.21 (7.21) min

Calculated Conc: 47.11 ng/mL

Area: 3.176e4

Sample Type: (Standard)

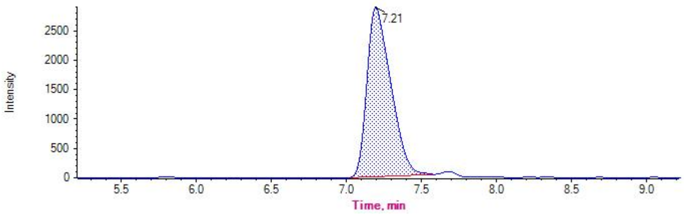

200

RT (Exp. RT): 7.20 (7.21) min

Calculated Conc: 195.21 ng/mL

Area: 1.316e5

Sample Type: (Standard)

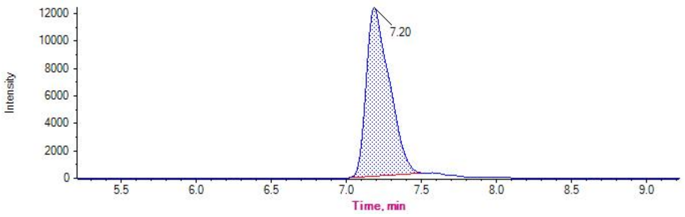

200

RT (Exp. RT): 7.20 (7.21) min

Calculated Conc: 195.28 ng/mL

Area: 1.317e5

Sample Type: (Standard)

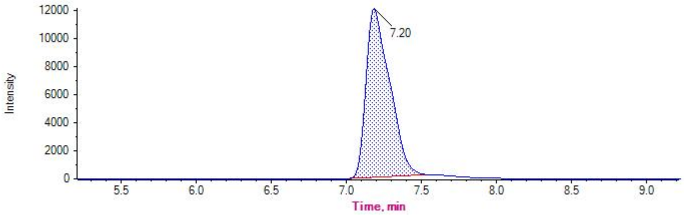

IP

0.1

RT (Exp. RT): 5.66 (5.61) min

Calculated Conc: 0.08 ng/mL

Area: 8.942e4

Sample Type: (Standard)

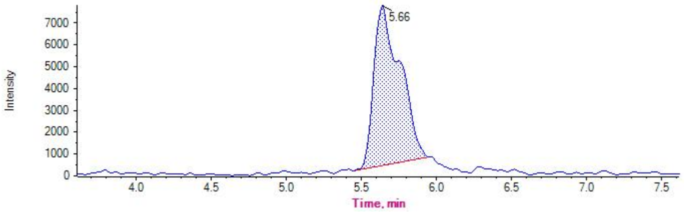

0.1

RT (Exp. RT): 5.67 (5.61) min

Calculated Conc: 0.11 ng/mL

Area: 1.146e5

Sample Type: (Standard)

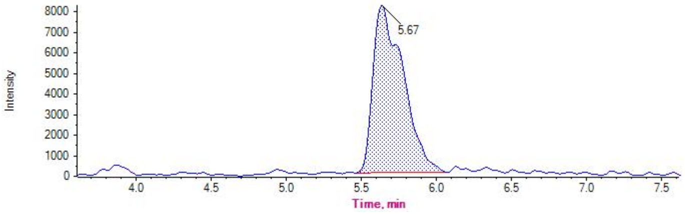

0.2

RT (Exp. RT): 5.65 (5.61) min

Calculated Conc: 0.21 ng/mL

Area: 1.918e5

Sample Type: (Standard)

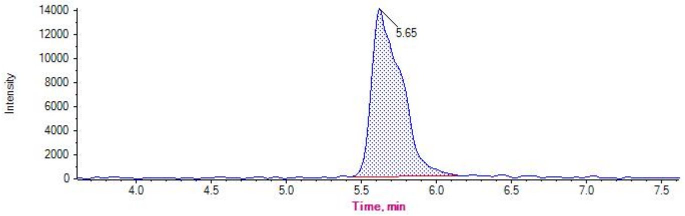

0.2

RT (Exp. RT): 5.65 (5.61) min

Calculated Conc: 0.21 ng/mL

Area: 1.899e5

Sample Type: (Standard)

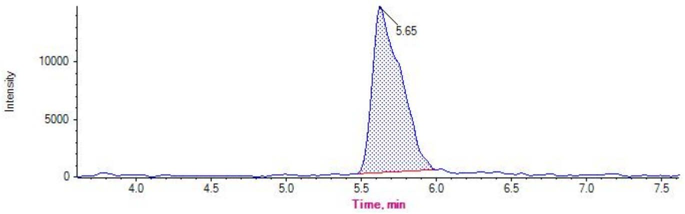

0.5

RT (Exp. RT): 5.65 (5.61) min

Calculated Conc: 0.51 ng/mL

Area: 4.295e5

Sample Type: (Standard)

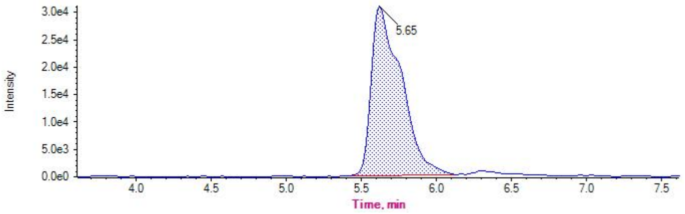

0.5

RT (Exp. RT): 5.65 (5.61) min

Calculated Conc: 0.50 ng/mL

Area: 4.233e5

Sample Type: (Standard)

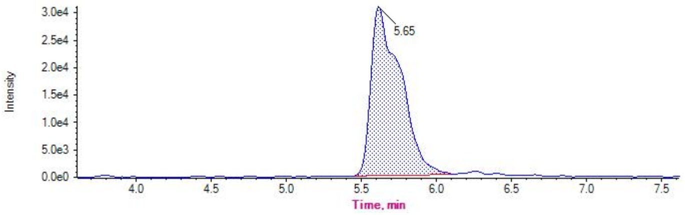

2

RT (Exp. RT): 5.64 (5.61) min

Calculated Conc: 2.03 ng/mL

Area: 1.655e6

Sample Type: (Standard)

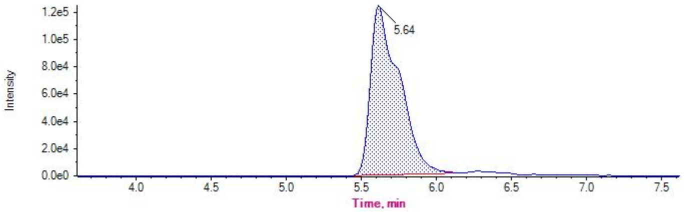

2

RT (Exp. RT): 5.63 (5.61) min

Calculated Conc: 2.03 ng/mL

Area: 1.657e6

Sample Type: (Standard)

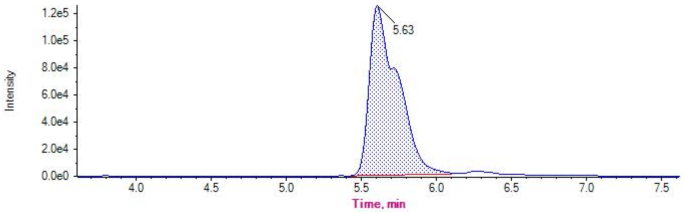

5

RT (Exp. RT): 5.64 (5.61) min

Calculated Conc: 5.03 ng/mL

Area: 4.063e6

Sample Type: (Standard)

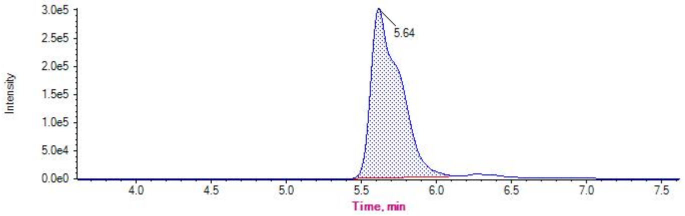

5

RT (Exp. RT): 5.65 (5.61) min

Calculated Conc: 4.94 ng/mL

Area: 3.993e6

Sample Type: (Standard)

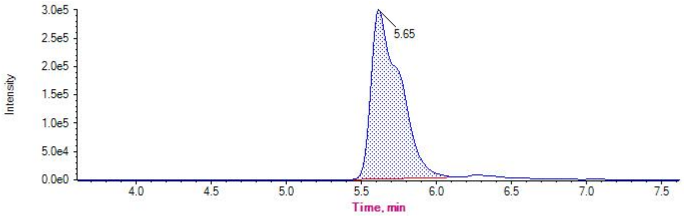

20

RT (Exp. RT): 5.63 (5.61) min

Calculated Conc: 21.38 ng/mL

Area: 1.719e7

Sample Type: (Standard)

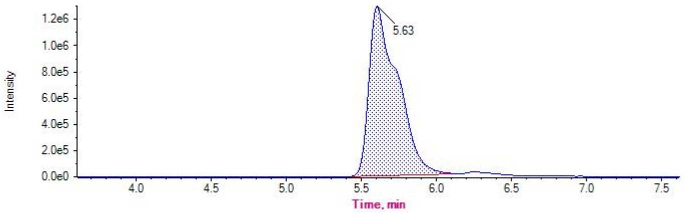

20

RT (Exp. RT): 5.64 (5.61) min

Calculated Conc: 20.62 ng/mL

Area: 1.658e7

Sample Type: (Standard)

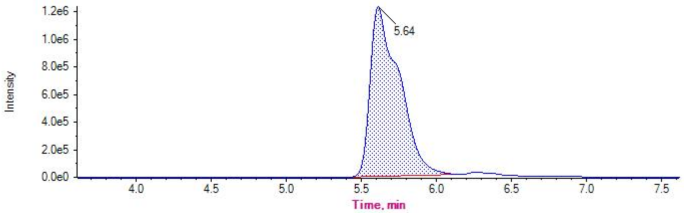

50		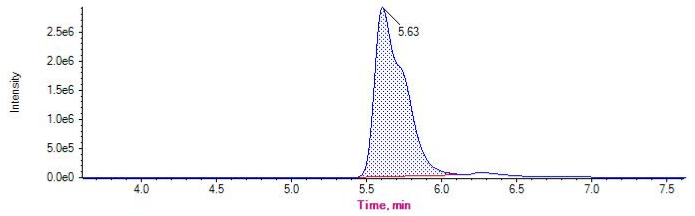
RT (Exp. RT):	5.63 (5.61) min	
Calculated Conc:	48.96 ng/mL	
Area:	3.935e7	
Sample Type:	(Standard)	

50		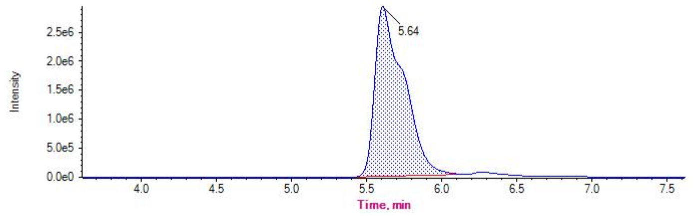
RT (Exp. RT):	5.64 (5.61) min	
Calculated Conc:	49.03 ng/mL	
Area:	3.941e7	
Sample Type:	(Standard)	

200		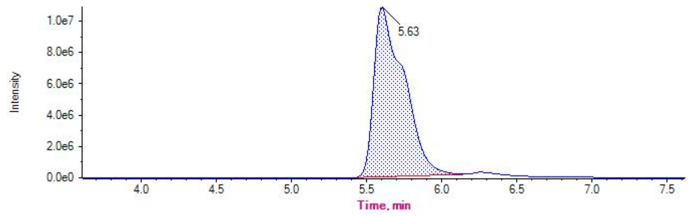
RT (Exp. RT):	5.63 (5.61) min	
Calculated Conc:	187.74 ng/mL	
Area:	1.508e8	
Sample Type:	(Standard)	

200		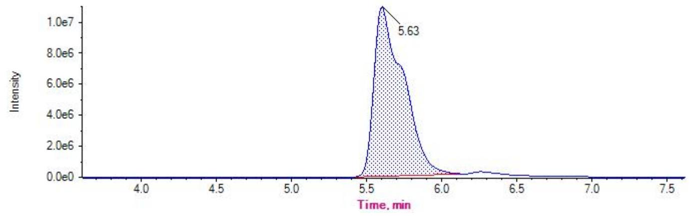
RT (Exp. RT):	5.63 (5.61) min	
Calculated Conc:	185.91 ng/mL	
Area:	1.494e8	
Sample Type:	(Standard)	

IPA

0.1		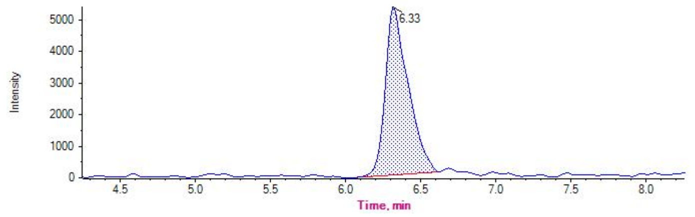
RT (Exp. RT):	6.33 (6.25) min	
Calculated Conc:	0.09 ng/mL	
Area:	5.634e4	
Sample Type:	(Standard)	

0.1

RT (Exp. RT): 6.32 (6.25) min

Calculated Conc: 0.10 ng/mL

Area: 6.244e4

Sample Type: (Standard)

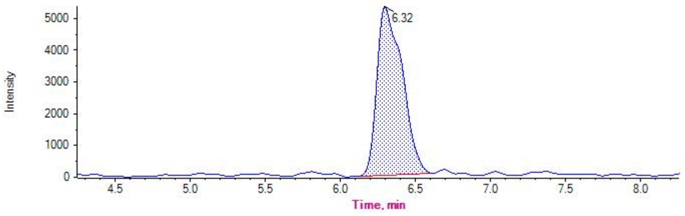

0.2

RT (Exp. RT): 6.30 (6.25) min

Calculated Conc: 0.21 ng/mL

Area: 1.235e5

Sample Type: (Standard)

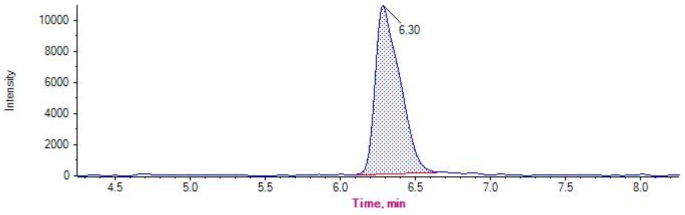

0.2

RT (Exp. RT): 6.30 (6.25) min

Calculated Conc: 0.23 ng/mL

Area: 1.321e5

Sample Type: (Standard)

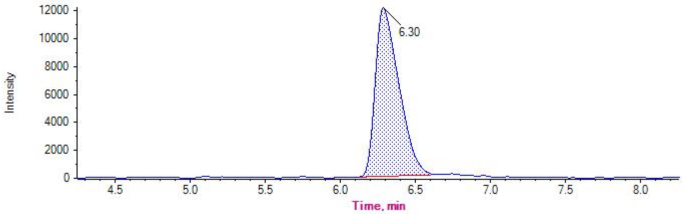

0.5

RT (Exp. RT): 6.30 (6.25) min

Calculated Conc: 0.46 ng/mL

Area: 2.675e5

Sample Type: (Standard)

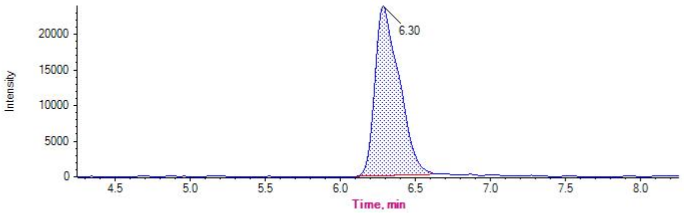

0.5

RT (Exp. RT): 6.30 (6.25) min

Calculated Conc: 0.45 ng/mL

Area: 2.623e5

Sample Type: (Standard)

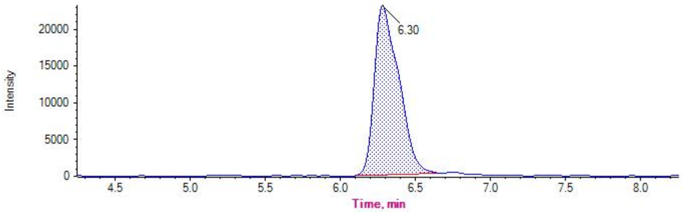

2

RT (Exp. RT): 6.29 (6.25) min

Calculated Conc: 1.91 ng/mL

Area: 1.094e6

Sample Type: (Standard)

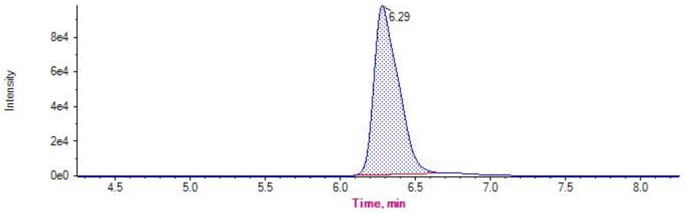

2

RT (Exp. RT): 6.29 (6.25) min

Calculated Conc: 2.04 ng/mL

Area: 1.166e6

Sample Type: (Standard)

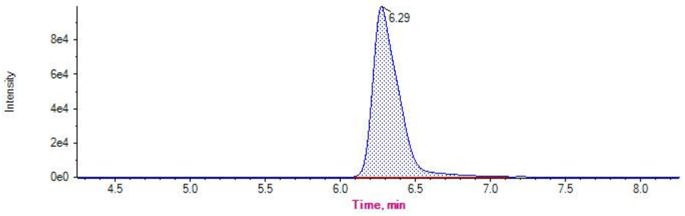

5

RT (Exp. RT): 6.29 (6.25) min

Calculated Conc: 4.67 ng/mL

Area: 2.661e6

Sample Type: (Standard)

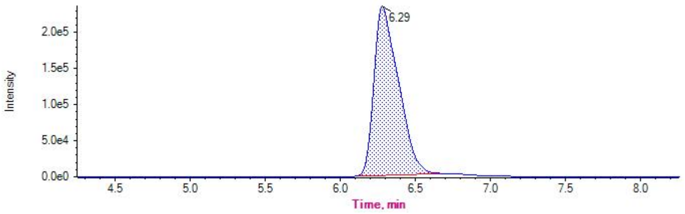

5

RT (Exp. RT): 6.29 (6.25) min

Calculated Conc: 5.02 ng/mL

Area: 2.862e6

Sample Type: (Standard)

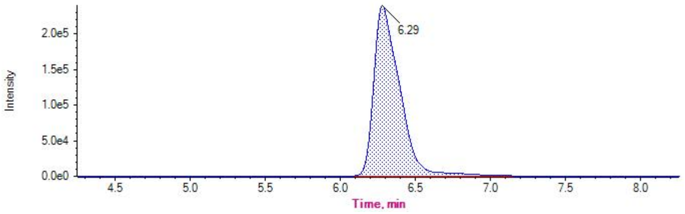

20

RT (Exp. RT): 6.27 (6.25) min

Calculated Conc: 21.53 ng/mL

Area: 1.227e7

Sample Type: (Standard)

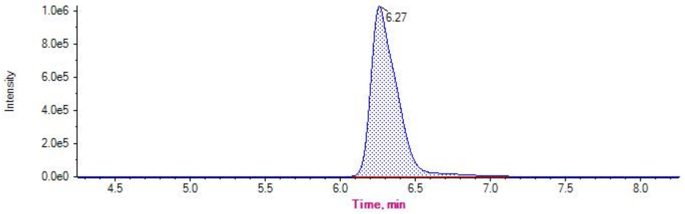

20

RT (Exp. RT): 6.29 (6.25) min

Calculated Conc: 21.22 ng/mL

Area: 1.209e7

Sample Type: (Standard)

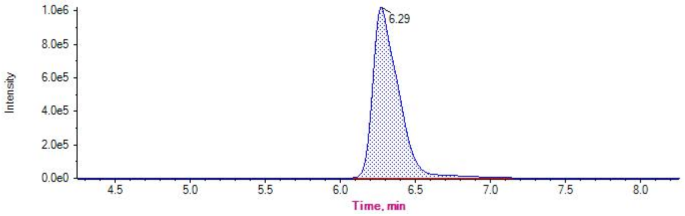

50

RT (Exp. RT): 6.28 (6.25) min

Calculated Conc: 49.86 ng/mL

Area: 2.840e7

Sample Type: (Standard)

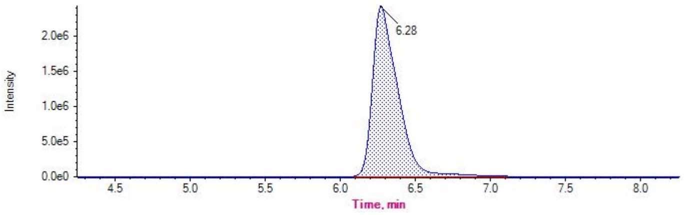

50

RT (Exp. RT): 6.29 (6.25) min

Calculated Conc: 49.84 ng/mL

Area: 2.839e7

Sample Type: (Standard)

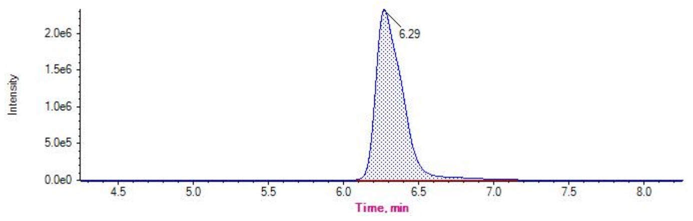

200

RT (Exp. RT): 6.28 (6.25) min

Calculated Conc: 200.63 ng/mL

Area: 1.143e8

Sample Type: (Standard)

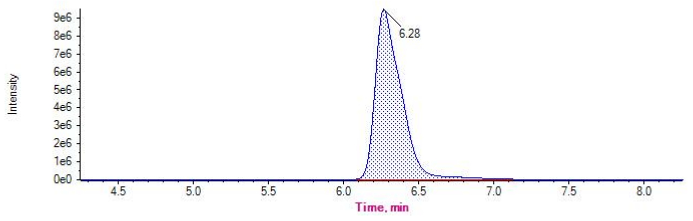

200

RT (Exp. RT): 6.28 (6.25) min

Calculated Conc: 199.15 ng/mL

Area: 1.134e8

Sample Type: (Standard)

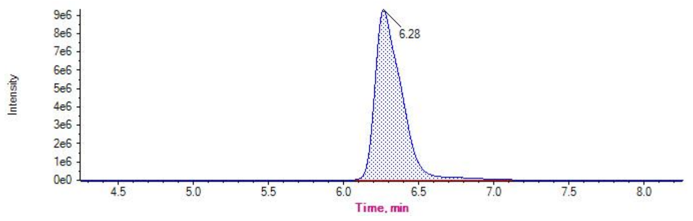

ZEATIN

0.1

RT (Exp. RT): 3.42 (3.39) min

Calculated Conc: 0.12 ng/mL

Area: 2.530e3

Sample Type: (Standard)

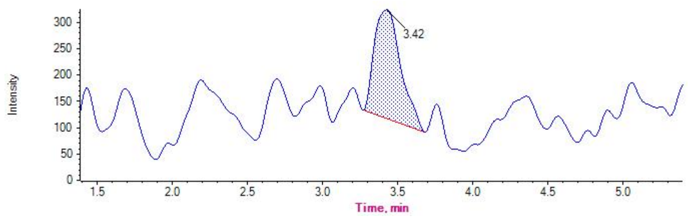

0.1

RT (Exp. RT): 3.39 (3.39) min

Calculated Conc: 0.08 ng/mL

Area: 1.573e3

Sample Type: (Standard)

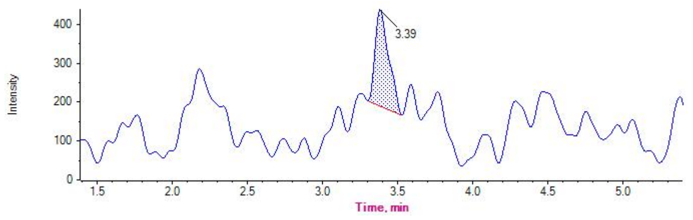

0.2

RT (Exp. RT): 3.37 (3.39) min

Calculated Conc: 0.20 ng/mL

Area: 4.574e3

Sample Type: (Standard)

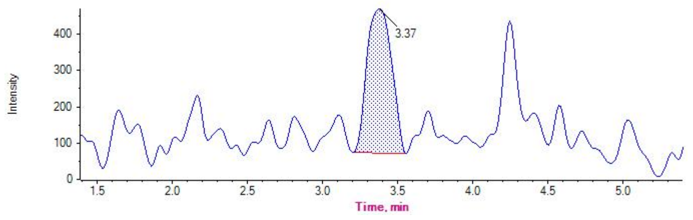

0.2

RT (Exp. RT): 3.38 (3.39) min

Calculated Conc: 0.24 ng/mL

Area: 5.681e3

Sample Type: (Standard)

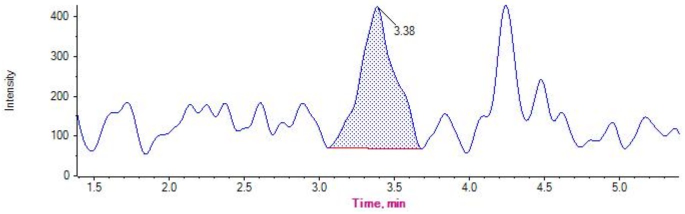

0.5

RT (Exp. RT): 3.38 (3.39) min

Calculated Conc: 0.47 ng/mL

Area: 1.123e4

Sample Type: (Standard)

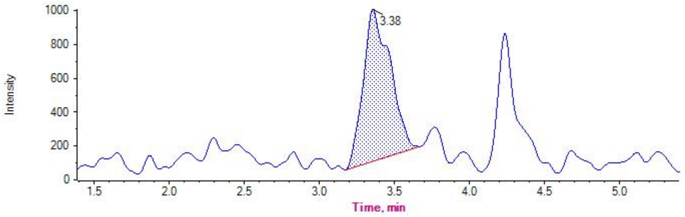

2

RT (Exp. RT): 3.38 (3.39) min

Calculated Conc: 1.90 ng/mL

Area: 4.614e4

Sample Type: (Standard)

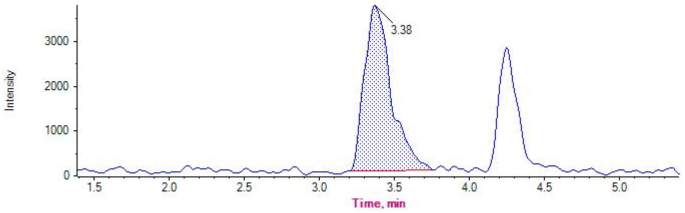

2

RT (Exp. RT): 3.37 (3.39) min

Calculated Conc: 1.91 ng/mL

Area: 4.644e4

Sample Type: (Standard)

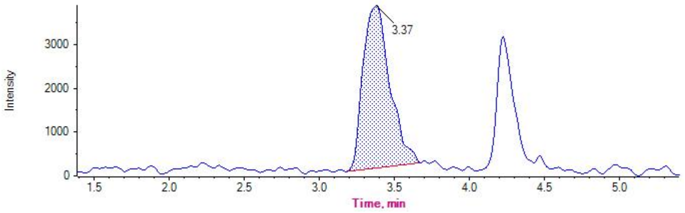

5

RT (Exp. RT): 3.38 (3.39) min

Calculated Conc: 4.33 ng/mL

Area: 1.056e5

Sample Type: (Standard)

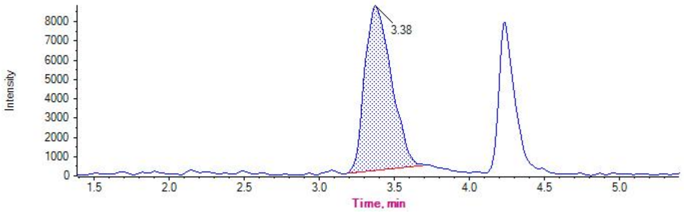

5

RT (Exp. RT): 3.38 (3.39) min

Calculated Conc: 4.74 ng/mL

Area: 1.156e5

Sample Type: (Standard)

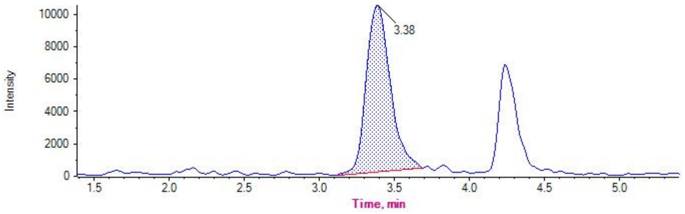

20

RT (Exp. RT): 3.38 (3.39) min

Calculated Conc: 21.83 ng/mL

Area: 5.331e5

Sample Type: (Standard)

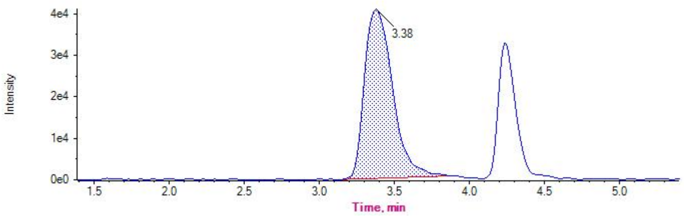

20

RT (Exp. RT): 3.37 (3.39) min

Calculated Conc: 18.77 ng/mL

Area: 4.584e5

Sample Type: (Standard)

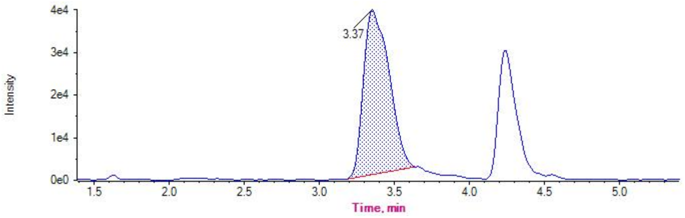

50

RT (Exp. RT): 3.37 (3.39) min

Calculated Conc: 49.18 ng/mL

Area: 1.202e6

Sample Type: (Standard)

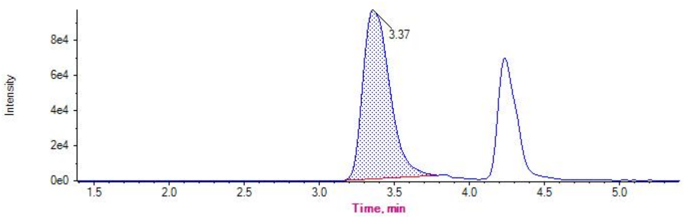

50

RT (Exp. RT): 3.37 (3.39) min

Calculated Conc: 48.12 ng/mL

Area: 1.176e6

Sample Type: (Standard)

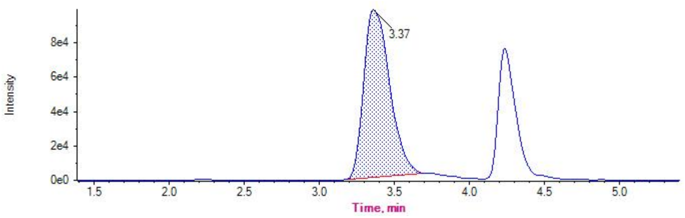

200

RT (Exp. RT): 3.37 (3.39) min

Calculated Conc: 221.44 ng/mL

Area: 5.412e6

Sample Type: (Standard)

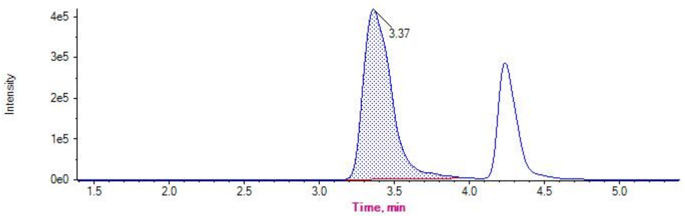

200

RT (Exp. RT): 3.38 (3.39) min

Calculated Conc: 225.51 ng/mL

Area: 5.511e6

Sample Type: (Standard)

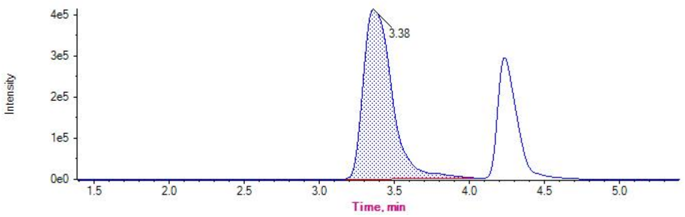

ABA

0.1

RT (Exp. RT): 6.76 (6.69) min

Calculated Conc: 0.09 ng/mL

Area: 7.665e3

Sample Type: (Standard)

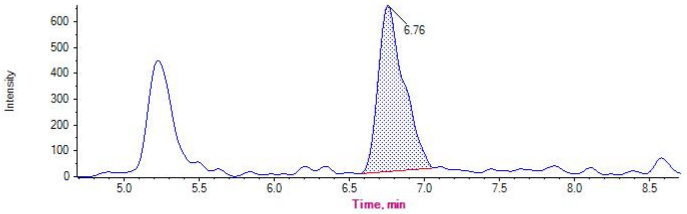

0.2

RT (Exp. RT): 6.73 (6.69) min

Calculated Conc: 0.19 ng/mL

Area: 1.017e4

Sample Type: (Standard)

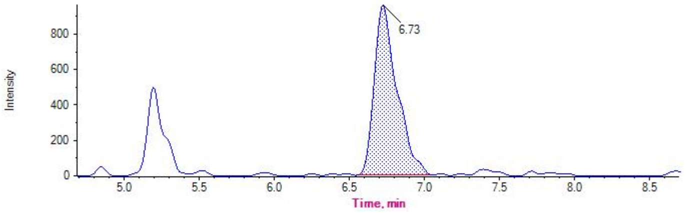

0.2

RT (Exp. RT): 6.74 (6.69) min

Calculated Conc: 0.23 ng/mL

Area: 1.138e4

Sample Type: (Standard)

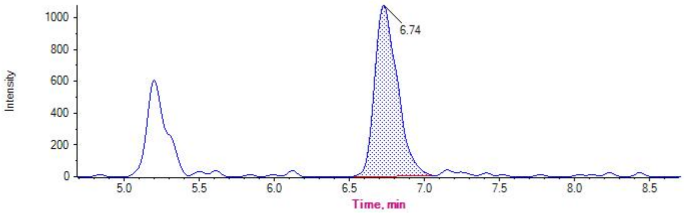

0.5

RT (Exp. RT): 6.74 (6.69) min

Calculated Conc: 0.43 ng/mL

Area: 1.683e4

Sample Type: (Standard)

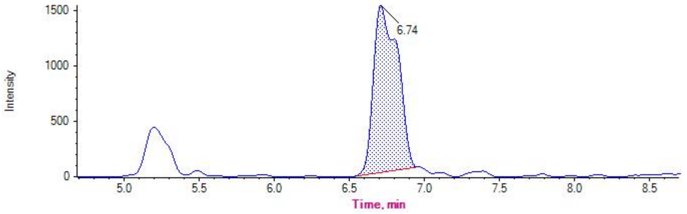

2

RT (Exp. RT): 6.72 (6.69) min

Calculated Conc: 2.12 ng/mL

Area: 6.282e4

Sample Type: (Standard)

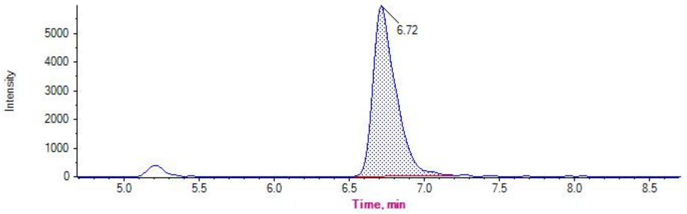

2

RT (Exp. RT): 6.72 (6.69) min

Calculated Conc: 2.06 ng/mL

Area: 6.099e4

Sample Type: (Standard)

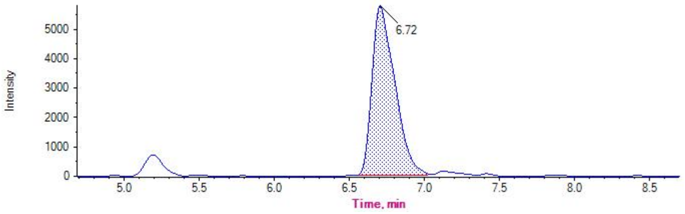

5

RT (Exp. RT): 6.72 (6.69) min

Calculated Conc: 4.83 ng/mL

Area: 1.364e5

Sample Type: (Standard)

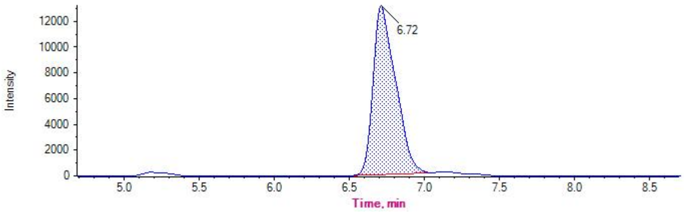

5

RT (Exp. RT): 6.72 (6.69) min

Calculated Conc: 5.45 ng/mL

Area: 1.531e5

Sample Type: (Standard)

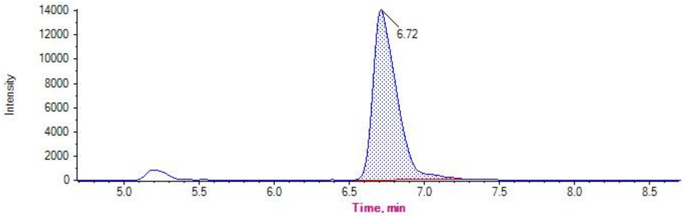

20

RT (Exp. RT): 6.70 (6.69) min

Calculated Conc: 18.93 ng/mL

Area: 5.194e5

Sample Type: (Standard)

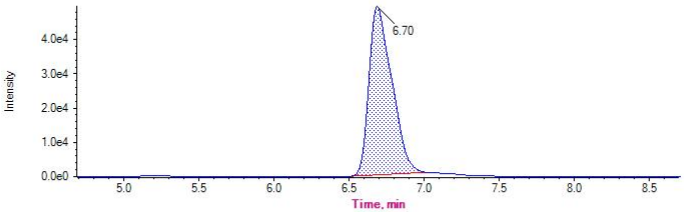

20

RT (Exp. RT): 6.71 (6.69) min

Calculated Conc: 20.54 ng/mL

Area: 5.632e5

Sample Type: (Standard)

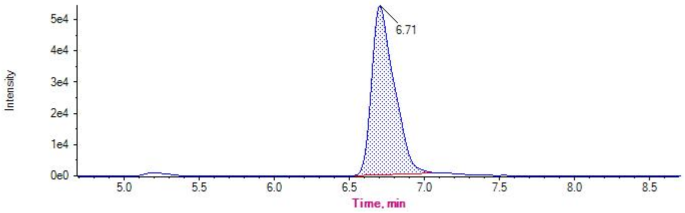

50

RT (Exp. RT): 6.71 (6.69) min

Calculated Conc: 47.24 ng/mL

Area: 1.289e6

Sample Type: (Standard)

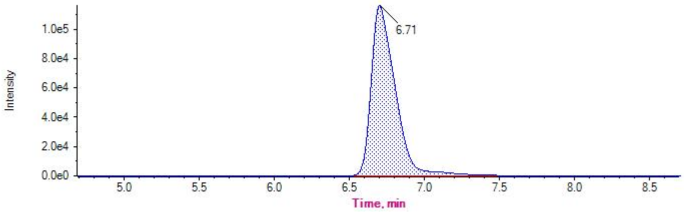

50

RT (Exp. RT): 6.71 (6.69) min

Calculated Conc: 47.75 ng/mL

Area: 1.302e6

Sample Type: (Standard)

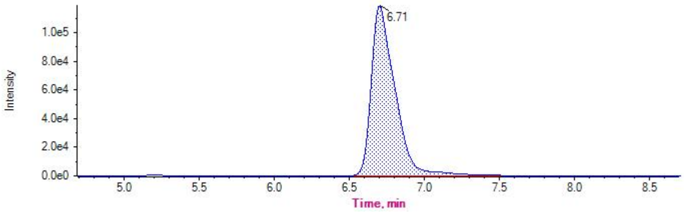

200

RT (Exp. RT): 6.71 (6.69) min

Calculated Conc: 189.74 ng/mL

Area: 5.160e6

Sample Type: (Standard)

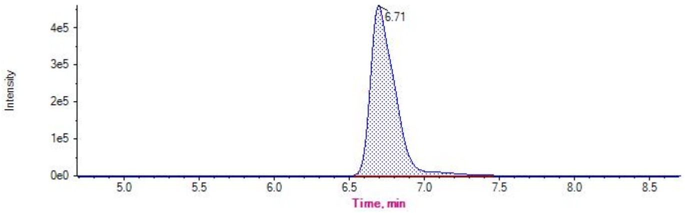

200

RT (Exp. RT): 6.71 (6.69) min

Calculated Conc: 189.83 ng/mL

Area: 5.163e6

Sample Type: (Standard)

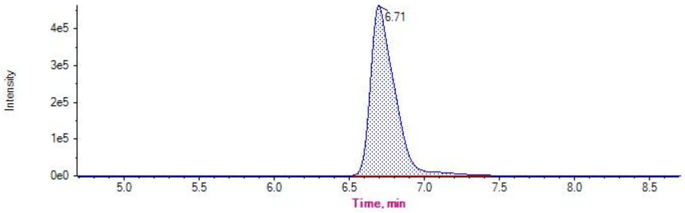

Sample raw data of 5-DS

Sample Name: DU **Vial #:** 9

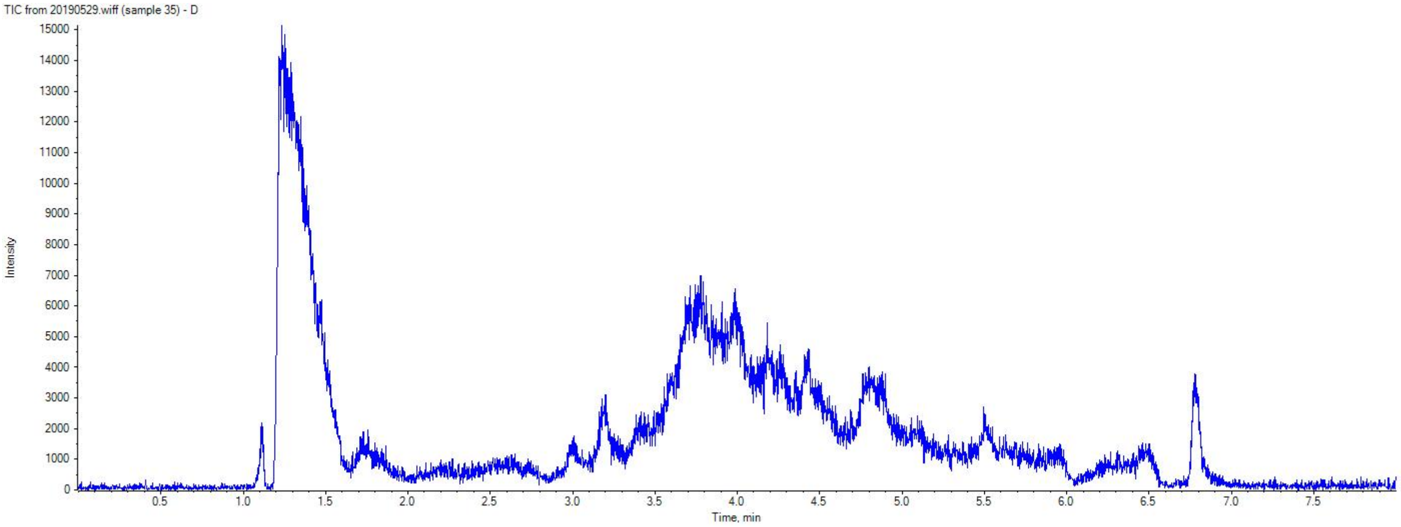

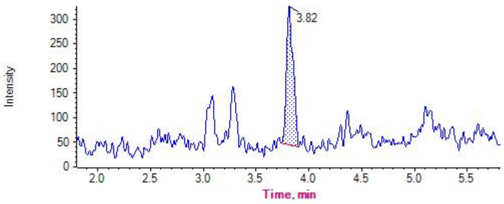

Compound Name:	5 DS 1 (331.0 / 234.1)
Expected RT:	3.81
Actual RT:	3.81
Equation:	y = 337.20896 x + -387.46192 (r = 0.99099) (weighting: 1 / x^2)
Area Counts:	8.294e2
ISTD Area Counts:	N/A
Amount:	3.61 (ng/mL)

Sample Name: DU **Vial #:** 9

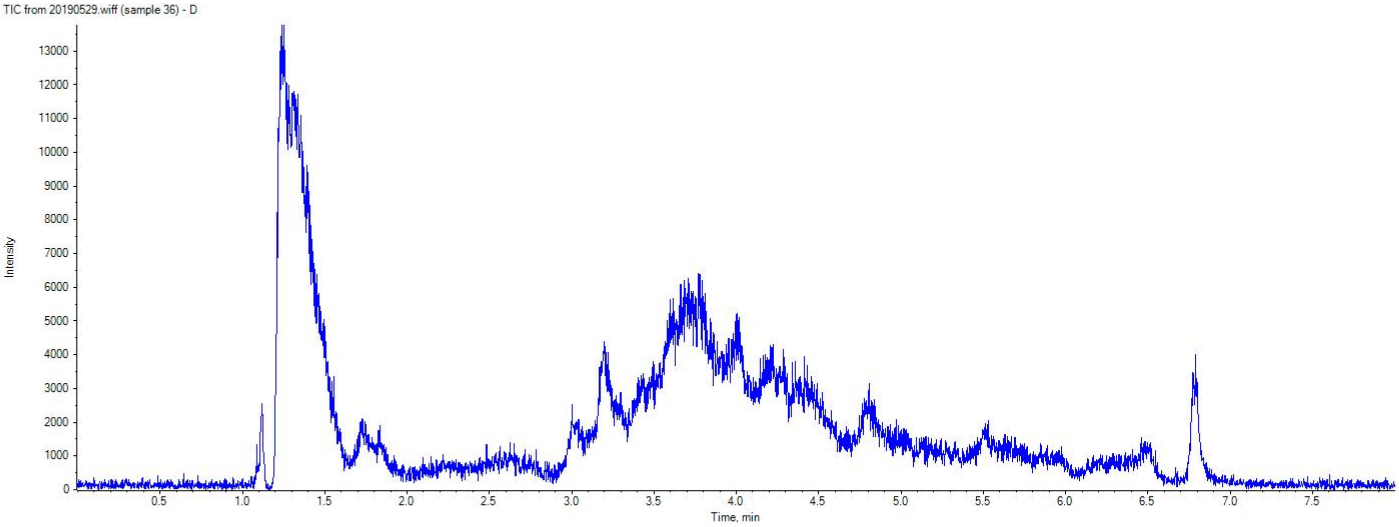

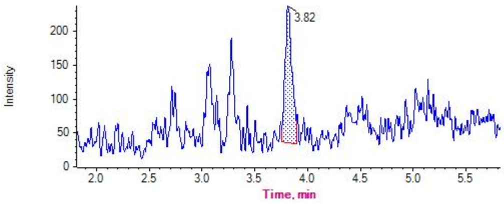

Compound Name:	5 DS 1 (331.0 / 234.1)
Expected RT:	3.82
Actual RT:	3.82
Equation:	y = 337.20896 x + -387.46192 (r = 0.99099) (weighting: 1 / x^2)
Area Counts:	9.148e2
ISTD Area Counts:	N/A
Amount:	3.86 (ng/mL)

Sample Name: DU **Vial #:** 9

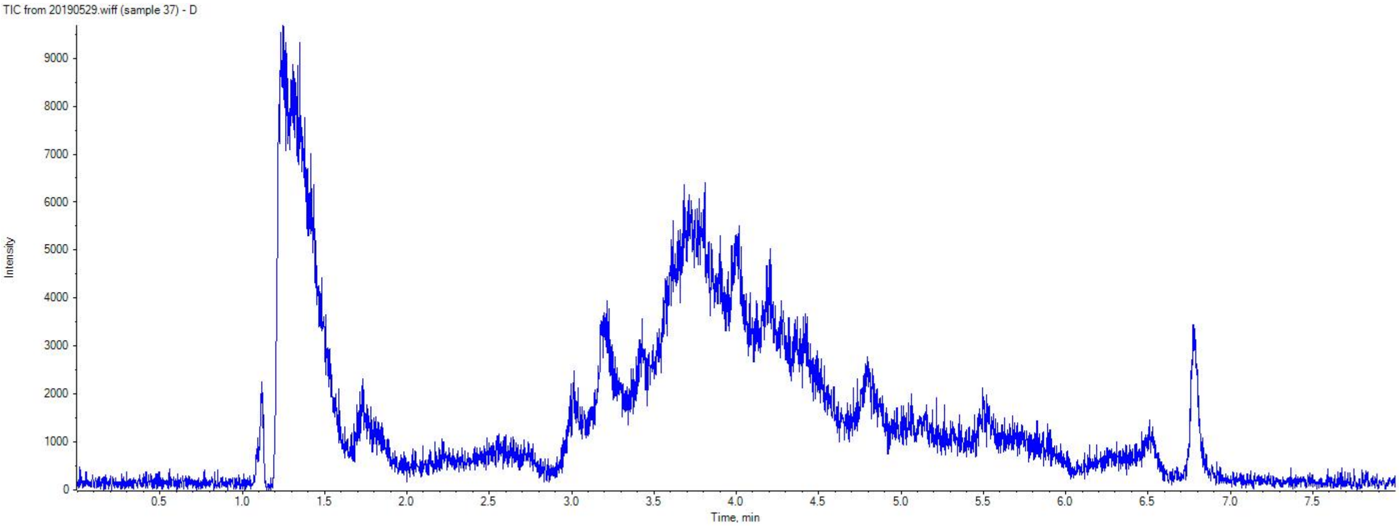

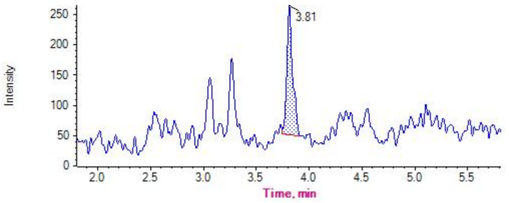

**Compound Name:**	5 DS 1 **(**331.0 / 234.1**)**
Expected RT:	3.81
Actual RT:	3.81
Equation:	y = 337.20896 x + -387.46192 (r = 0.99099) (weighting: 1 / x^2)
Area Counts:	8.139e2
ISTD Area Counts:	N/A
Amount:	3.56 (ng/mL)

Sample Name: SU **Vial #:** 10

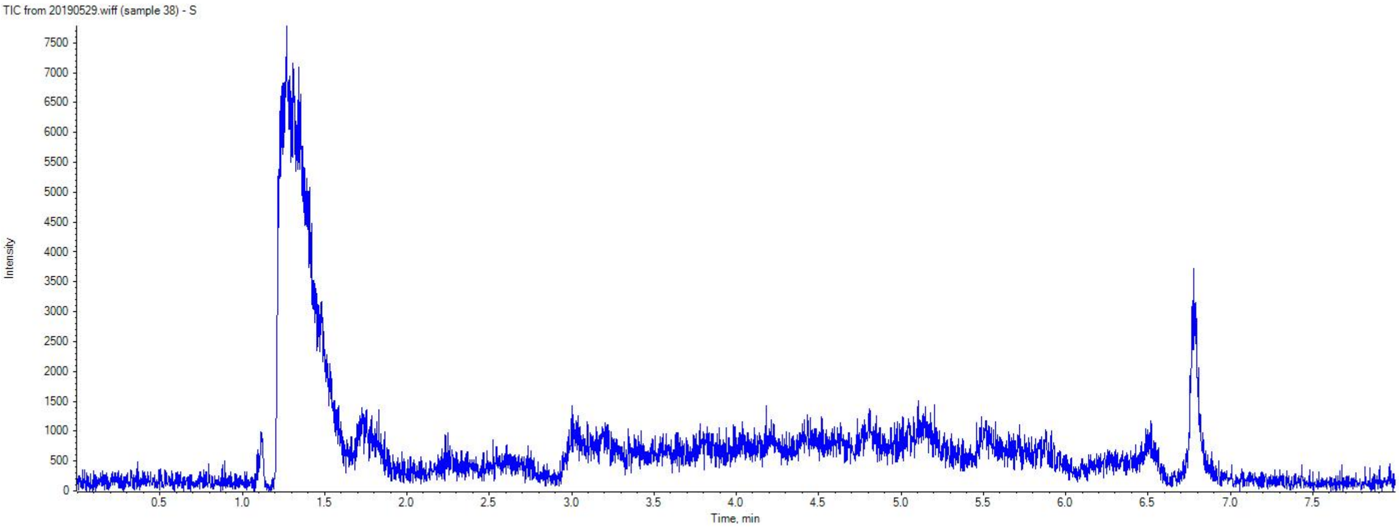

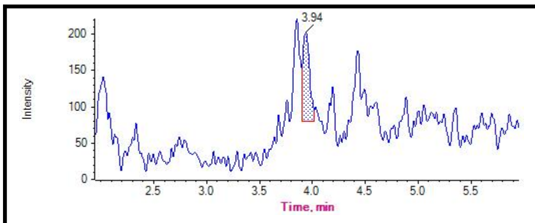

Compound Name:	5 DS 1 (331.0 / 234.1)
Expected RT:	3.95
Actual RT:	3.94
Equation:	y = 337.20896 x + -387.46192 (r = 0.99099) (weighting: 1 / x^2)
Area Counts:	5.468e2
ISTD Area Counts:	N/A
Amount:	2.77 (ng/mL)

Sample Name: SU **Vial #:** 10

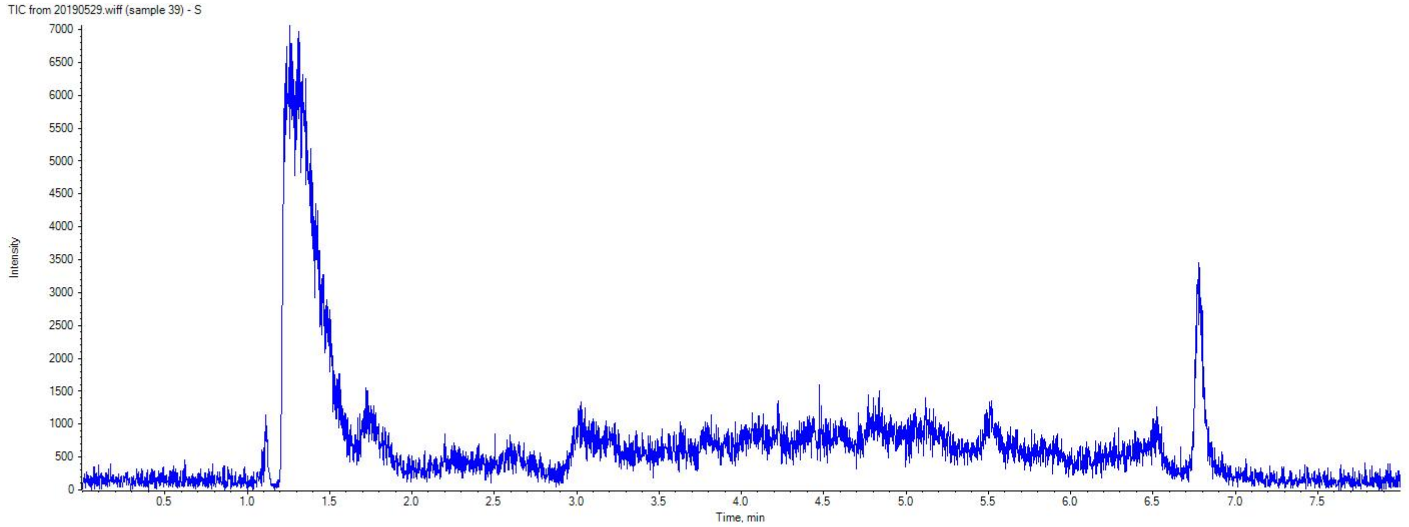

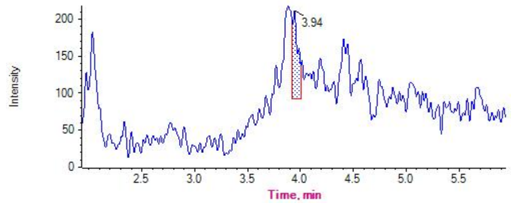

Compound Name:	5 DS 1 (331.0 / 234.1)
Expected RT:	3.94
Actual RT:	3.94
Equation:	y = 337.20896 x + -387.46192 (r = 0.99099) (weighting: 1 / x^2)
Area Counts:	4.284e2
ISTD Area Counts:	N/A
Amount:	2.42 (ng/mL)

5-DS

Sample Name: SU **Vial #:** 10

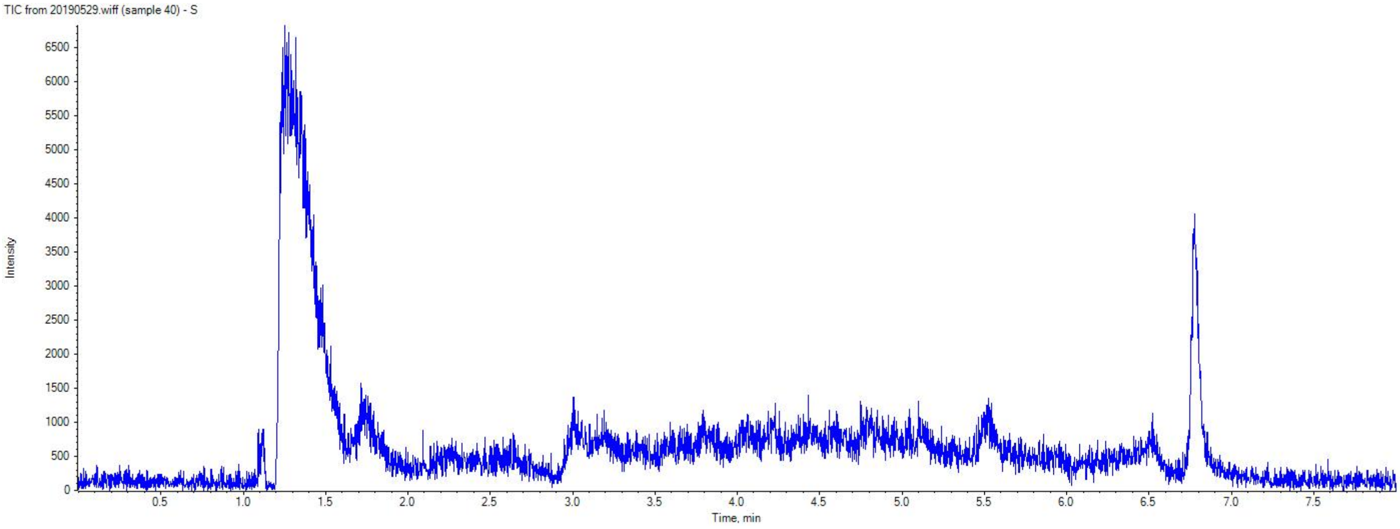

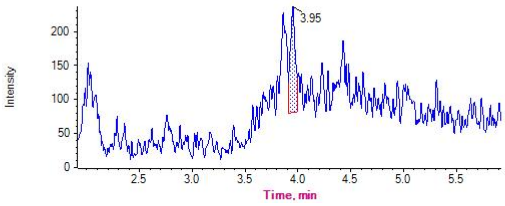

**Compound Name:**	5 DS 1 **(**331.0 / 234.1**)**
Expected RT:	3.92
Actual RT:	3.95
Equation:	y = 337.20896 x + -387.46192 (r = 0.99099) (weighting: 1 / x^2)
Area Counts:	5.217e2
ISTD Area Counts:	N/A
Amount:	2.70 (ng/mL)

Standard raw data

2

RT (Exp. RT):	3.90 (3.91) min
Calculated Conc:	2.14 ng/mL
Area:	3.341e2
Sample Type:	(Standard)

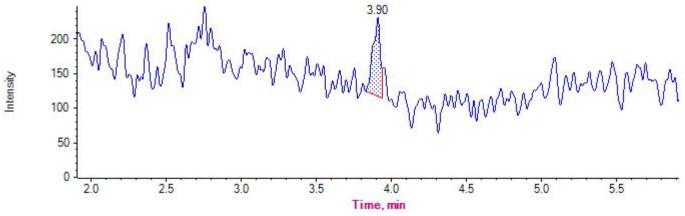

5

RT (Exp. RT):	3.92 (3.92) min
Calculated Conc:	4.60 ng/mL
Area:	1.165e3
Sample Type:	(Standard)

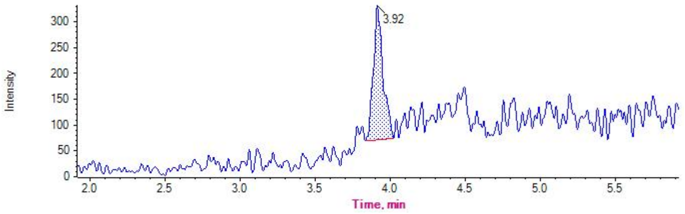

10

RT (Exp. RT): 3.92 (3.92) min

Calculated Conc: 11.01 ng/mL

Area: 3.326e3

Sample Type: (Standard)

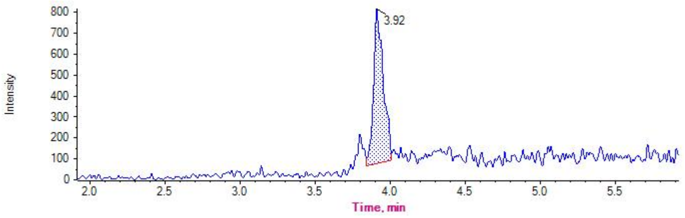

10

RT (Exp. RT): 3.92 (3.92) min

Calculated Conc: 11.35 ng/mL

Area: 3.439e3

Sample Type: (Standard)

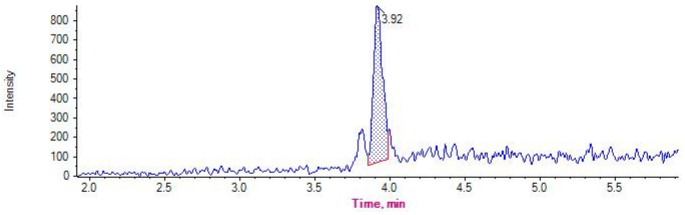

20

RT (Exp. RT): 3.92 (3.92) min

Calculated Conc: 18.47 ng/mL

Area: 5.842e3

Sample Type: (Standard)

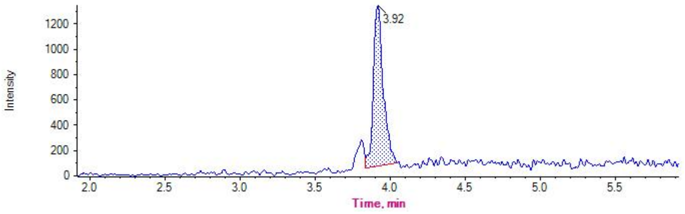

20

RT (Exp. RT): 3.91 (3.92) min

Calculated Conc: 18.43 ng/mL

Area: 5.828e3

Sample Type: (Standard)

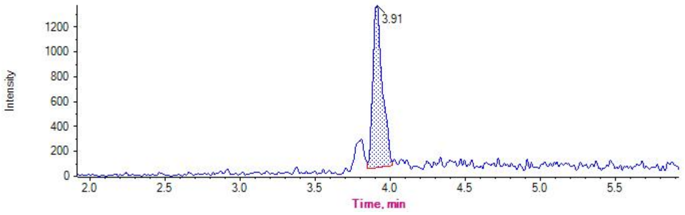

50

RT (Exp. RT): 3.92 (3.92) min

Calculated Conc: 49.35 ng/mL

Area: 1.625e4

Sample Type: (Standard)

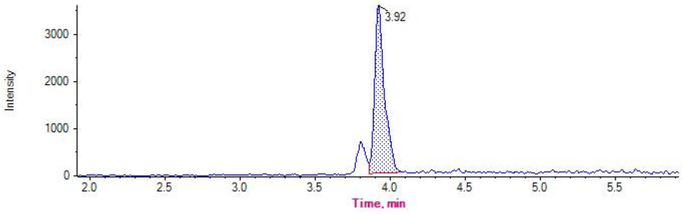

50

RT (Exp. RT): 3.92 (3.92) min

Calculated Conc: 50.54 ng/mL

Area: 1.666e4

Sample Type: (Standard)

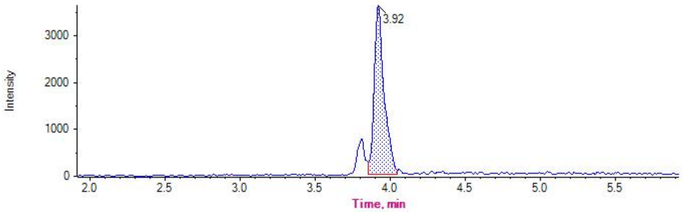

Supplement: Supplementary file 1 [file ijms-25-06149-s001.zip › sup.Fig.S1.pdf]
