# Supplementary material for: Endogenous Hormone Levels and Transcriptomic Analysis Reveal the Mechanisms of Bulbil Initiation in Pinellia ternata
Source: Int J Mol Sci. 2024 Jun 3;25(11):6149. doi: 10.3390/ijms25116149 (PMC11173086; doi:10.3390/ijms25116149)
Supplement: Supplementary file 1 [file ijms-25-06149-s001.zip › sup.Fig.S2.pdf]

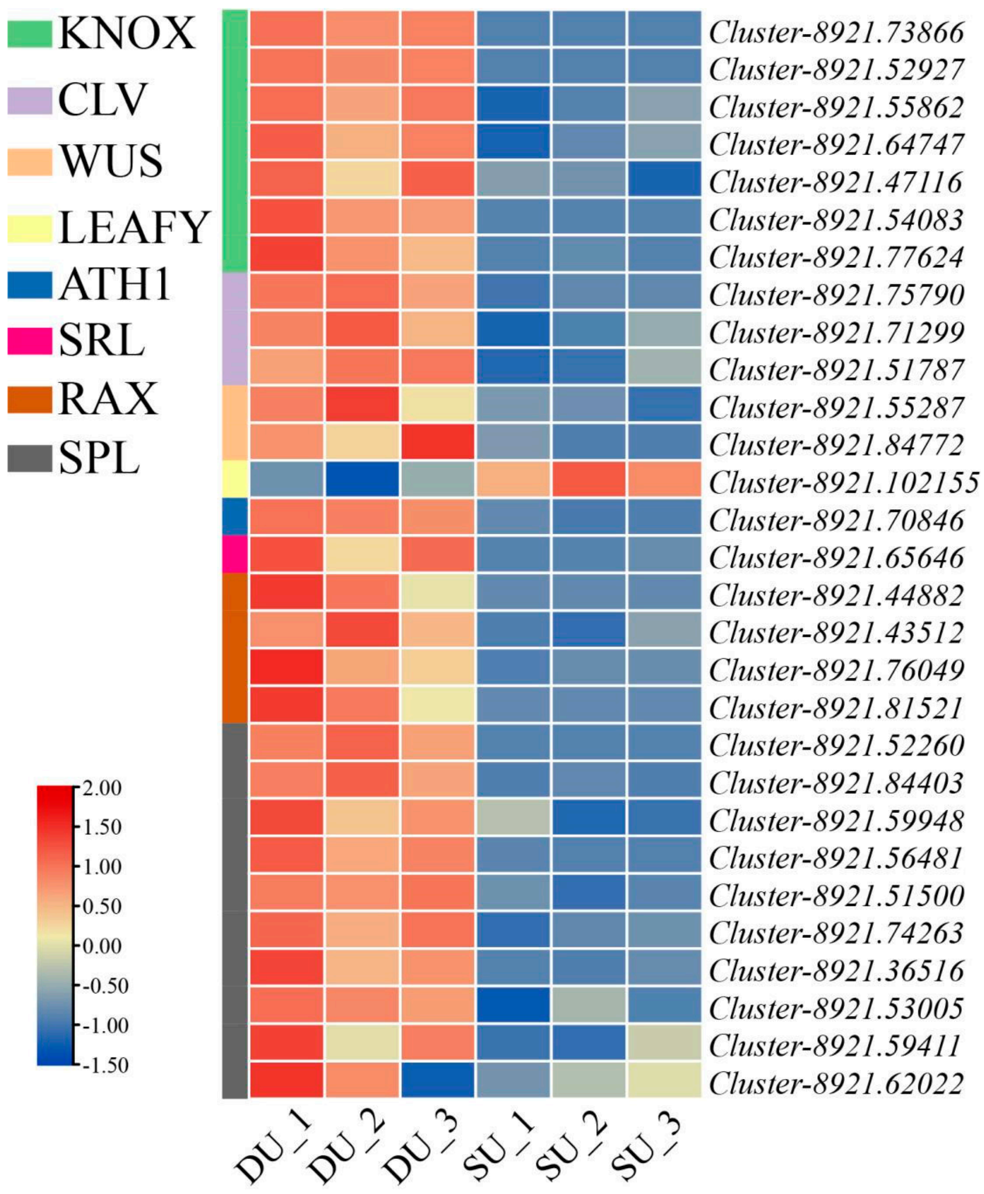

Sup. Figure S2 Heat map of differentially expressed transcription factors.

DU_1, DU_2, and DU_3 was the triple repetition of DU; SU_1, SU_2, and SU_3 was the triple repetition of SU; DU, the top of the petiole in DB; SU, the top of the petiole in SB; The FPKM value has a log2 numeric conversion and a row scale; the red indicated high expression level and blue indicated low expression level.
