# Supplementary material for: Endogenous Hormone Levels and Transcriptomic Analysis Reveal the Mechanisms of Bulbil Initiation in Pinellia ternata
Source: Int J Mol Sci. 2024 Jun 3;25(11):6149. doi: 10.3390/ijms25116149 (PMC11173086; doi:10.3390/ijms25116149)
Supplement: Supplementary file 1 [file ijms-25-06149-s001.zip › Sup.Fig.S3.pdf]

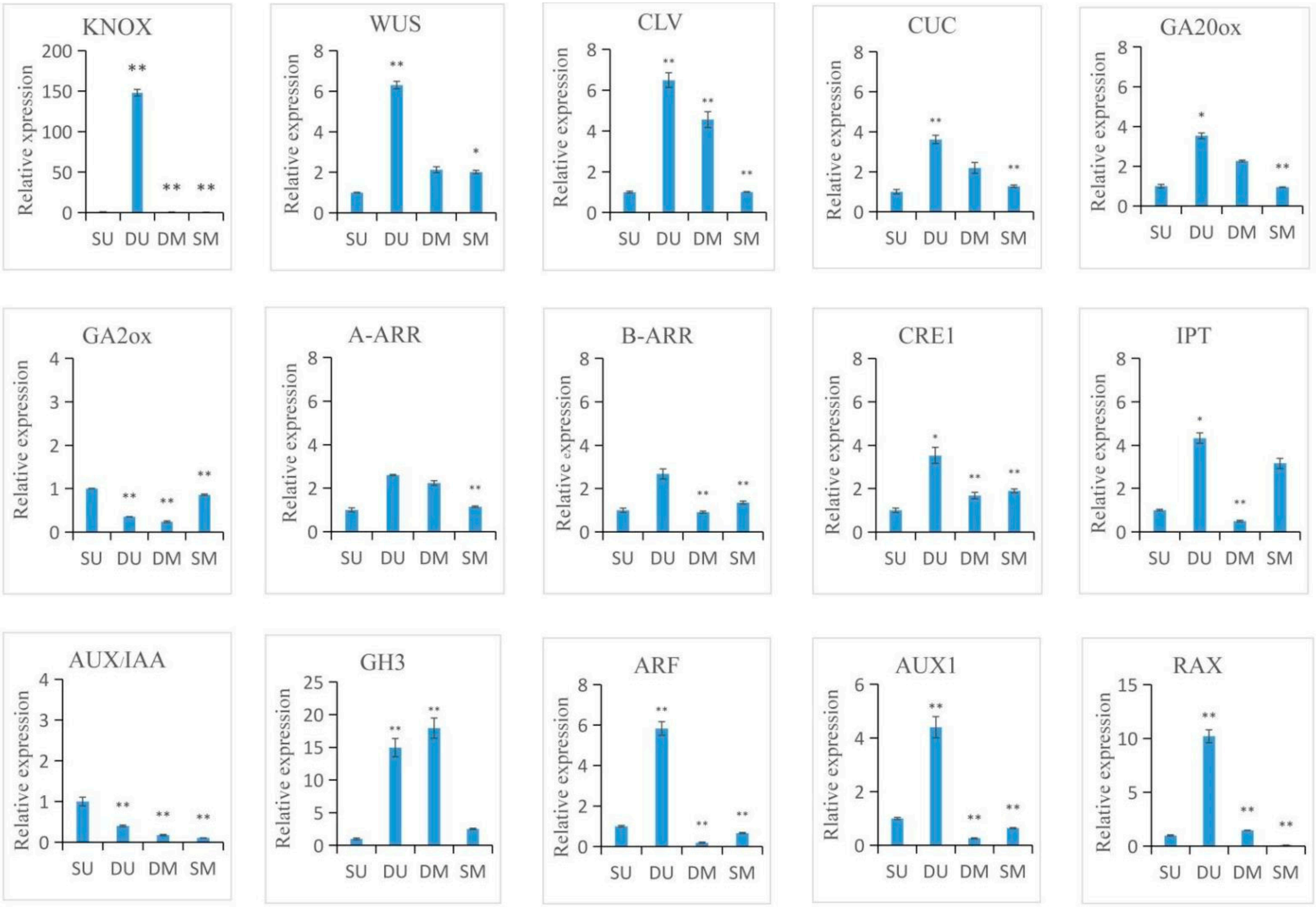

Sup. Figure S3. The relative expression of candidate genes

SU, the top of the petiole in SB; DU, the top of the petiole in DB; DM, the middle of the petiole in DB; SM, the middle of the petiole in SB.
