# Supplementary material for: Endogenous Hormone Levels and Transcriptomic Analysis Reveal the Mechanisms of Bulbil Initiation in Pinellia ternata
Source: Int J Mol Sci. 2024 Jun 3;25(11):6149. doi: 10.3390/ijms25116149 (PMC11173086; doi:10.3390/ijms25116149)
Supplement: Supplementary file 1 [file ijms-25-06149-s001.zip › sup.Fig.S4.pdf]

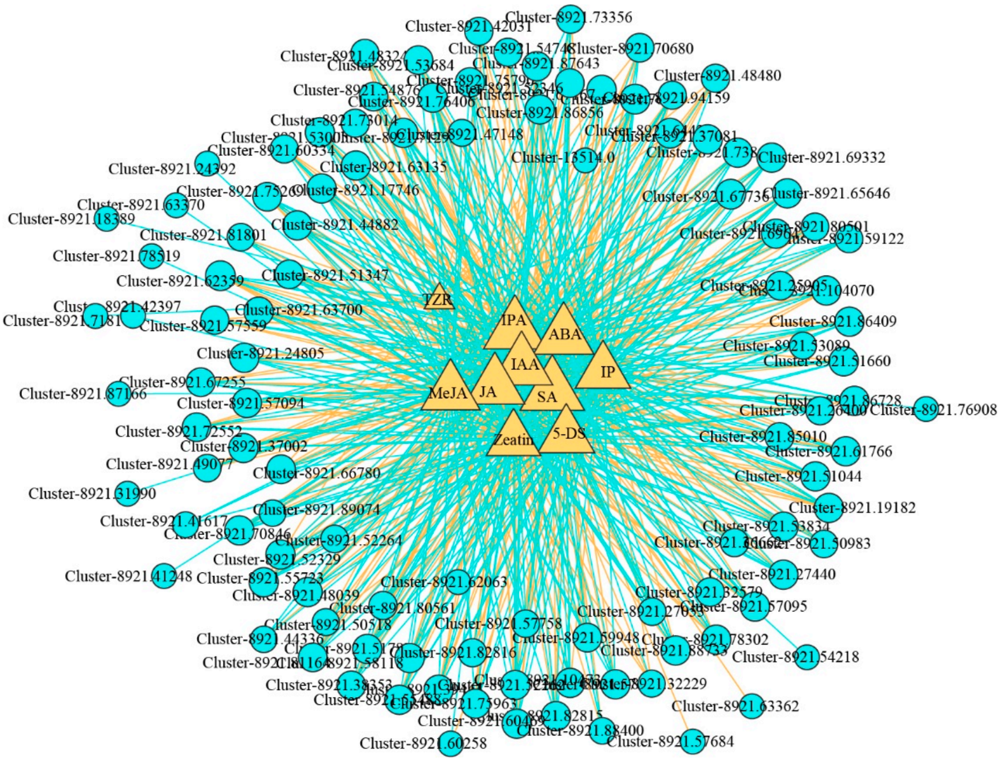

Figure S4 The Co-expression network of endogenous hormone and DEGs

Blue circles indicated genes, yellow triangles indicated hormones, blue lines indicated positive correlations, and yellow lines indicated negative correlations
