# Supplementary material for: Endogenous Hormone Levels and Transcriptomic Analysis Reveal the Mechanisms of Bulbil Initiation in Pinellia ternata
Source: Int J Mol Sci. 2024 Jun 3;25(11):6149. doi: 10.3390/ijms25116149 (PMC11173086; doi:10.3390/ijms25116149)
Supplement: Supplementary file 1 [file ijms-25-06149-s001.zip › Sup.Table S1.pdf]

Sup.Table S1 The results of quantitative hormone testing

ng/g

id	Compound	DU-1	DU-2	DU-3	Mean	SU-1	SU-2	SU-3	Mean	VIP	P-value	Q-value	Fold	Log foldchange
1	IAA	20.99200	20.92400	20.32000	20.74533	26.22000	25.64000	25.80000	25.88667	1.13564	4.80602E-05	3.41151E-05	1.247830837	0.319422368
2	IBA	0.11600	0.10219	0.12800	0.11540	0.14000	0.14800	0.09600	0.12800	0.38146	0.518036711	0.100529206	1.109223458	0.149550032
3	IP	0.00244	0.00575	0.00546	0.00455	0.02535	0.01173	0.01752	0.01820	0.99293	0.028803513	0.006815311	3.998792699	1.999564492
4	IPA	0.28000	0.30400	0.27200	0.28533	0.07200	0.06800	0.06400	0.06800	1.14140	2.53604E-05	2.65502E-05	0.238317757	-2.069041644
5	ZEATIN	0.15600	0.13200	0.12000	0.13600	0.24400	0.21200	0.22000	0.22533	1.08708	0.003345727	0.00101783	1.656862745	0.728454094
6	TZR	0.29600	0.28000	0.26400	0.28000	0.26400	0.25200	0.25600	0.25733	0.87578	0.083646808	0.017812796	0.919047619	-0.12178848
7	MEJA	0.00737	0.00800	0.00436	0.00657	0.02960	0.03013	0.01793	0.02589	1.04296	0.009537865	0.00253889	3.937541977	1.977295304
8	SA	41.39200	42.94400	40.93600	41.75733	8.99200	9.25200	9.89200	9.37867	1.14374	1.05815E-06	2.25335E-06	0.224599272	-2.154574843
9	ABA	2.72400	2.67600	2.53200	2.64400	1.63600	1.59600	1.50000	1.57733	1.13801	0.000110629	5.52359E-05	0.596570852	-0.745234604
10	JA	4.34400	4.48400	4.36000	4.39600	3.08000	2.76400	2.81949	2.88783	1.13148	0.000146975	6.25972E-05	0.656922392	-0.606205153
11	MESA	0.08400	0.08800	0.09600	0.08933	0.07200	0.09600	0.08400	0.08400	0.37452	0.530411676	0.102684076	0.940298507	-0.088809267
12	5DS	0.27700	0.24200	0.27000	0.26300	0.36086	0.38600	0.35600	0.36762	1.10267	0.001793381	0.000633404	1.39779831	0.483156208

The differential metabolite were selected based on a VIP ≥ 1 and p ≤ 0.05.

DU_1, DU_2, and DU_3 was the triple repetition of DU; SU_1, SU_2, and SU_3 was the triple repetition of SU; DU, the top of the petiole in DB; SU, the top of the petiole in SB.
