# Supplementary material for: Endogenous Hormone Levels and Transcriptomic Analysis Reveal the Mechanisms of Bulbil Initiation in Pinellia ternata
Source: Int J Mol Sci. 2024 Jun 3;25(11):6149. doi: 10.3390/ijms25116149 (PMC11173086; doi:10.3390/ijms25116149)
Supplement: Supplementary file 1 [file ijms-25-06149-s001.zip › Sup.Table S11.pdf]

Sup.Table S11 selected reaction monitoring conditions for protonated or deprotonated plant hormones([M+H]+or[M-H]-)

Component	Polarity	Parent ion（m/z）	Daughter ion（m/z）	Uncluster voltage(V)	Collision energy（V）
IBA	-	202	116/158/184	-80	-0.058479532
JA	-	209.2	58.9	-54	-16
MeJA	-	225.1	151.1/193.1/133	-50	-0.088888889
MeSA	-	153	121.0/93.1/65.0	-131	-0.012820513
SA	-	137	92.9/65	-50	0.512820513
ABA	-	263.1	153.0/204.2	-60	0.518518519
IP	+	204.1	136.1/148.2	51	24/29
IPA	+	336.2	204.2/136.3	59	28/32
Zeatin	+	220.4	136/202.1	92	22/16
TZR	+	352.3	220.2/136/202.1	90	25/40/32
IAA	+	176.2	129.8/102.9	65	12/42

IP, isopentenyladenine; SA, salicylic acid; MESA, methyl salicylate; IPA, isopentenyl adenosine; ABA, abscisic acid; JA, jasmonic acid; IAA, indole-3-acetic acid; IBA, indole-3-butyric acid; MeJA, methyl jasmonate; TZR, trans-zeatin nucleoside.
