# Supplementary material for: Endogenous Hormone Levels and Transcriptomic Analysis Reveal the Mechanisms of Bulbil Initiation in Pinellia ternata
Source: Int J Mol Sci. 2024 Jun 3;25(11):6149. doi: 10.3390/ijms25116149 (PMC11173086; doi:10.3390/ijms25116149)
Supplement: Supplementary file 1 [file ijms-25-06149-s001.zip › Sup.Table S12.pdf]

Sup.Table S12 Selected reaction monitoring conditions for protonated or deprotonated 5-DS([M+H]+or[M-H]-)

Component	Polarity	Parent ion （m/z）	Daughter ion （m/z）	Uncluster voltage(V)	Collision energy（V）
5-DS	+	331	234.1/217.2	45	14/16

5-DS, 5-dexoxystrigol
