# Supplementary material for: Endogenous Hormone Levels and Transcriptomic Analysis Reveal the Mechanisms of Bulbil Initiation in Pinellia ternata
Source: Int J Mol Sci. 2024 Jun 3;25(11):6149. doi: 10.3390/ijms25116149 (PMC11173086; doi:10.3390/ijms25116149)
Supplement: Supplementary file 1 [file ijms-25-06149-s001.zip › Sup.Table S13.pdf]

Table S13 The qRT-PCR Primer Sequences of DEGs

Gene ID	Gene Name	Primer sequences	Amplicon size (bp)	Primer TM（℃）	Melt Temp（℃）
Cluster-8921.38353	*ARF*	F: GGAATTATTGGTTGTGAAGGTGC<br>R: TGGCAGACGACAGCAGCAACT	238	58<br>64	82. 5
Cluster-8921.27053	*GA2ox*	F: TAGCGGTACCCGAACGAGAGG<br>R: CCCATGGGTTCTTCCGCGT	214	62<br>62	87
Cluster-8921.37002	*IPT*	F: CGTCCAGGAAGGGGAGGACATG<br>R: TCGGTGACGGGTAGCAG	165	62<br>58	84. 5
Cluster-8921.41248	*AUX1*	F: GGATCTCCATGTTCCTCTGGCACG<br>R: ACTTCGAACCACTGGATGACATG	265	62<br>59	86
Cluster-8921 .42397	*CUC*	F: GACCAACTGGGTCATGCACGA<br>R: TGGTGGTGGTGGTGGAATGC	222	60<br>62	90
Cluster-8921.43512	*RAX*	F: CCGGTGTTAATTTGATCCCGTACG<br>R: GGACTTCCGACAGTGCTCCG	204	60<br>61	80
Cluster-8921.49077	*CRE1*	F: GCGACCCATGAGAGTCTTAAGACAAAGG<br>R: CTCACTTCTCACCCTCTTCC	221	62<br>58	88
Cluster-8921. 51660	*KNOX1*	F: CGCCGTCCTCAGAACACG<br>R: AGTGCGGATGAGACATGATCTTC	231	60<br>58	88. 5
Cluster-8921. 51787	*CLV*	F: GCGCCGGTGAGGAGGTT<br>R: CTTCCCCAGCTCGAGTACATCG	197	62<br>60	84
Cluster-8921.53834	*GA20ox*	F: TCCCTGTGACTGAATTGGAATAACTG<br>R: CATTTCTCTTCAACACAGCTGGTT	269	59<br>58	85. 5
Cluster-8921. 55287	*WUS*	F: GCTTGGGGTGCCAGTGCC<br>R: GGCCGTCACCGCCCAGCAAGAC	175	65<br>69	81. 5
Cluster-8921. 57684	*AUX*	F: CCTTCAGGAAGAACATCTTCTCCG<br>R: CCAAGGCCATGGATAGCTCCTGGT	183	60<br>65	87
Cluster-8921 .67255	*A-ARR*	F: AGCAGCAGACACAGCAGTC<br>R: GCTGTCAAGCTGCTACTGTGTATC	225	60<br>60	81
Cluster-8921.85010	*B-ARR*	F: GCCTGTCCGGTTTACACTTCC<br>R: CGTTGGAGGTTCCTATGATAGACACT	201	59<br>60	85. 5
Cluster-8921. 94465	*GH3*	F: CCTCTTCCGTCACGGACTCC<br>R: GCCTCGACGTACTTGGCCC	169	60<br>62	78. 5
PteTubulin		F: CTCGCGGCATGGATCTGCA<br>R: CCGAGGCCGACGAGAACTGA	239	62<br>62	87.5
