# Supplementary material for: Endogenous Hormone Levels and Transcriptomic Analysis Reveal the Mechanisms of Bulbil Initiation in Pinellia ternata
Source: Int J Mol Sci. 2024 Jun 3;25(11):6149. doi: 10.3390/ijms25116149 (PMC11173086; doi:10.3390/ijms25116149)
Supplement: Supplementary file 1 [file ijms-25-06149-s001.zip › Sup.Table S2.pdf]

Sup.Table S2 The clean Illumina reads

sample	library	raw_reads	raw_bases	clean_reads	clean_bases	error_rate	Q20	Q30	GC_pct
DU_1	FRAS220098690-1r	23122402	6.9	20862794	6.3	0.03	96.49	91.27	53.97
DU_2	FRAS220098690-3r	18595954	5.6	18547759	5.6	0.03	96.62	91.48	53.79
DU_3	FRAS220098690-2r	20952140	6.3	19199287	5.8	0.03	96.79	91.96	53.44
DM_1	FRAS220098691-1r	23190640	7	23128264	6.9	0.03	96.8	91.84	52.63
DM_2	FRAS220098691-2r	23153718	6.9	23094402	6.9	0.03	96.77	91.75	52.93
DM_3	FRAS220098691-3r	23820331	7.1	21405675	6.4	0.03	96.63	91.45	53.16
SU_1	FRAS220098692-3r	23940495	7.2	21903333	6.6	0.03	97.05	92.37	52.12
SU_2	FRAS220098692-1r	23117382	6.9	23043606	6.9	0.03	96.79	91.83	54.24
SU_3	FRAS220098692-2r	24646967	7.4	22269442	6.7	0.03	96.93	92.13	53.4
SM_1	FRAS220098693-3r	23104404	6.9	21353194	6.4	0.03	97.12	92.7	53.52
SM_2	FRAS220098693-1r	22974999	6.9	22909878	6.9	0.03	96.68	91.54	52.05
SM_3	FRAS220098693-2r	20679433	6.2	20615816	6.2	0.03	96.6	91.46	53.85

SU, the top of the petiole in SB; DU, the top of the petiole in DB; DM, the middle of the petiole in DB; SM, the middle of the petiole in SB.
