# Supplementary material for: Endogenous Hormone Levels and Transcriptomic Analysis Reveal the Mechanisms of Bulbil Initiation in Pinellia ternata
Source: Int J Mol Sci. 2024 Jun 3;25(11):6149. doi: 10.3390/ijms25116149 (PMC11173086; doi:10.3390/ijms25116149)
Supplement: Supplementary file 1 [file ijms-25-06149-s001.zip › Sup.Table S3.pdf]

Sup.Table S3 The genes annotated in GO pathway

GO ID	GO Term (Lev2)	GO Term (Lev1)	Gene number
GO:0005198	structural molecule activity	Molecular Function	1089
GO:0016209	antioxidant activity	Molecular Function	168
GO:0140299	small molecule sensor activity	Molecular Function	97
GO:0005488	binding	Molecular Function	18346
GO:0140110	transcription regulator activity	Molecular Function	1427
GO:0060089	molecular transducer activity	Molecular Function	250
GO:0003824	catalytic activity	Molecular Function	14930
GO:0140104	molecular carrier activity	Molecular Function	26
GO:0005215	transporter activity	Molecular Function	2520
GO:0045182	translation regulator activity	Molecular Function	162
GO:0038024	cargo receptor activity	Molecular Function	8
GO:0098772	molecular function regulator	Molecular Function	1315
GO:0050789	regulation of biological process	Biological Process	6831
GO:0040011	locomotion	Biological Process	215
GO:0110148	biomineralization	Biological Process	13
GO:0051179	localization	Biological Process	5049
GO:0009987	cellular process	Biological Process	20249
GO:0048519	negative regulation of biological	Biological Process	518
GO:0048518	positive regulation of biological	Biological Process	420
GO:0050896	response to stimulus	Biological Process	4002
GO:0000003	reproduction	Biological Process	643
GO:0032501	multicellular organismal process	Biological Process	992
GO:0007610	behavior	Biological Process	53
GO:0040007	growth	Biological Process	47
GO:0048511	rhythmic process	Biological Process	23
GO:0043473	pigmentation	Biological Process	3
GO:0051703	intraspecies interaction between	Biological Process	35
GO:0023052	signaling	Biological Process	2075
GO:0098754	detoxification	Biological Process	50
GO:0022414	reproductive process	Biological Process	608
GO:0044419	interspecies interaction between	Biological Process	2127
GO:0008152	metabolic process	Biological Process	18437

GO:0032502	developmental process	Biological Process	811
GO:0065007	biological regulation	Biological Process	7278
GO:0019740	nitrogen utilization	Biological Process	3
GO:0022610	biological adhesion	Biological Process	247
GO:0002376	immune system process	Biological Process	306
GO:0051704	multi-organism process	Biological Process	629
GO:0032991	protein-containing complex	Cellular Component	5818
GO:0044423	virion part	Cellular Component	741
GO:0110165	cellular anatomical entity	Cellular Component	14386
GO:0019012	virion	Cellular Component	741
GO:0005622	intracellular	Cellular Component	7783
