# Supplementary material for: Endogenous Hormone Levels and Transcriptomic Analysis Reveal the Mechanisms of Bulbil Initiation in Pinellia ternata
Source: Int J Mol Sci. 2024 Jun 3;25(11):6149. doi: 10.3390/ijms25116149 (PMC11173086; doi:10.3390/ijms25116149)
Supplement: Supplementary file 1 [file ijms-25-06149-s001.zip › Sup.Table S4.pdf]

Sup.Table S4 The results of the pairwise comparisons between SB and DB libraries

Comparison groups	All	Up	Down	Threshold
DU vs SU	9112	4573	4539	DESeq2 padj<0.05 |log2FoldChange|>1
DU vs DM	3630	3201	429	DESeq2 padj<0.05 |log2FoldChange|>1
SU vs SM	1021	695	326	DESeq2 padj<0.05 |log2FoldChange|>1
DM vs SM	7552	3409	4143	DESeq2 padj<0.05 |log2FoldChange|>1

SU, the top of the petiole in SB; DU, the top of the petiole in DB; DM, the middle of the petiole in DB; SM, the middle of the petiole in SB; SB, single-bulbil type; DB, double-bulbil type.
