# Supplementary material for: Endogenous Hormone Levels and Transcriptomic Analysis Reveal the Mechanisms of Bulbil Initiation in Pinellia ternata
Source: Int J Mol Sci. 2024 Jun 3;25(11):6149. doi: 10.3390/ijms25116149 (PMC11173086; doi:10.3390/ijms25116149)
Supplement: Supplementary file 1 [file ijms-25-06149-s001.zip › Sup.Table S6.pdf]

Table S6 The DEGs information of hormone synthesis pathways

hormone	Gene name	Gene_ID	log2FoldChange	pval	padj	up or down	Description
5-DS	D27	Cluster-8921.57758	-1.1029	0.0019472	0.026128	down	beta-carotene isomerase D27 [Juglans regia]
	D14	Cluster-8921.72552	1.1679	0.0033434	0.039947	up	strigolactone esterase D14-like [Phoenix dactylifera]
		Cluster-8921.52346	-1.6984	0.0000161	0.00048847	down	strigolactone esterase D14 [Phoenix dactylifera]
		Cluster-8921.61766	1.0445	0.000077	0.0018515	up	PREDICTED: strigolactone esterase D14 [Zea mays]
		Cluster-8921.27440	5.9043	0.0001795	0.0037644	up	strigolactone esterase D14 [Vitis vinifera]
	CYP711A1	Cluster-8921.79128	1.8142	0.00024424	0.0048728	up	Cytochrome P450 711A1 like [Actinidia chinensis var. chinensis]
		Cluster-8921.6134	-5.2782	0.0036643	0.042869	down	cytochrome P450 711A1 [Phoenix dactylifera]
	SMAX	Cluster-8921.40244	3.7718	0.00004457	0.0011622	up	protein SMAX1-LIKE 4-like

							[Phoenix dactylifera]
CTK	IPT	Cluster-8921.37002	2.1445	0.0000357	0.00095867	up	adenylate isopentenyltransferase [Eucalyptus grandis]
		Cluster-8921.7088	-2.6666	0.00017833	0.003743	down	Adenylate isopentenyltransferase 5 [Arabidopsis thaliana]
JAs	LOX	Cluster-8921.7181	-3.9353	0.00035832	0.0066807	down	lipoxygenase
		Cluster-8921.83767	-3.9149	0.0013425	0.019466	down	linoleate 9S-lipoxygenase 5 [Manihot esculenta]
		Cluster-8921.63135	3.8863	3.9E-24	3.12E-21	up	probable linoleate 9S-lipoxygenase 4 [Elaeis guineensis]
	AOS	Cluster-8921.58118	1.9415	4.52E-14	9.13E-12	up	allene oxide synthase-like [Hevea brasiliensis]
		Cluster-8921.60469	1.3746	1.42E-09	0.000000125	up	allene oxide synthase 1 [Elaeis guineensis]
		Cluster-8921.86856	2.1076	0.00011848	0.0026521	up	Allene oxide synthase 1,OS=Oryza sativa subsp.

	Cluster-8921.36662	2.0457	0.00041664	0.007546	up	allene oxide synthase 2-like [Phoenix dactylifera]
	Cluster-8921.25905	1.278	0.0033289	0.039814	up	PREDICTED: allene oxide synthase 2-like [Musa acuminata subsp. malaccensis]
AOC	Cluster-8921.66780	1.7486	0.00000003	0.00000196	up	allene oxide cylase [Lilium longiflorum]
	Cluster-8921.55723	2.0465	5.78E-08	0.00000353	up	allene oxide cyclase [Chenopodium quinoa]
	Cluster-8921.80501	3.2486	0.0000523	0.0013273	up	allene oxide cyclase [Pisum sativum]
	Cluster-8921.52264	1.5147	0.00014783	0.0031936	up	allene oxide cyclase [Vigna radiata]
	Cluster-8921.70680	1.1427	0.00066532	0.011031	up	allene oxide cyclase [Sorghum bicolor]
	Cluster-8921.52262	1.5694	0.0024687	0.031533	up	PREDICTED: allene oxide cyclase 2, chloroplastic-like [Erythranthe guttata]
JMT	Cluster-8921.57682	1.3254	0.00047646	0.0084123	up	jasmonate O-methyltransferase

							[Hevea brasiliensis]
	OPR	Cluster-8921.23729	1.8195	2.8042E-06	0.00010833	up	12-oxophytodienoate reductase 3-like [Chenopodium quinoa]
		Cluster-8921.54218	1.6859	0.0033975	0.04045	up	9-cis-epoxycarotenoid dioxygenase [Oncidium hybrid cultivar]
		Cluster-8921.75963	3.4105	5.59E-10	5.39E-08	up	9-cis-epoxycarotenoid dioxygenase NCED1 [Phoenix dactylifera]
	NCED						
ABA		Cluster-8921.88400	-2.0612	0.0003839	0.0070677	down	9-cis-epoxycarotenoid dioxygenase NCED1 [Phoenix dactylifera]
		Cluster-8921.87166	4.5793	0.00070002	0.011488	up	Putative 9-cis-epoxycarotenoid dioxygenase NCED5,[Glycine soja]
	MCSU	Cluster-8921.55329	-1.1722	0.000014222	0.00043991	down	molybdenum cofactor sulfurase isoform X4 [Phoenix dactylifera]
Auxin	YUCCA	Cluster-8921.104070	-6.8863	0.000000513	0.0000243	down	indole-3-pyruvate monooxygenase YUCCA5

							[Elaeis guineensis]
	TDC	Cluster-8921.50137	7.1366	5.1699E-15	1.2014E-12	up	Pyridoxal phosphate-dependent decarboxylase [Cinnamomum micranthum f. kanehirae]
		Cluster-8921.80448	6.5278	0.000021276	0.00062402	up	tryptophan decarboxylase TDC1 [Solanum lycopersicum]
	ALDH	Cluster-8921.52329	1.5699	0.00000166	0.000068	up	aldehyde dehydrogenase 22A1 [Elaeis guineensis]
		Cluster-8921.76406	1.2715	0.00000674	0.0002304	up	putative Aldehyde dehydrogenase [Zostera marina]
		Cluster-8921.78519	3.6535	0.00052867	0.0091596	up	aldehyde dehydrogenase family 7 member A1 [Phoenix dactylifera]
		Cluster-8921.31990	3.4723	0.0041264	0.046899	up	Aldehyde dehydrogenase 22A1[Arabidopsis thaliana]
GA	GA20ox	Cluster-8921.27053	-1.6012	0.00059143	0.010031	down	gibberellin 20 oxidase 2-like [Musa acuminata]
		Cluster-8921.93746	3.3393	9.5496E-06	0.00031221	up	Gibberellin 20 oxidase 1-B

	GA2ox	Cluster-8921.54748	1.7652	0.0011515	0.017139	up	gibberellin 2-beta-dioxygenase 8 [Ricinus communis]
		Cluster-8921.53834	1.6502	1.62E-15	4.07E-13	up	gibberellin 2-beta-dioxygenase [Ananas comosus]
		Cluster-8921.39441	3.1589	6.86E-13	1.14E-10	up	gibberellin 2-beta-dioxygenase 8 [Elaeis guineensis]
		Cluster-8921.69332	1.7316	1.02E-09	9.36E-08	up	PREDICTED: gibberellin 2-beta-dioxygenase 1-like [Musa acuminata subsp. malaccensis]
		Cluster-8921.87643	3.1597	1.64E-08	0.00000115	up	Gibberellin 2-beta-dioxygenase 5 [Oryza sativa subsp. japonica]
		Cluster-8921.87642	3.6489	5.11E-08	0.00000317	up	gibberellin 2-beta-dioxygenase 8 isoform X1 [Prunus avium]
SA	ICS	Cluster-8921.78302	-1.0952	0.0022101	0.028904	down	isochorismate synthase 2 [Phoenix dactylifera]
	4CL	Cluster-8921.48543	2.5816	1.9937E-13	3.5941E-11	up	probable 4-coumarate--CoA ligase 2 [Elaeis guineensis]

	Cluster-8921.59093	2.0036	4.7031E-09	3.7367E-07	up	4-coumarate-CoA ligase [Cinnamomum micranthum f. kanehirae]
	Cluster-8921.56507	-6.4049	0.000013205	0.00041283	down	4-coumarate--CoA ligase-like 1 isoform X1 [Sesamum indicum]
PAL	Cluster-8921.75594	2.1203	0.00082448	0.013088	up	phenylalanine ammonia lyase [Cunninghamia lanceolata]
