# Supplementary material for: Endogenous Hormone Levels and Transcriptomic Analysis Reveal the Mechanisms of Bulbil Initiation in Pinellia ternata
Source: Int J Mol Sci. 2024 Jun 3;25(11):6149. doi: 10.3390/ijms25116149 (PMC11173086; doi:10.3390/ijms25116149)
Supplement: Supplementary file 1 [file ijms-25-06149-s001.zip › Sup.Table S7.pdf]

Sup.Table S7 The DEGs information of hormone signaling

hormone	Gene name	Gene_ID	log2FoldChange	pval	padj	up or down	Description
	AUX1	cluster-8921.41248	1.8125	0.0021532	0.028316	up	auxin transporter-like protein 3 [Musa acuminata]
		cluster-8921.63362	-1.3278	0.00058676	0.0099727	down	auxin-induced protein 22C [Ziziphus jujuba]
		cluster-8921.60258	-1.1161	0.0019395	0.026066	down	auxin-responsive protein IAA12-like protein [Cinnamomum micranthum]
	AUX/IAA						
		cluster-8921.18389	-3.645	0.0024285	0.031125	down	auxin-responsive protein IAA [Oryza sativa]
Auxin							
		cluster-8921.57684	-1.4974	0.00011754	0.0026349	down	auxin-responsive protein IAA30-like [Ananas comosus]
		cluster-8921.17746	2.907	0.0036897	0.043087	up	auxin response factor 11 [Elaeis guineensis]
	ARF	cluster-8921.38353	2.3922	0.00000407	0.00014926	up	auxin response factor 11 [Elaeis guineensis]
		cluster-8921.86728	-2.7616	0.00014487	0.0031374	down	AUX/IAA protein [Cinnamomum micranthum]

	cluster-13514.0	3.8899	0.0021868	0.028683	up	SAUR family protein [Ensete ventricosum]
	cluster-8921.47148	1.6652	0.0037384	0.043554	up	auxin-responsive protein SAUR32-like [Musa acuminata]
	cluster-8921.50983	1.5723	0.00013704	0.002998	up	auxin-induced protein 15A [Nelumbo nucifera]
	cluster-8921.73014	1.2707	0.001003	0.015351	up	auxin-induced protein 15A [Nelumbo nucifera]
SAUR	cluster-8921.52060	3.5967	0.00000807	0.00026918	up	Auxin-induced protein [Cinnamomum micranthum]
	cluster-8921.89074	1.7964	0.0014217	0.020393	up	auxin-responsive protein SAUR36-like [Phoenix dactylifera]
	cluster-8921.59122	-1.4607	0.0000535	0.001353	down	auxin-responsive protein SAUR32-like [Ziziphus jujuba]
	cluster-8921.3740	-4.0089	0.0043196	0.048662	down	Auxin-induced protein X10A [Glycine max]
	cluster-8921.104731	-5.472	0.00080145	0.012803	down	small auxin-up RNA [Helianthus annuus]

		cluster-8921.94159	-2.0484	0.00036441	0.0067719	down	auxin-responsive protein SAUR71 [Nelumbo nucifera]
		cluster-8921.37081	-1.4221	0.00000137	0.0000572	down	Auxin-responsive protein SAUR32 [Arabidopsis thaliana]
		cluster-8921.88733	-1.0128	0.00026453	0.0051944	down	auxin-responsive protein SAUR32 [Eutrema salsugineum]
		cluster-8921.80561	-2.029	0.00000661	0.00022679	down	protein Ccrd_025029 [Cynara cardunculus]
	GH3	cluster-8921.94465	3.4861	0.00000492	0.00017608	up	GH3-like hormone conjugating enzyme [Trema orientale]
CTK	CRE1	cluster-8921.49077	1.3627	0.0039466	0.045423	up	histidine kinase 4 [Elaeis guineensis]
		cluster-8921.24392	6.1414	0.00000961	0.00031355	up	histidine kinase 5 [Asparagus officinalis]
	AHP	cluster-8921.73356	-1.3821	0.00013536	0.0029662	down	pseudo histidine-containing phosphotransfer protein 5-like [Phoenix dactylifera]
		cluster-8921.19182	-2.2722	0.00000532	0.00018793	down	pseudo histidine-containing phosphotransfer protein 2-like [Phoenix dactylifera]

	B-ARR	cluster-8921.85010	1.0362	0.00037671	0.0069597	up	two-component response regulator ORR26-like [Phoenix dactylifera]
	A-ARR	cluster-8921.41617	1.1665	0.00077192	0.012428	up	Two-component response regulator ORR10 [Oryza sativa]
		cluster-8921.67255	1.1497	0.000056	0.001411	up	Signal transduction response regulator [Macleaya cordata]
ABA	PP2C	cluster-8921.50405	3.2325	0.0000452	0.0011742	up	probable protein phosphatase 2C 53 [Musa acuminata]
		cluster-8921.57559	1.1822	0.000046	0.0011892	up	protein phosphatase 2C [Asparagus officinalis]
	SnRK2	cluster-8921.54876	-3.0371	0.0000298	0.00082601	down	serine/threonine-protein kinase [Saccharum hybrid]
	ABF	cluster-8921.53684	3.0927	0.0010966	0.016473	up	bZIP transcription factor TRAB1-like [Ananas comosus]
		cluster-8921.4832	-3.5051	0.00011242	0.0025353	down	protein ABSCISIC ACID-INSENSITIVE 5 [Elaeis guineensis]
JAs	COI1	cluster-8921.62063	-1.0136	0.0020038	0.026733	down	coronatine-insensitive protein homolog 1a [Elaeis guineensis]

	JAZ	cluster-8921.63700	1.397	3.98E-08	0.00000254	up	protein TIFY 6b-like isoform [Phoenix dactylifera]
		cluster-8921.64422	1.3478	0.000000211	0.0000112	up	protein TIFY 10a-like [Musa acuminata subsp]
		cluster-8921.51347	3.8107	1.14E-10	1.26E-08	up	protein TIFY 9-like [Manihot esculenta]
		cluster-8921.69647	1.3083	0.000000213	0.0000112	up	Tify [Macleaya cordata]
	MYC2	cluster-8921.53089	1.0984	0.0035487	0.041821	up	transcription factor MYC2-like [Musa acuminata]
SA	NPR1	cluster-8921.67167	1.3651	1.11E-11	1.52E-09	up	BTB/POZ domain and ankyrin repeat-containing protein NPR5 [Elaeis guineensis]
		cluster-8921.55488	1.3566	0.0000296	0.00082114	up	regulatory protein NPR1 [Salvia splendens]
		cluster-8921.48039	2.6706	1.25E-14	2.79E-12	up	BTB/POZ-like [Macleaya cordata]
	TGA	cluster-8921.48480	1.5116	0.00088	0.013778	up	transcription factor TGA2.2 [Elaeis guineensis]
		cluster-8921.81164	1.9885	0.000000641	0.0000296	up	transcription factor TGA2

							[Cinnamomum micranthum]
		cluster-8921.32229	-1.6467	0.00014682	0.0031762	down	pathogenesis-related protein 1-like [Musa acuminata]
	PR-1	cluster-8921.57094	-5.2422	9.94E-09	0.000000729	down	pathogenesis-related protein 1-like [Musa acuminata]
		cluster-8921.57095	-4.3655	0.000000194	0.0000103	down	pathogenesis-related protein 1-like [Musa acuminata]
		cluster-8921.86409	2.6255	0.00088803	0.013888	up	DELLA protein RHT-1-like [Phoenix dactylifera]
	DELLA	cluster-8921.82815	2.9563	1.42E-10	1.54E-08	up	DELLA protein GAI-like protein [Cinnamomum micranthum]
GA		cluster-8921.82816	2.7947	0.000000244	0.0000127	up	DELLA protein RHT-1-like [Phoenix dactylifera]
	TF	cluster-8921.51044	-1.315	0.00000155	0.0000641	down	transcription factor PIF4 isoform [Elaeis guineensis]
		cluster-8921.75269	-1.2876	0.00038209	0.0070408	down	transcription factor PIF4-like protein isoform [Cinnamomum micranthum]

cluster-8921.67736	-1.1314	0.00000373	0.00013912	down	transcription factor PIF4-like isoform [Asparagus officinalis]
cluster-8921.62359	-1.3761	0.000000795	0.0000357	down	transcription factor phytochrome interacting factor-like 13-like [Phoenix dactylifera]
cluster-8921.81801	1.8852	0.00000525	0.00018595	up	transcription factor phytochrome interacting factor-like 15 [Elaeis guineensis]
cluster-8921.60334	2.2192	3.42E-09	0.000000281	up	transcription factor PIF3 isoform [Nelumbo nucifera]
